# A Sustainable Synthesis of Novel 2-(3,4-Disubstituted phenyl)benzoxazole Derivatives and Their Antiproliferative and Antibacterial Evaluation

**DOI:** 10.3390/molecules30081767

**Published:** 2025-04-15

**Authors:** Anja Rakas, Leentje Persoons, Dirk Daelemans, Dajana Kučić Grgić, Tatjana Gazivoda Kraljević

**Affiliations:** 1 Department of Organic Chemistry, Faculty of Chemical Engineering and Technology, University of Zagreb, Marulićev Trg 20, 10000 Zagreb, Croatia; arakas@fkit.unizg.hr; 2 Molecular Genetics and Therapeutics in Virology and Oncology Research Group, Department of Microbiology, Immunology and Transplantation, Rega Institute for Medical Research, KU Leuven, 3000 Leuven, Belgium; leentje.persoons@kuleuven.be (L.P.); dirk.daelemans@kuleuven.be (D.D.); 3 Department of Industrial Ecology, Faculty of Chemical Engineering and Technology, University of Zagreb, Marulićev Trg 19, 10000 Zagreb, Croatia; dkucic@fkit.unizg.hr; 4 Department for Packaging, Recycling and Environmental Protection, University North, Trg dr. Žarka Dolinara 1, 48000 Koprivnica, Croatia

**Keywords:** benzoxazole, sustainable synthesis, antiproliferative activity, antibacterial evaluation

## Abstract

This study describes the synthesis of *O*-alkylated benzaldehydes **1**–**8**, Schiff bases **9**–**28**, and benzoxazole derivatives **29**–**48** using microwave, ultrasound, and mechanochemical reactions, as well as reactions in deep eutectic solvents in excellent yields, and their antiproliferative and antibacterial activities. The in vitro evaluation of antiproliferative activity for the newly synthesised benzoxazole derivatives **29**–**48** against a diverse panel of human cancer cell lines, such as LN-229, Capan-1, HCT-116, NCI-H460, DND-41, HL-60, K-562, and Z-138 demonstrated that the majority of these benzoxazole derivatives displayed promising anticancer activity, particularly against non-small cell lung cancer (NSCLC) cells (NCI-H460). Notably, several derivatives showed enhanced activity compared to the included reference drug, etoposide. Considering the influence of substituents at position 5 of the benzoxazole ring and positions 3 and 4 of the phenyl ring on the antiproliferative activity, it is evident that derivatives **41**–**48** bearing a methoxy group at position 3 generally exhibit higher activity compared to compounds **29**–**40**, which lack substitution at position 3. Furthermore, derivatives substituted at position 4 with a morpholine substituent, as well as those with an *N*,*N*-diethyl group, exhibited higher activity compared to other evaluated benzoxazole derivatives. The in vitro antibacterial evaluation against Gram-positive and Gram-negative bacteria revealed that benzoxazole derivative **47** exhibited notable activity, against the Gram-negative bacterium *Pseudomonas aeruginosa* (MIC = 0.25 μg/mL) and the Gram-positive bacterium *Enterococcus faecalis* (MIC = 0.5 μg/mL). The results point out that this class of benzoxazoles can be efficiently synthesized using eco-friendly methods and represent promising candidates for further design and optimization aimed at developing potent antiproliferative agents.

## 1. Introduction

**Figure 1:**
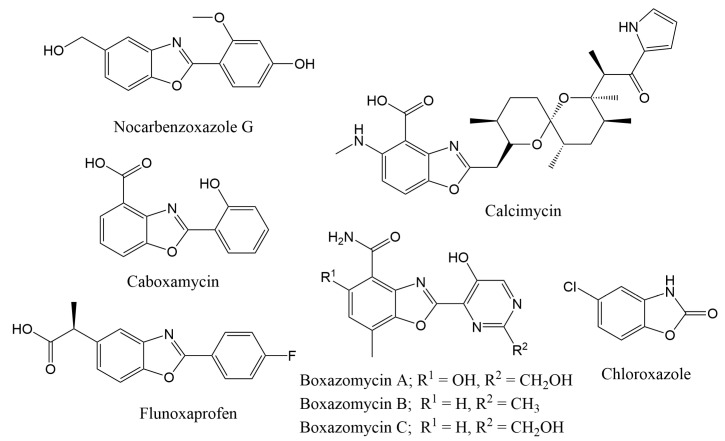
Marketed drugs containing benzoxazole.

Heterocyclic compounds have long been recognized as a cornerstone of medicinal chemistry due to their remarkable structural diversity and biological relevance. Among these, benzoxazole derivatives have emerged as promising candidates for drug discovery [1], exhibiting a wide spectrum of pharmacological activities such as anticancer [2], antimicrobial [3], analgesic [4], anti-inflammatory [5,6], and neuroprotective effects [7]. The benzoxazole skeleton, characterized by a fused oxazole-benzene ring structure, provides a versatile scaffold for the rational design of bioactive molecules [8]. This scaffold is present in many marketed drugs such as the anticancer agent Nocarbenzoxazole G [9], the antibiotics Calcimycin [10] and Caboxamycin [11], the potent broad-spectrum antibiotics boxazomycins A–C [12], the non-steroidal anti-inflammatory drug Flunoxaprofen [13], and the muscle relaxant Chlorzoxazone [14] (Figure 1). Consequently, benzoxazoles continue to play an essential role in drug development, with modifications at specific positions, particularly at position 2, significantly influencing biological activity and enabling targeted interactions with key biomolecular pathways [2,15].

Cancer and infectious diseases remain significant global health challenges, contributing substantially to morbidity and mortality rates worldwide. According to the World Health Organization (WHO), cancer remains one of the leading causes of death globally, accounting for nearly 10 million deaths in 2020. Similarly, antimicrobial resistance (AMR) is an escalating public health crisis, with bacterial infections claiming approximately 1.27 million lives annually, as per recent estimates. These alarming statistics underscore the urgent need for novel therapeutic agents with improved efficacy and specificity to combat these diseases. In the context of anticancer therapy, benzoxazole derivatives have demonstrated potent activity against various cancer cell lines through mechanisms such as DNA intercalation [16,17,18], enzyme inhibition, and disruption of cellular signalling pathways. Inspired by the structures of marketed drugs, 2-arylbenzoxazoles with promising anticancer profiles were synthesized and identified as inhibitors of target proteins involved in anticancer activity. Specifically, benzoxazole derivatives target VEGFR kinase [19,20,21,22,23], and DNA topoisomerase [24,25], and are potent, selective and long-acting inhibitors of PI3Kα, which belongs to the family of lipid kinases present in the plasma membrane [26,27].

Likewise, in the field of antimicrobial therapy, benzoxazole-based compounds have exhibited strong antibacterial activity, including efficacy against drug-resistant strains, making them valuable tools in the fight against antimicrobial resistance (AMR). Synthetic benzoxazole derivatives are widely recognized for their broad-spectrum antimicrobial activity and often exhibit potent antibacterial effects against Gram-positive bacteria such as *Staphylococcus aureus*, *Streptococcus faecalis*, and *Bacillus subtilis*, as well as Gram-negative bacteria including *Escherichia coli*, *Klebsiella pneumoniae*, and *Pseudomonas aeruginosa*. A strong structure–activity relationship (SAR) has been observed for benzoxazole derivatives, highlighting the critical importance of substituents at the 2- and 5-positions of the benzoxazole core. These positions significantly influence antimicrobial potency, especially when both are substituted. Structural modifications at these positions can enhance lipophilicity, electronic properties, and steric compatibility, optimizing interactions with microbial targets such as enzymes, membranes, or other critical cellular components [28]. The biological activity of benzoxazole derivatives can be attributed to their ability to disrupt essential microbial processes. For instance, their lipophilic nature enhances membrane permeability, allowing efficient intracellular accumulation and interaction with key biomolecules. Moreover, these derivatives can inhibit enzymes crucial for cell wall synthesis or DNA replication, thereby exerting a bactericidal effect [29,30]. Given their broad activity spectrum and tunable properties, benzoxazole derivatives are promising candidates for the development of novel antimicrobial agents, particularly in the context of rising resistance to existing drugs. Further exploration of SAR and mechanistic studies can provide valuable insights into optimizing their efficacy and safety profiles for clinical application. In the synthesis of new benzoxazole derivatives, it is desirable to apply green synthetic methodologies that emphasize key aspects such as solvent choice, separation methods, energy efficiency, and waste reduction. These principles support sustainable practices and contribute to a more sustainable world. The goal is to enhance effective and diverse green methodologies, including the use of aqueous media, ionic liquids, solar energy, ultrasonication, mechanochemistry, and bio-based catalysts, all of which play a critical role in the synthesis of bioactive molecules while minimizing environmental and social impacts [30,31]. In addition to the traditional condensation of 2-aminophenol with carboxylic acids or acid halides under strongly acidic conditions, various organometallic-catalyzed reactions have been actively explored for the synthesis of benzoxazole derivatives. These include oxidative coupling of acetals/imines, cross-coupling of amides, arylation, one-pot domino cross-coupling, multi-component processes, tandem oxidation, C–C triple bond cleavage, and dehydrogenative coupling [32]. A major drawback of current methods for synthesizing derivatives is their reliance on inefficient and costly processes. These reactions often require extreme conditions and expensive catalysts, raising toxicity concerns due to the presence of toxic heavy metal impurities and transition metal-bound ligands that may remain in drug intermediates. These challenges can be addressed by adopting more efficient and cost-effective synthetic approaches, such as transition metal-free catalysis, to produce benzoxazole derivatives under milder conditions [1,33].

Based on the findings that the most important positions for biological activity are the substitutions at the C-2 and C-5 positions of the benzoxazole ring, we designed and synthesized derivatives substituted at position 5 of the benzoxazole ring with either bromine or chlorine and at position 2 with a benzene ring that is further substituted at position 3 or 4. This study explores the sustainable synthesis of 2-arylbenzoxazoles and the evaluation of 2-arylbenzoxazole derivatives and their potential as antiproliferative and antibacterial agents.

## 2. Results and Discussion

### 2.1. Chemistry

**Scheme 1:**
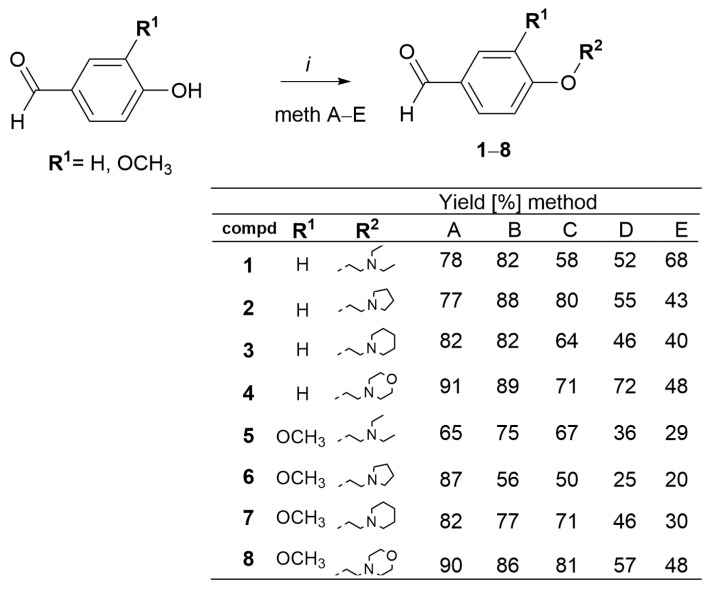
Reagents and conditions: (i) K_2_CO_3_/ACN/appropriate alkyl halide; A: conventional method 5 h, reflux; B: microwave-assisted 2 h, 600 W, 30 °C; C: ultrasound-assisted 2 h, 50 °C; D: mechanochemical 2 h, rt; E: reaction in DES, 2 h, 40 °C.

Benzoxazole derivatives **29**–**48** were prepared through a three-step process, starting with the alkylation of 4-hydroxy benzaldehyde, followed by a condensation reaction with 2-aminophenol, and concluding with the cyclocondensation of Schiff bases. To optimize the reaction conditions and incorporate eco-friendly synthetic methodologies, each step was performed using various approaches, including conventional methods, microwave- and ultrasound-assisted synthesis, mechanochemical reactions, and reactions in deep eutectic solvents (DES). In the first step, 4-*O*-alkylated derivatives of benzaldehyde **1**–**8**, required for the synthesis of Schiff bases **9**–**28** as precursors for benzoxazole cyclization, were prepared through the alkylation of 4-hydroxybenzaldehide or 4-hydroxy-3-methoxybenzaldehide with appropriate haloalkylating reagents in the presence of K_2_CO_3_ as a base (Scheme 1). This alkylation reaction was performed using conventional, microwave- and ultrasound-assisted synthesis, mechanochemical reactions, and reactions in DES. The results indicated that while conventional and microwave-assisted synthesis produced the highest and comparable yields for the *O*-alkylation reaction, the microwave-assisted method was carried out in a shorter time and at a lower temeperature, making it the most efficient among the applied synthetic methods.

**Scheme 2:**
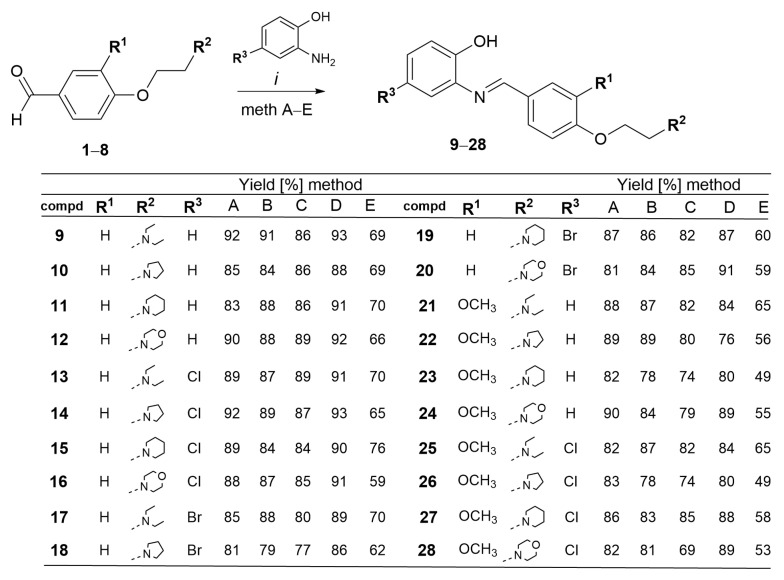
Reagents and conditions: (i) ZnO_NP_/ EtOHabs; A: conventional method, 1 h, rt; B: microwave-assisted, 5 min, 600 W, 30 °C; C: ultrasound-assisted, 5 min, 50 °C; D: mechanochemical, 5 min, 14 Hz; E: reaction in DES, 5 min, 40 °C.

To explore the possibilities of eco-friendly synthesis of 2-arylbenzoxazole derivatives [34,35,36,37], we investigated alternative reaction conditions on a model reaction, testing reagents such as molecular iodine [38] and polyphosphoric acid [39]. Unfortunately, these conditions resulted in low yields. Additionally, we attempted a one-pot reaction using ZnO nanoparticles in ethanol [40], but the yields remained low, and the reaction predominantly formed only the Schiff base. Given these challenges, we decided to isolate the Schiff base and proceed with the cyclocondensation reaction using NaCN without the use of external metal co-catalysts and bases. Therefore, the condensation reaction of 4-*O*-alkylated benzaldehyde derivatives **1**–**8** with the corresponding 2-aminophenol in the presence of ZnO nanoparticles was carried out, resulting in the synthesis of Schiff bases **9**–**28** (Scheme 2). The yields presented in Scheme 2 indicate that applied microwave, ultrasound, and mechanochemical reactions, except for reactions in DES, provide excellent yields comparable to conventional synthesis. However, their main advantage is the significant reduction in reaction time from 1 h to 5 min. These methods can be effectively and widely applied in the synthesis of similar derivatives. Although the yields obtained using DES are somewhat lower, they can still be considered satisfactory.

**Scheme 3:**
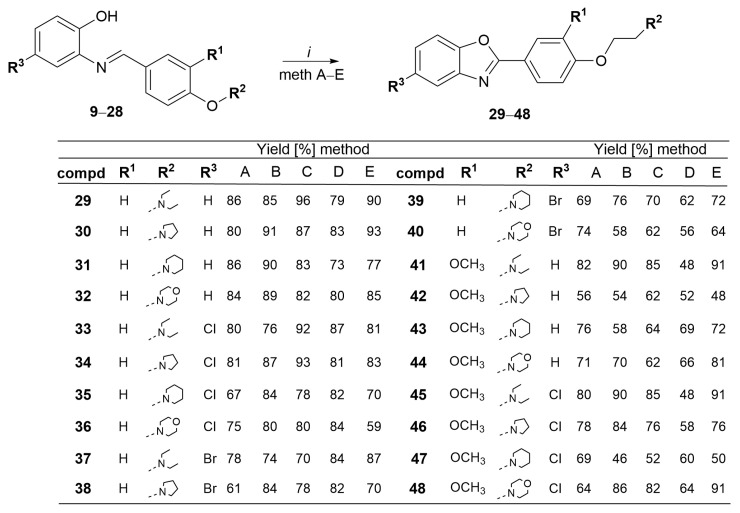
Reagents and conditions; (i) NaCN/DMF/O_2_; A: conventional method, 7–9 h, rt; B: microwave-assisted, 3 h, 600 W, 50 °C; C: ultrasound-assisted, 2 h, 50 °C; D: mechanochemical, 2 h, 14 Hz; E: reaction in DES, 3 h, 40 °C.

The target 2-(3,4-disubstituted phenyl)benzoxazole derivatives **29**–**48** were synthesized via a cyclocondensation reaction of Schiff bases **9**–**28** with NaCN, applying both conventional and sustainable synthetic methods (Scheme 3). The benzoxazole derivatives were predominantly prepared with high yields using methods B–E, comparable to and mostly higher than those obtained with conventional synthesis. The main advantage of the applied sustainable methods is the significant reduction in reaction time, which proved to be superior to conventional methods. Therefore, these cyclizations of Schiff bases into benzoxazole derivatives can be effectively applied in the synthesis of various classes of compounds with different substitutions on the benzoxazole ring.

**Scheme 4:**
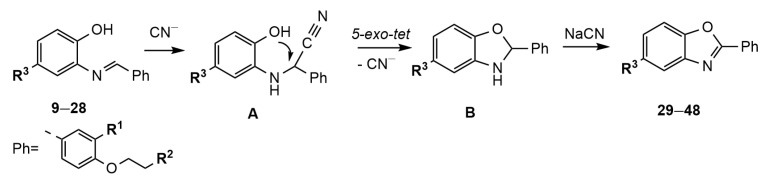
Proposed reaction mechanism for the synthesis of 2-arylbenzoxazoles **29**–**48**.

The proposed reaction mechanism for the synthesis of benzoxazoles **29**–**48** via aerobic oxidation is analogous to the previously reported synthesis of 2-substituted benzoxazole derivatives [41]. In the reaction of imines **9**–**28** with sodium cyanide, intermediate **A** is generated, whose lone pair on the oxygen atom can be properly oriented towards the C–CN bond, which then undergoes a *5-exo-tet* cyclization to form benzoxazoline **B**, followed by oxidation catalyzed by cyanide to yield the desired 2-arylbenzoxazoles **29**–**48** (Scheme 4).

**Figure 2:**
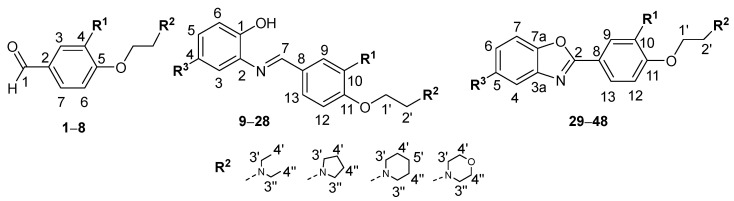
Structures of benzaldehyde derivatives **1**–**8**, Schiff bases **9**–**28** and 2-arylbenzoxazoles **29**–**48** with numbered atoms.

The structures of the prepared Shiff bases **9**–**28** and 2-arylbenzoxazole derivatives **29**–**48** were confirmed by ^1^H- and ^13^C-NMR spectroscopy. The assignment of the ^1^H-NMR spectra was performed based on chemical shifts, signal intensity, resonance multiplicity and H-H coupling constants in accordance with the atom numbering shown in Figure 2, while the ^13^C-NMR/APT spectra were assigned based on chemical shifts, except of compounds **15**, **19**, **21** and **24**. The details of the structural characterization are provided in the Experimental section and Supplementary Materials. The ^1^H-NMR spectra of Schiff bases 9–28 exhibit a characteristic signal at ~8.65 ppm, corresponding to the imine functionality (N=CH-7) along with the expected number of signals in both the aromatic and aliphatic regions which correspond to the protons of the phenyl rings as well as those of the aminoalkyl substituents. The ^13^C-NMR/APT spectra show signals for the imine carbon N=C-7 at ~161 ppm, signals for the carbons of the phenyl rings in the aromatic region, and additional signals in the aliphatic region corresponding to the aminoalkyl substituent. On the other hand, in the ^1^H-NMR spectra of 2-arylbenzoxazoles **29**–**48**, signals for the hydroxyl and imine protons are absent, while in the ^13^C-NMR or APT spectra, signals corresponding to the benzoxazole ring carbons C-2 at ~162 ppm, C-7a at ~150 ppm and C-3a at ~142 ppm are present.

### 2.2. In Vitro Antiproliferative Activity of Benzoxazole Derivatives ***29***–***48***

The in vitro antiproliferative effects of the newly synthesized benzoxazole derivatives **29**–**48** were evaluated against various human cancer cells: LN-229 (glioblastoma), Capan-1 (pancreatic adenocarcinoma), HCT-116 (colorectal carcinoma), NCI-H460 (lungcarcinoma), DND-41 (acute lymphoblastic leukemia), HL-60 (acute myeloid leukemia), K-562 (chronic myeloid leukemia), and Z-138 (non-Hodgkin lymphoma), with etoposide and nocodazole used as reference compounds (Table 1).

The results, presented in Table 1 as IC_50_ values (50% inhibitory concentrations), revealed that the majority of synthesised benzoxazole derivatives displayed promising anticancer activity. It is known that substitution of the 5-position of benzoxazole with a halogen atom, hydroxyl or methyl group, as well as substitution at the 2-position, especially with aryl substituents, can lead to enhanced antiproliferative activity [2]. Detailed analysis indicated that all tested benzoxazole derivatives, except compounds **31** and **34**, showed pronounced to moderate antiproliferative activity against the non-small cell lung cancer (NSCLC) cell line NCI-H460, in the range of 0.4–41.6 μM.

Notably, compounds **30** (IC_50_ = 1.7 μM), **33** (IC_50_ = 1.1 μM), **36** (IC_50_ = 1.3 μM), **39** (IC_50_ = 3.8 μM), **40** (IC_50_ = 0.4 μM), **43** (IC_50_ = 1.8 μM) and **45**–**47** (IC_50_ = 0.9 μM, 1.1 μM, 1.3 μM, respectively) exhibited more pronounced activity compared to the included reference drug etoposide (IC_50_ = 6.1 μM). Moreover, compounds **36**, **43**, **45** and **46** also displayed promising antiproliferative activities against other solid tumour cell lines, namely HCT-116 and LN229 cell lines (IC_50_ = 2.2–14.3 μM). Compound **36**, substituted with chlorine at position 5 of the benzoxazole ring and a morpholine ring at position 4 of the benzene substituent, also showed pronounced activity against Capan-1 (IC_50_ = 2.0 μM) and HCT-116 (IC_50_ = 5.7 μM), and against LN 229 (IC_50_ = 2.2 μM) its antiproliferative activity surpassed the activity of etoposide (IC_50_ = 3.7 μM). Furthermore, 5-chlorobenzoxazole derivative **45**, substituted on the benzene ring with an *N*,*N*-diethyl at position 4 and a methoxy group at position 3, exhibited enhanced antiproliferative activity against HCT-116 (IC_50_ = 2.4 μM) and NCI-H460 (IC_50_ = 0.9 μM) in comparison to etoposide (IC_50_ = 3.4 μM, 6.1 μM, respectively). Analyzing the influence of substituents at the 3-position of the phenyl ring on the antiproliferative activity of 5-unsubstituted benzoxazole derivatives, reveals that derivatives **41**–**48** bearing a methoxy group are generally more active than compounds **29**–**40** with an unsubstituted 3-position. An exception is compounds **36** and **40**, which have a morpholine substituent at the 4-position. Compound **36** shows pronounced to moderate activity across all tested cell lines (IC_50_ = 1.3–86.2 μM), while compound **40** exhibits prominent activity against NCI-H460, Z138 and Capan-1 (IC_50_ = 0.4 μM, 11.2 μM, 15.7 μM, respectively) Among the tested compounds **29**, **33**, **37**, **41** and **45** containing an *N*,*N*-diethyl substituent at position 4 of the benzene ring, compounds **33** and **45** substituted with chlorine in position 5 of the benzoxazole show more enhanced activity compared to compounds **29** and **41** with an unsubstituted benzene ring or with bromine substituted **37**. Investigating the impact of hydrophobic substituents at position 4 of the phenyl ring on antiproliferative activity indicates that derivatives with *N*,*N*-diethyl or morpholine substituents generally exhibit superior activity compared to compounds with pyrrolidine or piperidine substituents. Thus, among all the tested compounds, the most pronounced antiproliferative activity against NCI-H460 was exhibited by benzoxazole derivative **40** with a morpholine substituent (IC_50_ = 0.4 μM) and **45** bearing an *N*,*N*-diethyl substituent (IC_50_ = 0.9 μM).

**Figure 3:**
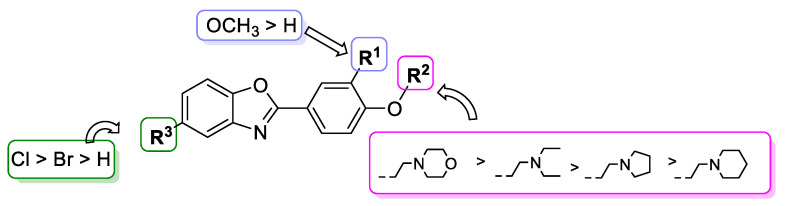
Summary of the structure-activity relationship of the antiproliferative activity of the synthesized benzoxazole derivatives.

Analysis of the antiproliferative activity results has provided valuable insights into the structure-activity relationship. It was found that benzoxazole derivatives bearing a chlorine at position 5 or a methoxy group at position 3 of the phenyl ring, along with an *N*,*N*-diethyl or morpholine substituent at position 4, generally exhibited higher activity compared to compounds lacking substituents at position 5 of benzoxazole and position 3 of the benzene ring (Figure 3).

### 2.3. In Vitro Antibacterial Activity of Benzoxazole Derivatives ***29***–***48***

The synthesized 2-arylbenzoxazole derivatives **29**–**48**, featuring hydrogen, chlorine or bromine atom at the 5-position of the benzoxazole, and at phenyl ring in positions 3 hydrogen or methoxy group and in position 4 *N,N*-diethylaminoethyl or cycloaminoethyl group, were evaluated for their in vitro antibacterial activity against Gram-negative bacteria *Escherichia coli* (*E. coli*), *Pseudomonas aeruginosa* (*P. aeruginosa*) and *Klebsiella pneumoniae* (*K. pneumoniae*) and Gram-positive bacteria *Staphylococcus aureus* (*S. aureus*) and *Enterococcus faecalis* (*E. faecalis*). The minimum inhibitory concentration (MIC) presented in Table 2 is expressed in micrograms per milliliter (μg/mL) and represents the lowest concentration that inhibits visible microbial growth following overnight incubation. To assess the relative potency of the tested benzoxazole derivatives, standard antibiotics ceftazidime (CAZ) and ciprofloxacin (CIP) served as reference controls.

The majority of the tested compounds did not exhibit antibacterial activity. However, benzoxazole derivative **47**, substituted at position 2 with a 4-(piperidinethoxy)phenyl unit, showed pronounced activity specifically against both the Gram-negative bacterium *P. aeruginosa* (MIC = 0.25 μg/mL) and the Gram-positive bacterium *Enterococcus faecalis* (MIC = 0.5 μg/mL). Additionally, benzoxazole derivative **29**, bearing a phenyl ring with an *N*,*N*-diethylethoxy substituent, showed significant activity against *E. faecalis* (MIC = 8 μg/mL), and exhibited more pronounced activity compared to the standard antibiotics CAZ and CIP.

Heterocycles such as benzoxazoles typically exert their antimicrobial effects through intercalation into bacterial membranes, thereby disrupting membrane integrity. This membrane destabilization results in the leakage of essential ions, metabolites, and macromolecules, which ultimately causes bacterial cell death [42]. The significant antibacterial activity of compound **47** against both Gram-negative and Gram-positive bacteria is likely due to its capacity to effectively target and disrupt the membranes of both bacterial types. The substitution patterns at positions 3, 4, and 5 of the benzoxazole ring likely enhance the compound’s interaction with lipid-rich bacterial membranes, thus optimizing its membrane-disruptive activity.

The selective antibacterial activity of compound **47** against Gram-positive and Gram-negative bacteria can be attributed to the distinct structural differences in their cell envelopes. Gram-positive bacteria, such as *Enterococcus faecalis*, lack an outer membrane, which allows for more efficient interaction with the peptidoglycan layer. In contrast, Gram-negative bacteria, like *Pseudomonas aeruginosa*, possess a protective outer membrane that acts as a barrier to many antimicrobial agents [43]. However, the lipophilic nature of compound **47** may facilitate its penetration through the outer membrane, enabling it to target the underlying peptidoglycan layer or other intracellular structures, thus overcoming the permeability barrier.

In addition to their membrane-disrupting effects, certain benzoxazole derivatives, especially those bearing specific substituents, have been shown to interact with bacterial enzymes involved in crucial cellular processes such as cell wall biosynthesis and DNA replication. For instance, these compounds may inhibit enzymes like DNA gyrase and topoisomerase IV, which are vital for bacterial DNA replication and repair [25,44]. The observed antibacterial effects of compound **47** against *Enterococcus faecalis* and *Pseudomonas aeruginosa* may therefore also result from the inhibition of these enzymes, further contributing to the overall antimicrobial activity and subsequent bacterial cell death.

## 3. Materials and Methods

All chemicals and solvents for the synthesis of compounds were purchased from commercial suppliers Sigma-Aldrich ((St. Louis, MO, USA), Alfa Aesar (Haverhill, MA, USA) and Fisher Scientific International Fisher Scientific International (Pittsburg, PA, USA) and were used without additional purification.

For proliferation assays, the human cancer cell lines LN-229, Capan-1, HCT-116, NCI-H460, DND-41, HL-60, K-562 and Z-138, were acquired from the American Type Culture Collection (ATCC, Manassas, VA, USA). Culture media were purchased from Gibco (Gibco Life Technologies, Merelbeke, Belgium) and supplemented with 10% fetal bovine serum (HyClone, Cytiva, Marlborough, MA, USA).

To assess antibacterial activities, bacterial cultures of Gram-negative bacteria *Escherichia coli* (ATCC 25922), *Pseudomonas aeruginosa* (ATCC 9027) and *Klebsiella pneumoniae* (ATCC 27736), and Gram-positive bacteria *Staphylococcus aureus* (ATCC 25923) and *Enterococcus faecalis* (ATCC 29212) were taken from the collection of the Department of Industrial Ecology, University of Zagreb Faculty of Chemical Engineering and Technology. The optical density measurement was carried out using a spectrophotometer Hach, Model DR/2400, Ames, IA, USA.

The monitoring of the progress of the reaction was carried out by thin layer chromatography (TLC) on pre-coated silica gel plates 60F254 (Merck, Darmstadt, Germany).

Column chromatography (CC) was performed on silica gel (Fluka, Buchs, Switzerland, 0.063–0.2 nm), and the glass columns were filled under the influence of gravity, solvent mixture CH_2_Cl_2_:CH_3_OH in the appropriate ratio was used as the eluent. UV light with a wavelength of 254 was used for the detection of isolated components.

Flash chromatography was conducted using Interchim Puri Flash 430 (Montluçon, France).

^1^H- and ^13^C-NMR/APT spectra were recorded on Bruker Avance 300 or 600 MHz spectrometer (Bruker Biospin, Rheinstetten, Germany) using standard pulse sequences. All experiments were recorded in deuterated dymethyl sulfoxide (DMSO-*d*_6_) at 298 K. Chemical shifts (δ) in ^1^H-NMR and ^13^C-NMR/APT spectra are expressed in ppm units relative to tetramethylsilane as the internal standard (TMS, δ = 0.0 ppm), and coupling constants (*J*) in hertz (Hz). Individual resonances were assigned based on their chemical shifts, signal intensities, signal multiplicity and H-H coupling constants.

Melting points were determined with a Koffler hot stage microscope (Reichert, Wien, Austria) and were uncorrected.

Elemental analysis was performed using a PerkinElmer 2400 Series II CHNS analyser (Waltham, MA, USA).

HPLC analysis was performed using Agilent Technologies 1290 Infinity II coupled to an Agilent Technologies 6120 Quadrupole LC/MS mass spectrometer (Santa Clara, CA, USA).

Microwave-assisted reactions were performed in a Milestone start S microwave oven (Sorisole, Italy) using glass cuvettes at 600 W.

Ultrasound-assisted reactions were performed in a Bandelin-Sonorex digiplus DL 1028 H (Berlin, Germany) with a nominal power of 1200 W and a frequency of 35 kHz.

Mechanochemical reactions were performed using an IST400 ball mill (InSolido Technologies, Zagreb, Croatia) at a frequency of 14 Hz.

### 3.1. Chemical Synthesis

#### 3.1.1. General Procedure for the Synthesis of 4-*O*-Alkylated Benzaldehyde Derivatives **1**–**8**

Method A: To a solution of the appropriate benzaldehyde (1 eq, 1.64 mmol or 1.31 mmol) in acetonitrile (CH_3_CN) (8 mL) potassium carbonate (K_2_CO_3_) (2 eq, 3.28 mmol or 2.62 mmol) was added. The reaction mixture was stirred for 1 h at room temperature. Then, the appropriate alkyl halide (1 eq, 1.64 mmol or 1.31 mmol) was added. The reaction mixture was stirred at reflux for 5 h. The progress of the reaction was monitored by TLC. After completion of the reaction, the reaction mixture was filtered and the solvent was evaporated under reduced pressure to dryness. The crude product was purified by column chromatography (CH_2_Cl_2_:CH_3_OH = 100:1).

Method B: To a solution of the appropriate benzaldehyde (1 eq, 1.64 mmol or 1.31 mmol) in CH_3_CN (8 mL) was added K_2_CO_3_ (2 eq, 3.28 mmol or 2.62 mmol). The reaction mixture was stirred in a microwave reactor for 35 min at 600 W and 30 °C, then the appropriate alkyl halide (1 eq, 1.64 mmol or 1.31 mmol) was added and the reaction mixture was stirred in a microwave reactor for 2 h at 600 W and 30 °C. The progress of the reaction was monitored by TLC. After completion, the reaction mixture was filtered, the solvent was removed under reduced pressure, and the crude product was purified by column chromatography (CH_2_Cl_2_:CH_3_OH = 100:1).

Method C: To a solution of the appropriate aldehyde (1 eq, 1.64 mmol or 1.31 mmol) in CH_3_CN (8 mL) was added K_2_CO_3_ (2 eq, 3.28 mmol or 2.62 mmol). The reaction mixture was heated in an ultrasonic bath for 1 h at 50 °C, and then the appropriate alkyl halide (1 eq, 1.64 mmol or 1.31 mmol) was added. The reaction mixture was additionally heated in an ultrasonic bath for 2 h at 50 °C. The progress of the reaction was monitored by TLC. After completion, the reaction mixture was filtered and the solvent was evaporated under reduced pressure. The crude product was purified by column chromatography (CH_2_Cl_2_:CH_3_OH = 100:1).

Method D: To a solution of the appropriate aldehyde (1 eq, 1.64 mmol or 1.31 mmol) in CH_3_CN (240 μL) was added K_2_CO_3_ (2 eq, 3.28 mmol or 2.62 mmol). The reaction mixture was ball milled for 1 h at 14 Hz at room temperature, then the appropriate alkyl halide (1 eq, 1.64 mmol or 1.31 mmol) was added and the mixture was milled in a closed reaction system for 2 h at 14 Hz at room temperature. The progress of the reaction was monitored by TLC. After completion, the reaction mixture was dissolved in CH_3_OH, filtered and the solvent was evaporated to dryness. The crude product was purified by column chromatography (CH_2_Cl_2_:CH_3_OH = 100:1).

Method E: DES was prepared by mixing choline chloride (ChCl) and glycerol in a ratio of 1:2 at 40 °C until a viscous clear solution was formed. The appropriate aldehyde (1 eq, 1.64 mmol or 1.31 mmol) was dissolved in DES (10 mL) and K_2_CO_3_ (2 eq, 3.28 mmol or 2.62 mmol) was added. The reaction was then stirred for 1 h at 40 °C and the appropriate alkyl halide (1 eq, 1.64 mmol or 1.31 mmol) was added. The reaction mixture was additionally stirred for 2 h at 40 °C. The reaction was monitored by TLC. After completion, the reaction mixture was extracted with CH_2_Cl_2_: H_2_O. The organic layer was dried over MgSO_4_, then filtered and the solvent was evaporated under reduced pressure. The crude product was purified by column chromatography (CH_2_Cl_2_:CH_3_OH = 100:1).

*4(4-(2-(Diethylamino)ethoxy)benzaldehyde* **1**. Compound **1** was synthesized according to general procedure Section 3.1.1 from 4-hydroxybenzaldehyde (200 mg, 1.64 mmol) and 2-chloro-*N*,*N*-diethylethanamine hydrochloride (236 mg, 1.64 mmol). After purification by column chromatography compound **1** was obtained as a yellowish oil. (Method A: 281 mg, 78%; Method B: 285 mg, 82%; Method C: 211 mg, 58%; Method D: 189 mg, 52%; Method E: 247 mg, 68%). ^1^H-NMR (600 MHz, DMSO-*d*_6_) δ 9.87 (s, 1H, H-1), 7.86 (d, *J* = 8.8 Hz, 2H, H-3,7), 7.13 (d, *J* = 8.7 Hz, 2H, H-4,6), 4.13 (t, *J* = 6.1 Hz, 2H, H-1′), 2.80 (t, *J* = 6.1 Hz, 2H, H-2′), 2.55 (q, *J* = 7.1 Hz, 4H, H-3′,3″), 0.97 (t, *J* = 7.1 Hz, 6H, H-4′,4″). ^13^C-NMR/APT (151 MHz, DMSO-*d*_6_) δ 191.9, 154.0, 143.7, 130.1, 114.7, 67.1, 54.9, 46.0, 12.3.

*4-(2-(Pyrrolidin-1-yl)ethoxy)benzaldehyde* **2**. Compound **2** was synthesized according to general procedure Section 3.1.1 from 4-hydroxybenzaldehyde (200 mg, 1.64 mmol) and 1-(2-chloroethyl)pyrrolidine hydrochloride (279 mg, 1.64 mmol). After purification by column chromatography compound **2** was obtained as a yellowish oil. (Method A: 277 mg, 77%; Method B: 314 mg, 88%; Method C: 287 mg, 80%; Method D: 196 mg, 55%, Method E: 155 mg, 43%). ^1^H NMR (600 MHz, DMSO-*d*_6_) δ 9.87 (s, 1H, H-1), 7.86 (d, *J* = 8.8 Hz, 2H, H-3,7), 7.14 (d, *J* = 8.7 Hz, 2H, H-4,6), 4.19 (t, *J* = 5.8 Hz, 2H, H-1′), 2.81 (t, *J* = 5.8 Hz, 2H, H-2′), 2.53 (m, 4H, H-3′,3″), 1.68 (m, 4H, H-4′,4″). ^13^C-NMR/APT (151 MHz, DMSO-*d*_6_) δ 191.8, 164.0, 130.1, 126.6, 115.9, 67.1, 57.6, 39.9, 23.6.

*4-(2-(Piperidin-1-yl)ethoxy)benzaldehyde* **3**. Compound **3** was synthesized according to general procedure Section 3.1.1 from 4-hydroxybenzaldehyde (200 mg, 1.64 mmol) and 1-(2-chloroethyl)piperidine hydrochloride (300 mg, 1.64 mmol). After purification by column chromatography compound 3 was obtained as a yellowish oil. (Method A: 315 mg, 82%; Method B: 313 mg, 82%; Method C: 246 mg, 64%, Method D: 175 mg, 46% Method E: 153 mg, 40%). 1H-NMR (600 MHz, DMSO-*d*_6_) δ 9.86 (s, 1H, H-1), 7.85 (d, *J* = 8.8 Hz, 2H, H-3,7), 7.12 (d, *J* = 8.7 Hz, 2H, H-4,6), 4.17 (t, *J* = 5.9 Hz, 2H, H-1′), 2.66 (t, *J* = 5.9 Hz, 2H, H-2′), 2.42 (t, *J* = 5.3 Hz, 4H, H-3′,3″), 1.48 (p, *J* = 5.6 Hz, 4H, H-4′,4″), 1.36 (m, 2H, H-5′). 13C-NMR (151 MHz, DMSO-*d*_6_) δ 191.2 (C-1), 163.5, 131.7, 129.5, 115.0, 65.7, 57.1, 54.3, 25.5, 23.9.

*4-(2-Morpholinoethoxy)benzaldehyde* **4**. Compound **4** was synthesized according to general procedure Section 3.1.1 from 4-hydroxybenzaldehyde (200 mg, 1.64 mmol) and 4-(2-chloroethyl)morpholine hydrochloride (305 mg, 1.64 mmol). After purification by column chromatography compound **4** was obtained as a beige oil. (Method A: 285 mg, 91%, Method B: 275 mg, 89%; Method C: 218 mg, 71%, Method D: 223 mg, 72% Method E: 147 mg, 48%). ^1^H-NMR (300 MHz, DMSO-*d*_6_) δ 9.88 (s, 1H, H-1), 7.87 (d, *J* = 8.7 Hz, 2H, H-3,7), 7.14 (d, *J* = 8.7 Hz, 2H, H-4,6), 4.21 (t, *J* = 5.7 Hz, 2H, H-1′), 3.58 (t, *J* = 4.5 Hz, 4H, H-4′,4″), 2.72 (t, *J* = 5.7 Hz, 2H, H-2′), 2.52 (t, *J* = 4.5 Hz, 4H, H-3′,3″). ^13^C-NMR (151 MHz, DMSO-*d*_6_) δ 191.3, 163.4, 131.8, 129.6, 115.0, 66.1, 65.8, 56.8, 53.6.

*4-(2-(Diethylamino)ethoxy)-3-methoxybenzaldehyde* **5**. Compound **5** was synthesized according to general procedure Section 3.1.1 from 4-hydroxy-3-methoxybenzaldehyde (200 mg, 1.31 mmol) and *N*,*N*-diethyl-2-chloroethan-1-amine hydrochloride (225 mg, 1.31 mmol). After purification by column chromatography compound **5** was obtained as a yellowish oil. (Method A: 216 mg, 65%; Method B: 246 mg, 75%; Method C: 222 mg, 67%; Method D: 119 mg, 36%; Method E: 96 mg, 29%). ^1^H-NMR (600 MHz, DMSO-*d*_6_) δ 9.83 (s, 1H, H-1), 7.52 (dd, *J* = 8.2, 1.9 Hz, 1H, H-7), 7.38 (d, *J* = 1.9 Hz, 1H, H-3), 7.18 (d, *J* = 8.2 Hz, 1H, H-6), 4.11 (t, *J* = 6.2 Hz, 2H, H-1′), 3.82 (s, 3H, OCH_3_), 2.81 (t, *J* = 6.2 Hz, 2H, H-2′,2″), 2.55 (q, *J* = 7.1 Hz, 4H, H-3′,3″), 0.96 (t, *J* = 7.1 Hz, 6H, H-4′,4″). ^13^C-NMR/APT (151 MHz, DMSO-*d*_6_) δ 191.3, 153.5, 149.2, 132.7, 129.6, 112.1, 109.7, 67.3, 59.4, 55.5, 53.8, 25.1.

*3-Methoxy-4-(2-(pyrrolidin-1-yl) ethoxy)benzaldehyde* **6**. Compound **6** was synthesized according to general procedure Section 3.1.1 from 4-hydroxy-3-methoxybenzaldehyde (200 mg, 1.31 mmol) and 1-(2-chloroethyl) pyrrolidine hydrochloride (222 mg, 1.31 mmol). After purification by column chromatography compound **6** was obtained as a pale-yellow oil. (Method A: 287 mg, 87%; Method B: 183 mg, 56%; Method C: 164 mg, 50%, Method D: 83 mg, 25%; Method E: 67 mg, 20%). ^1^H-NMR (600 MHz, DMSO-*d*_6_) δ 9.84 (s, 1H, H-1), 7.53 (dd, *J* = 8.2, 1.9 Hz, 1H, H-7), 7.39 (d, *J* = 1.9 Hz, 1H, H-3), 7.19 (d, *J* = 8.3 Hz, 1H, H-6), 4.17 (t, *J* = 6.0 Hz, 2H, H-1′), 3.83 (s, 3H, OCH_3_), 2.81 (t, *J* = 6.0 Hz, 2H, H-2′), 2.52 (m, 4H, H3′,3″), 1.68 (m, 4H, H-4′,4″). ^13^C-NMR/APT (151 MHz, DMSO-*d*_6_) δ 191.3, 153.5, 149.2, 129.6, 126.0, 111.8, 109.6, 67.7, 55.5, 54.1, 54.0, 23.1.

*3-Methoxy-4-(2-(piperidin-1-yl) ethoxy)benzaldehyde* **7**. Compound **7** was synthesized according to general procedure Section 3.1.1 from 4-hydroxy-3-methoxybenzaldehyde (200 mg, 1.31 mmol) and 1-(2-chloroethyl) piperidine hydrochloride (239 mg, 1.31 mmol). After purification by column chromatography compound **7** was obtained as a light-yellow oil. (Method A: 286 g, 82%; Method B: 265 mg, 77%; Method C: 244 mg, 71%; Method D: 159 mg, 46% Method E: 103 mg, 30%). ^1^H-NMR (300 MHz, DMSO-*d*_6_) δ 9.84 (s, 1H, H-1), 7.53 (dd, *J* = 8.3, 1.9 Hz, 1H, H-7), 7.39 (d, *J* = 1.9 Hz, 1H, H-3), 7.20 (d, *J* = 8.3 Hz, 1H, H-6), 4.18 (t, *J* = 6.0 Hz, 2H, H-1′), 3.83 (s, 3H, OCH_3_), 2.70 (t, *J* = 6.0 Hz, 2H, H-2′), 2.45 (t, *J* = 5.3 Hz, 4H, H-3′,3″), 1.49 (t, *J* = 5.6 Hz, 4H, H-4′,4′′), 1.38 (m, 2H, H-5′). ^13^C-NMR/APT (151 MHz, DMSO-*d*_6_) δ 190.2, 152.4, 148.1, 128.5, 124.9, 111.0, 108.5, 66.6, 54.4, 53.0, 52.9, 24.3, 22.0.

*3-Methoxy-4-(2-morpholinoethoxy)benzaldehyde* **8**. Compound **8** was synthesized according to general procedure Section 3.1.1 from 4-hydroxy-3-methoxybenzaldehyde (200 mg, 1.31 mmol) and 4-(2-chloroethyl) morpholine hydrochloride (243 mg, 1.31 mmol). After purification by column chromatography compound **8** was obtained as a brown oil. (Method A: 316 mg, 90%; Method B: 301 mg, 86%; Method C: 281 mg, 81%; Method D: 199 mg, 57% Method E: 166 mg, 48%). ^1^H-NMR (300 MHz, DMSO-*d*_6_) δ 9.84 (s, 1H, H-1), 7.54 (dd, *J* = 8.2, 1.9 Hz, 1H, H-7), 7.40 (d, *J* = 1.9 Hz, 1H, H-3), 7.21 (d, *J* = 8.2 Hz, 1H, H-6), 4.20 (t, *J* = 5.8 Hz, 2H, H-1′), 3.83 (s, 3H, OCH_3_), 3.58 (t, *J* = 4.6 Hz, 4H, H-4′,4″), 2.73 (t, *J* = 5.8 Hz, 2H, H-2′), 2.49 (m, 4H, H-3′,3″). ^13^C-NMR/APT (151 MHz, DMSO-*d*_6_) δ 191.3, 153.6, 149.2, 129.7, 125.0, 112.3, 109.7, 66.4, 56.7, 55.6, 53.6, 53.20.

#### 3.1.2. General Procedure for the Synthesis of Schiff Bases **9**–**28**

Method A: To a solution of benzaldehyde derivatives **1**–**8** (1 eq) and the corresponding 2-aminophenol (1 eq) in absolute ethanol (EtOH_abs_) (5 mL), nanoparticles of zinc (II) oxide (ZnO_NP_) as a catalyst (0.3 eq, 10 mg, 0.12 mmol) was added. The reaction mixture was stirred for 1 h at room temperature. The progress of the reaction was monitored by TLC. After completion of the reaction, the reaction mixture was filtered and the solvent was evaporated to dryness under reduced pressure to obtain the pure compound.

Method B: To a solution of benzaldehyde derivatives **1**–**8** (1 eq) and the corresponding 2-aminophenol (1 eq, 0.45 mmol) in EtOH_abs_ (5 mL), ZnO_NP_ (0.3 eq, 10 mg, 0.12 mmol) was added. The reaction mixture was stirred in a microwave reactor for 5 min at 600 W and 30 °C. The progress of the reaction was monitored by TLC. After completion of the reaction, the reaction mixture was filtered and the solvent was evaporated to dryness under reduced pressure to obtain the pure compound.

Method C: To a solution of benzaldehyde derivatives **1**–**8** (1 eq) and the corresponding 2-aminophenol (1 eq) in EtOH_abs_ (5 mL), ZnO_NP_ (0.3 eq, 10 mg, 0.12 mmol) was added. The reaction mixture was heated in an ultrasonic bath for 5 min at 50 °C. The progress of the reaction was monitored by TLC. After completion of the reaction, the reaction mixture was filtered and the solvent was evaporated to dryness under reduced pressure to obtain the pure compound.

Method D: To a solution of benzaldehyde derivatives **1**–**8** (1 eq) and the corresponding 2-aminophenol (1 eq) in EtOH_abs_ (240 μL), ZnO_NP_ (0.3 eq, 10 mg, 0.12 mmol) was added. The reaction mixture was ground in a ball mill for 5 min at 14 Hz at room temperature. The progress of the reaction was monitored by TLC. After completion, the reaction mixture was dissolved in CH_3_OH, filtered and the solvent was evaporated to dryness to obtain the pure compound.

Method E: DES was prepared by mixing choline chloride (ChCl) and glycerol in a ratio of 1:2 at 40 °C until a clear viscous solution was formed. Benzaldehyde derivatives **1**–**8** (1 eq) were dissolved in DES (10 mL) and ZnO_NP_ (0.3 eq, 10 mg, 0.12 mmol) was added. The reaction mixture was stirred for 5 min at 40 °C. The progress of the reaction was monitored by TLC. After completion, the reaction mixture was extracted with CH_2_Cl_2_:H_2_O. Layers were separated and the organic layer was dried over MgSO_4_, then filtered off and the solvent was evaporated under reduced pressure to obtain the pure compound.

*2-((4-(2-(Diethylamino)ethoxy)benzylidene)amino)phenol* **9**. Compound **9** was synthesized according to general procedure Section 3.1.2 from **1** (100 mg, 0.45 mmol), ZnO_NP_ (10 mg, 0.12 mmol) and 2-aminophenol (49 mg, 0.45 mmol). Compound **9** was obtained as a brown oil. (Method A: 129 mg, 92%; Method B: 128 mg, 91%; Method C: 125 mg, 89%; Method D: 131 mg, 93%; Method E: 98 mg, 69%)**.** ^1^H-NMR (600 MHz, DMSO-*d*_6_) δ 9.21 (s, 1H, OH), 8.62 (s, 1H, N=CH-7), 7.97 (d, *J* = 8.8 Hz, 2H, H-9,13), 7.86 (d, *J* = 8.8 Hz, 1H, H-5), 7.24 (d, *J* = 7.5 Hz, 2H, H-10,12), 7.14 (d, *J* = 8.7 Hz, 1H, H-3), 7.08 (d, *J* = 8.6 Hz, 1H, H-4), 6.89 (d, *J* = 8.6 Hz, 1H, H-6), 4.19 (t, *J* = 5.8 Hz, 2H, H-1′), 2.82 (t, *J* = 5.9 Hz, 4H, H-3′,3″), 2.54 (m, 2H, H-2′), 1.06 (t, *J* = 6.0 Hz, 6H, H-4′,4″). ^13^C-NMR/APT (151 MHz, DMSO-*d*_6_) δ 162.9, 162.1, 150.5, 143.6, 128.5, 127.1, 125.1, 124.1, 119.6, 117.5, 115.9, 113.7, 67.4, 55.2, 50.3, 45.3, 18.8, 12.7. Calcd for C_19_H_24_N_2_O_2_: C, 73.05; H, 7.74; N, 8.97; Found: C, 73.07; H, 7.73; N, 8.95.

*2-((4-(2-(Pyrrolidin-1-yl)ethoxy)benzylidene)amino)phenol* **10**. Compound **10** was synthesized according to general procedure Section 3.1.2 from **2** (100 mg, 0.46 mmol), ZnO_NP_ (10 mg, 0.12 mmol) and 2-aminophenol (50 mg, 0.46 mmol). Compound **10** was obtained as an orange oil. (Method A: 120 mg, 85%; Method B: 119 mg, 84%; Method C: 122 mg, 86%; Method D: 125 mg, 88%; Method E: 96 mg, 69%). ^1^H-NMR (600 MHz, DMSO-*d*_6_) δ 9.87 (s, 1H, OH), 8.62 (s, 1H, N=CH-7), 7.97 (d, *J* = 8.8 Hz, 2H, H-9,13), 7.86 (d, *J* = 8.8 Hz, 1H, H-5), 7.24 (d, *J* = 7.4 Hz, 2H, H-10,12), 7.14 (d, *J* = 8.7 Hz, 1H, H-3), 7.08 (dd, *J* = 8.6, 2.9 Hz, 1H, H-4), 6.89 (d, *J* = 8.6 Hz, 1H, H-6), 4.19 (t, *J* = 5.8 Hz, 2H, H-1′), 2.82 (m, 2H, H-2′), 2.55 (m, 4H, H-3′,3″), 1.69 (m, 4H, H-4′,4″). ^13^C-NMR/APT (151 MHz, DMSO-*d*_6_) δ 161.59, 160.21, 151.08, 142.35, 129.81, 125.60, 120.01, 116.54, 111.52, 55.18, 23.19. Calcd for C_19_H_22_N_2_O_2_: C, 73.52; H, 7.14; N, 9.03; Found: C, 73.50; H, 7.15; N, 9.01.

*2-((4-(2-(Piperidin-1-yl)ethoxy)benzylidene)amino)phenol* **11**. Compound **11** was synthesized according to general procedure Section 3.1.2 from **3** (100 mg, 0.43 mmol), ZnO_NP_ (10 mg, 0.12 mmol) and 2-aminophenol (47 mg, 0.43 mmol). Compound **11** was obtained as a brown oil. (Method A: 115 mg, 83%; Method B: 123 mg, 88%; Method C: 120 mg, 86%; Method D: 126 mg, 91%; Method E: 97 mg, 70%). ^1^H-NMR (600 MHz, DMSO-*d*_6_) δ 8.88 (s, 1H, OH), 8.62 (s, 1H, N=CH-7), 7.97 (d, *J* = 8.7 Hz, 1H, H-9,13), 7.17 (dd, *J* = 7.9, 1.6 Hz, 1H, H-5), 7.07 (d, *J* = 8.8 Hz, 2H, H-10,12), 7.04 (d, *J* = 1.6 Hz, 1H, H-3), 6.89 (d, *J* = 1.3 Hz, 1H, H-4), 6.82 (d, *J* = 1.4 Hz, 1H, H-6), 4.15 (t, *J* = 5.9 Hz, 2H, H-1′), 2.68 (t, *J* = 5.9 Hz, 2H, H-2″), 2.44 (m, 4H, H-3′,3″), 1.51 (m, 4H, H-4′,4″), 1.39 (m, 2H, H-5′). ^13^C-NMR/APT (151 MHz, DMSO-*d*_6_) δ 161.49, 158.82, 151.59, 144.36, 131.00, 127.38, 121.30, 119.30, 116.25, 66.03, 57.25, 54.72, 25.78, 24.30. Calcd for C_20_H_24_N_2_O_2_: C, 74.05; H, 7.46; N, 8.63; Found: C, 74.07; H, 7.45; N, 8.64.

*2-((4-(2-Morpholinoethoxy)benzylidene)amino)phenol* **12**. Compound 12 was synthesized according to general procedure Section 3.1.2 from **4** (100 mg, 0.42 mmol), ZnO_NP_ (10 mg, 0.12 mmol) and 2-aminophenol (46 mg, 0.42 mmol). Compound **12** was obtained as a brown oil. (Method A: 125 mg, 90%; Method B: 122 mg, 88%; Method C: 124 mg, 89%; Method D: 127 mg, 92%; Method E: 91 mg, 66%). ^1^H-NMR (600 MHz, DMSO-*d*_6_) δ 9.23 (s, 1H, OH), 8.63 (s, 1H, N=CH-7), 7.97 (d, *J* = 8.8 Hz, 2H, H-9,13), 7.30 (d, *J* = 2.6 Hz, 1H, H-5), 7.24 (d, *J* = 7.6 Hz, 2H, H-10,12), 7.09 (d, *J* = 2.7 Hz, 1H, H-3), 7.07 (d, *J* = 2.6 Hz, 1H, H-4), 6.89 (dd, *J* = 8.6, 2.6 Hz, 1H, H-6), 4.27 (t, *J* = 5.7 Hz, 2H, H-1′), 3.59 (m, 4H, H-4′,4″), 2.76 (t, *J* = 5.7 Hz, 2H, H-2′), 2.51 (m, 4H, H-3′,3″). ^13^C-NMR/APT (151 MHz, DMSO-*d*_6_) δ 161.6, 160.6, 149.2, 143.3, 131.4, 127.7, 127.1, 126.5, 119.1, 117.8, 113.3, 110.0, 66.6,66.2, 56.7, 54.1.

*4-Chloro-2-((4-(2-(diethylamino)ethoxy)benzylidene)amino)phenol* **13**. Compound **13** was synthesized according to general procedure Section 3.1.2 from **1** (100 mg, 0.45 mmol), ZnO_NP_ (10 mg, 0.12 mmol) and 2-amino-4-chlorophenol (65 mg, 0.45 mmol). Compound **13** was obtained as a brown oil. (Method A: 139 mg, 89%; Method B: 136 mg, 87%; Method C: 140 mg, 89%; Method D: 144 mg, 91%; Method E: 109 mg, 70%). ^1^H-NMR (600 MHz, DMSO-*d*_6_) δ 9.25 (s, 1H, OH), 8.62 (s, 1H, N=CH-7), 7.96 (d, *J* = 8.8 Hz, 2H, H-9,13), 7.23 (d, *J* = 8.6 Hz, 1H, H-5), 7.08 (d, *J* = 2.6 Hz, 1H, H-3), 7.07 (m, 2H, H-10,12), 6.88 (d, *J* = 8.6 Hz, 1H, H-6), 4.11 (t, *J* = 6.1 Hz, 2H, H-1′), 2.81 (t, *J* = 6.1 Hz, 2H, H-2′), 2.57 (q, *J* = 7.2 Hz, 4H, H-3′,3″), 1.06 (t, *J* = 7.0 Hz, 6H, H-4′,4″). ^13^C-NMR/APT (151 MHz, DMSO-*d*_6_) δ 163.4, 161.5, 150.9, 143.3, 139.6, 131.5, 127.4, 125.1, 119.7, 117.4, 115.9, 115.1, 113.2, 67.4, 57.4, 54.2, 48.3, 19.8, 12.7. Calcd for C_19_H_23_ClN_2_O_2_: C, 65.79; H, 6.68; N, 8.08; Found: C, 65.77; H, 6.69; N, 8.06.

*4-Chloro-2-((4-(2-(pyrrolidin-1-yl)ethoxy)benzylidene)amino)phenol* **14**. Compound **14** was synthesized according to general procedure Section 3.1.2 from **2** (100 mg, 0.46 mmol), ZnO_NP_ (10 mg, 0.12 mmol) and 2-amino-4-chlorophenol (65 mg, 0.46 mmol). Compound **14** was obtained as an organic oil. (Method A: 145 mg, 92%; Method B: 140 mg, 89%; Method C: 136 mg, 87%; Method D: 147 mg, 93%; Method E: 102 mg, 65%). ^1^H-NMR (600 MHz, DMSO-*d*_6_) δ 8.93 (s, 1H, OH), 8.62 (s, 1H, N=CH-7), 7.97 (d, *J* = 8.8 Hz, 2H, H-9,13), 7.17 (dd, *J* = 7.9, 1.6 Hz, 1H, H-5), 7.07 (d, *J* = 8.8 Hz, 2H, H-10,12), 7.04 (d, *J* = 1.6 Hz, 1H, H-3), 6.88 (d, *J* = 8.1 Hz, 1H, H-6), 4.16 (t, *J* = 5.9 Hz, 2H, H-1′), 2.82 (t, *J* = 5.9 Hz, 2H, H-2′), 2.54 (m, 4H, H-3′,3″), 1.69 (m, 4H, H-4′,4″). Calcd for C_19_H_21_ClN_2_O_2_: C, 66.18; H, 6.14; N, 8.12; Found: C, 66.16; H, 6.15; N, 8.13.

*4-Chloro-2-((4-(2-(piperidin-1-yl)ethoxy)benzylidene)amino)phenol **15**. Compound* **15** was synthesized according to general procedure Section 3.1.2 from **3** (100 mg, 0.43 mmol), ZnO_NP_ (10 mg, 0.12 mmol) and 2-amino-4-chlorophenol (61 mg, 0.43 mmol). Compound **15** was obtained as a light brown oil. (Method A: 137 mg, 89%; Method B: 134 mg, 84%; Method C: 129 mg, 84%; Method D: 139 mg, 90%; Method E: 117 mg, 76%). ^1^H-NMR (600 MHz, DMSO-*d*_6_) δ 9.25 (s, 1H, OH), 8.66 (s, 1H, N=CH-7), 8.01 (d, *J* = 8.4 Hz, 2H, H-9,13), 7.40 (d, *J* = 8.4 Hz, 2H, H-10,12), 7.32 (d, *J* = 7.6 Hz, 1H, H-5), 7.22 (d, *J* = 2.4 Hz, 1H, H-3), 6.85 (d, *J* = 8.6 Hz, 1H, H-6), 4.27 (t, *J* = 5.8 Hz, 2H, H-1′), 2.72 (m, 2H, H-2′), 2.44 (m, 4H, H-3′,3″), 1.51 (m, 4H, H-4′,4″), 1.38 (p, *J* = 5.9 Hz, 2H, H-5′). Calcd for C_20_H_23_ClN_2_O_2_: C, 66.94; H, 6.46; N, 7.81; Found: C, 66.96; H, 6.47; N, 7.80.

*4-Chloro-2-((4-(2-morpholinoethoxy)benzylidene)amino)phenol* **16**. Compound **16** was synthesized according to general procedure Section 3.1.2 from **4** (100 mg, 0.42 mmol), ZnO_NP_ (10 mg, 0.12 mmol) and 2-amino-4-chlorophenol (61 mg, 0.42 mmol). Compound **16** was obtained as an orange oil. (Method A: 135 mg, 88%; Method B: 133 mg, 87%; Method C: 130 mg, 85%; Method D: 139 mg, 91%; Method E: 90 mg, 89%). ^1^H-NMR (600 MHz, DMSO-*d*_6_) δ 9.24 (s, 1H, OH), 8.67 (s, 1H, N=CH-7), 8.02 (d, *J* = 8.6 Hz, 2H, H-9,13), 7.32 (d, *J* = 8.5 Hz, 1H, H-5), 7.30 (d, *J* = 7.5 Hz, 2H, H-10,12), 7.11 (d, *J* = 1.8 Hz, 1H, H-3), 6.90 (d, *J* = 8.6 Hz, 1H, H-6), 4.26 (t, *J* = 5.7 Hz, 2H, H-1′), 3.59 (t, *J* = 4.7 Hz, 4H, H-4′,4″), 2.75 (t, *J* = 5.7 Hz, 2H, H-2′), 2.49 (m, 4H, H-3′,3″). ^13^C-NMR/APT (151 MHz, DMSO-*d*_6_) δ 163.9, 161.7, 148.9, 143.0, 129.3, 125.8, 125.5, 119.1, 118.2, 115.8, 109.7, 66.1, 65.7, 56.7, 54.3. Calcd for C_19_H_21_ClN_2_O_3_: C, 63.24; H, 5.87; N, 7.76; Found: C, 63.23; H, 5.89; N, 7.77.

*4-Bromo-2-((4-(2-(diethylamino)ethoxy)benzylidene)amino)phenol* **17**. Compound **17** was synthesized according to general procedure Section 3.1.2 from **1** (100 mg, 0.45 mmol), ZnO_NP_ (10 mg, 0.12 mmol) and 2-amino-4-bromophenol (85 mg, 0.45 mmol). Compound **17** was obtained as a brown oil. (Method A: 151 mg, 85%; Method B: 156 mg, 88%; Method C: 143 mg, 80%; Method D: 158 mg, 89%; Method E: 124 mg, 70%). ^1^H-NMR (600 MHz, DMSO-*d*_6_) δ 9.25 (s, 1H, OH), 8.62 (s, 1H, N=CH-7), 7.96 (d, *J* = 8.8 Hz, 2H, H-9,13), 7.23 (d, *J* = 8.6 Hz, 1H, H-5), 7.07 (d, *J* = 7.8 Hz, 2H, H-10,12), 7.06 (d, *J* = 2.1 Hz, 1H, H-3), 6.88 (d, *J* = 8.6 Hz, 1H, H-6), 4.11 (t, *J* = 6.1 Hz, 2H, H-1′), 2.81 (t, *J* = 6.1 Hz, 2H, H-2′), 2.57 (q, *J* = 7.2 Hz, 4H, H-3′,3″), 0.99 (t, *J* = 7.1 Hz, 6H, H-4′,4″). ^13^C-NMR/APT (151 MHz, DMSO-*d*_6_) δ 163.1, 161.3, 150.7, 143.4, 138.2, 131.7, 126.2, 124.7, 119.0, 117.7, 115.3, 114.8, 112.5, 67.7, 54.7, 51.2, 47.3, 19.4, 12.2.

*4-Bromo-2-((4-(2-(pyrrolidin-1-yl)ethoxy)benzylidene)amino)phenol* **18**. Compound **18** was synthesized according to general procedure Section 3.1.2 from **2** (100 mg, 0.46 mmol), ZnO_NP_ (10 mg, 0.12 mmol) and 2-amino-4-bromophenol (86 mg, 0.46 mmol). Compound **18** was obtained as a brown oil. (Method A: 144 mg, 81%; Method B: 141 mg, 79%; Method C: 137 mg, 77%; Method D: 152 mg, 86%; Method E: 109 mg, 62%). ^1^H-NMR (600 MHz, DMSO-*d*_6_) δ 8.99 (s, 1H, OH), 8.60 (s, 1H, N=CH-7), 7.76 (d, *J* = 6.9 Hz, 2H, H-9,13), 7.19 (d, *J* = 8.4 Hz, 1H, H-5), 7.10 (d, *J* = 7.3 Hz, 2H, H-10,12), 7.05 (d, *J* = 1.6 Hz, 1H, H-3), 6.89 (d, *J* = 8.4 Hz, 1H, H-6), 4.14 (t, *J* = 6.0 Hz, 2H, H-1′), 2.82 (t, *J* = 6.0 Hz, 2H, H-2′), 2.54 (m, 4H, H-3′,3″), 1.69 (t, *J* = 3.6 Hz, 4H, H-4′,4″). ^13^C-NMR/APT (151 MHz, DMSO-*d*_6_) δ 159.4, 161.9, 150.4, 148.4, 139.6, 138.1, 128.9, 127.7, 125.3, 124.2, 119.3, 117.3, 115.8, 67.3, 57.7, 54.8, 23.4. Calcd for C_19_H_21_BrN_2_O_2_: C, 58.62; H, 5.44; N, 7.20; Found: C, 58.60; H, 5.46; N, 7.22.

*4-Bromo-2-((4-(2-(piperidin-1-yl)ethoxy)benzylidene)amino)phenol* **19**. Compound **19** was synthesized according to general procedure Section 3.1.2 from **3** (100 mg, 0.43 mmol), ZnO_NP_ (10 mg, 0.12 mmol) and 2-amino-4-bromophenol (81 mg, 0.43 mmol). Compound **19** was obtained as a dark brown oil. (Method A: 152 mg, 87%; Method B: 149 mg, 86%; Method C: 142 mg, 82%; Method D: 150 mg, 87%; Method E: 104 mg, 60%). ^1^H-NMR (600 MHz, DMSO-*d*_6_) δ 9.25 (s, 1H, OH), 8.66 (s, 1H, N=CH-7), 8.02 (d, *J* = 7.1 Hz, 2H, H-9,13), 7.71 (d, *J* = 7.5 Hz, 1H, H-5), 7.40 (d, *J* = 7.1 Hz, 2H, H-10,12), 7.33 (d, *J* = 2.5 Hz, 1H, H-3), 6.85 (d, *J* = 8.6 Hz, 1H, H-6), 4.24 (t, *J* = 5.9 Hz, 2H, H-1′), 2.71 (m, 2H, H-2′), 2.46 (m, 4H, H-3′,3″), 1.50 (q, *J* = 7.0 Hz, 4H, H-4′,4″), 1.38 (p, *J* = 6.8 Hz, 2H, H-5′). Calcd for C_20_H_23_BrN_2_O_2_: C, 59.56; H, 5.75; N, 6.95; Found: C, 59.57; H, 5.76; N, 6.94.

*4-Bromo-2-((4-(2-morpholinoethoxy)benzylidene)amino)phenol* **20**. Compound **20** was synthesized according to general procedure Section 3.1.2 from **4** (100 mg, 0.42 mmol), ZnO_NP_ (10 mg, 0.12 mmol) and 2-amino-4-bromophenol (80 mg, 0.42 mmol). Compound **21** was obtained as a dark brown oil. (Method A: 140 mg, 81%; Method B: 144 mg, 84%; Method C: 130 mg, 85%; Method D: 139 mg, 91%; Method E: 90 mg, 59%). ^1^H-NMR (600 MHz, DMSO-*d*_6_) δ 9.24 (s, 1H, OH), 8.67 (s, 1H, N=CH-7), 8.03 (d, *J* = 7.6 Hz, 2H, H-9,13), 7.73 (d, *J* = 7.8 Hz, 1H, H-5), 7.30 (d, *J* = 7.6 Hz, 2H, H-10,12), 7.12 (d, *J* = 2.5 Hz, 1H, H-3), 6.90 (d, *J* = 8.1 Hz, 1H, H-6), 4.26 (t, *J* = 5.7 Hz, 2H, H-1′), 3.59 (t, *J* = 4.7 Hz, 4H, H-4′,4″), 2.75 (t, *J* = 5.7 Hz, 2H, H-2′), 2.49 (m, 4H, H-3′,3″). ^13^C-NMR/APT (151 MHz, DMSO-*d*_6_) δ 163.3, 161.4, 148.4, 142.4, 129.1, 128.9, 125.0, 124.2, 119.4, 118.0, 115.2, 109.9, 66.3, 65.4, 56.4, 54.0. Calcd for C_19_H_21_BrN_2_O_3_: C, 56.31; H, 5.22; N, 6.91; Found: C, 56.30; H, 5.24; N, 6.92.

*2-((4-(2-(Diethylamino)ethoxy)-3-methoxybenzylidene)amino)phenol* **21**. Compound **21** was synthesized according to general procedure Section 3.1.2 from **5** (100 mg, 0.40 mmol), ZnO_NP_ (10 mg, 0.12 mmol) and 2-aminophenol (43 mg, 0.40 mmol). Compound **21** was obtained as a light brown oil. (Method A: 120 mg, 88%; Method B: 118 mg, 87%; Method C: 112 mg, 82%; Method D: 115 mg, 84%; Method E: 89 mg, 65%). ^1^H-NMR (300 MHz, DMSO-*d*_6_) δ 8.88 (s, 1H, OH), 8.60 (s, 1H, N=CH-7), 7.76 (d, *J* = 1.9 Hz, 1H, H-9), 7.46 (d, *J* = 1.9 Hz, 1H, H-13), 7.20 (d, *J* = 1.7 Hz, 1H, H-5), 7.08 (d, *J* = 8.3 Hz, 1H, H-4), 6.90 (d, *J* = 1.4 Hz, 1H, H-12), 6.88 (d, *J* = 1.4 Hz, 1H, H-3), 6.82 (d, *J* = 1.5 Hz, 1H, H-6), 4.08 (t, *J* = 6.3 Hz, 2H, H-1′), 3.86 (s, 3H, OCH_3_), 2.81 (t, *J* = 6.3 Hz, 2H, H-2′), 2.56 (t, *J* = 7.1 Hz, 4H, H-3′,3″), 0.98 (t, *J* = 7.1 Hz, 6H, H-4′,4″). Calcd for C_20_H_26_N_2_O_3_: C, 70.15; H, 7.65; N, 8.18; Found: C, 70.14; H, 7.64; N, 8.19.

*2-((3-Methoxy-4-(2-(pyrrolidin-1-yl)ethoxy)benzylidene)amino)phenol* **22**. Compound **22** was synthesized according to general procedure Section 3.1.2 from **6** (100 mg, 0.40 mmol), ZnO_NP_ (10 mg, 0.12 mmol) and 2-aminophenol (43 mg, 0.40 mmol). Compound **22** was obtained as a light orange oil. (Method A: 122 mg, 89%; Method B: 121 mg, 89%; Method C: 108 mg, 80%; Method D: 130 mg, 78%; Method E: 93 mg, 56%). ^1^H-NMR (600 MHz, DMSO-*d*_6_) δ 8.88 (s, 1H, OH), 8.61 (s, 1H, N=CH-7), 7.78 (d, *J* = 1.9 Hz, 1H, H-9), 7.46 (d, *J* = 1.9 Hz, 1H, H-13), 7.21 (d, *J* = 1.6 Hz, 1H, H-5), 7.08 (d, *J* = 1.6 Hz, 1H, H-4), 6.90 (d, *J* = 1.4 Hz, 1H, H-12), 6.88 (d, *J* = 1.4 Hz, 1H, H-3), 6.83 (d, *J* = 1.4 Hz, 1H, H-6), 4.16 (t, *J* = 6.2 Hz, 2H, H-1′), 3.84 (s, 3H, OCH_3_), 2.70 (t, *J* = 6.1 Hz, 2H, H-2′), 2.45 (m, 4H, H-3′,3″), 1.51 (p, *J* = 5.6 Hz, 4H,H-4′,4″). ^13^C-NMR (151 MHz, DMSO-*d*_6_) δ 160.5, 153.9, 150.7, 149.3, 138.9, 130.1, 128.7, 126.1, 125.9, 121.3, 118.7, 112.7, 109.1, 67.5, 55.4, 54.1, 54.0, 23.1.

*2-((3-Methoxy-4-(2-(piperidin-1-yl)ethoxy)benzylidene)amino)phenol* **23**. Compound **23** was synthesized according to general procedure Section 3.1.2 from **7** (100 mg, 0.38 mmol), ZnO_NP_ (10 mg, 0.12 mmol) and 2-aminophenol (41 mg, 0.38 mmol). Compound **23** was obtained as a brown oil. (Method A: 135 mg, 82%; Method B: 131 mg, 78%; Method C: 124 mg, 74%; Method D: 135 mg, 80%; Method E: 82 mg, 49%). ^1^H-NMR (600 MHz, DMSO-*d*_6_) δ 8.88 (s, 1H, OH), 8.61 (s, 1H, N=CH-7), 7.78 (d, *J* = 1.9 Hz, 1H, H-9), 7.46 (d, *J* = 1.9 Hz, 1H, H-5), 7.21 (d, *J* = 1.6 Hz, 1H, H-4), 7.05 (d, *J* = 1.6 Hz, 1H, H-12), 6.90 (d, *J* = 1.4 Hz, 1H, H-3), 6.88 (d, *J* = 1.4 Hz, 1H, H-6), 4.16 (t, *J* = 6.2 Hz, 2H, H-1′), 3.84 (s, 1H, OCH_3_), 2.70 (t, *J* = 6.1 Hz, 2H, H-2′), 2.46 (m, 4H, H-3′,3″), 1.51 (p, *J* = 5.6 Hz, 4H, H-4′,4″), 1.39 (p, *J* = 6.3 Hz, 2H, H-5′). ^13^C-NMR (151 MHz, DMSO-*d*_6_) δ 160.5, 153.9, 150.7, 145.3, 137.4, 130.1, 128.7, 126.1, 125.9, 121.27, 118.69, 112.74, 109.05, 66.7, 59.40, 56.85, 54.3, 53.5, 23.4. Calcd for C_21_H_26_N_2_O_3_: C, 71.16; H, 7.39; N, 7.90; Found: C, 71.15; H, 7.38; N, 7.92.

*2-((3-Methoxy-4-(2-morpholinoethoxy)benzylidene)amino)phenol* **24**. Compound **24** was synthesized according to general procedure Section 3.1.2 from **8** (100 mg, 0.38 mmol), ZnO_NP_ (10 mg, 0.12 mmol) and 2-aminophenol (41 mg, 0.38 mmol). Compound **24** was obtained as a dark brown oil. (Method A: 121 mg, 90%; Method B: 113 mg, 84%; Method C: 106 mg, 79%; Method D: 120 mg, 89%; Method E: 74 mg, 55%). ^1^H-NMR (600 MHz, DMSO-*d*_6_) δ 8.90 (s, 1H, OH), 8.60 (s, 1H, N=CH-7), 7.75 (d, *J* = 1.9 Hz, 1H, H-9), 7.44 (d, *J* = 1.9 Hz, 1H, H-13), 7.18 (d, *J* = 1.6 Hz, 1H, H-5), 7.10 (d, *J* = 8.3 Hz, 1H, H-4), 7.04 (d, *J* = 1.5 Hz, 1H, H-12), 6.89 (d, *J* = 1.4 Hz, 1H, H-3), 6.88 (d, *J* = 1.4 Hz, 1H, H-6), 4.16 (t, *J* = 5.9 Hz, 2H, H-1′), 3.86 (s, 3H, OCH_3_), 3.58 (m, 4H, H-4′,4″), 2.72 (t, *J* = 5.9 Hz, 2H, H-2′), 2.49 (m, 4H, H-3′,3″). Calcd for C_20_H_24_N_2_O_4_: C, 67.40; H, 6.79; N, 7.86; Found: C, 67.41; H, 6.77; N, 7.85.

*4-Chloro-2-((4-(2-(diethylamino)ethoxy)-3-methoxybenzylidene)amino)phenol* **25**. Compound **25** was synthesized according to general procedure Section 3.1.2 from **5** (100 mg, 0.40 mmol), ZnO_NP_ (10 mg, 0.12 mmol) and 2-amino-4-chlorophenol (57 mg, 0.40 mmol). Compound **25** was obtained as a dark yellow oil. (Method A: 120 mg, 82%; Method B: 118 mg, 87%; Method C: 112 mg, 82%; Method D: 115 mg, 84%; Method E: 89 mg, 65%). ^1^H-NMR (600 MHz, DMSO-*d*_6_) δ 8.90 (s, 1H, OH), 8.60 (s, 1H, N=CH-7), 7.75 (d, *J* = 1.9 Hz, 1H, H-9), 7.44 (d, *J* = 1.9 Hz, 1H, H-13), 7.18 (d, *J* = 1.6 Hz, 1H, H-5), 7.06 (d, *J* = 1.6 Hz, 1H, H-3), 6.83 (d, *J* = 1.4 Hz, 1H, H-12), 6.81 (d, *J* = 1.4 Hz, 1H,H-6), 4.16 (t, *J* = 5.9 Hz, 2H, H-1′), 3.86 (s, 3H, OCH_3_), 2.72 (t, *J* = 5.9 Hz, 2H, H-2′), 2.49 (m, 4H, H-3′,3″), 1.06 (t, *J* = 7.0 Hz, 6H, H-4′,4″). ^13^C-NMR (151 MHz, DMSO-*d*_6_) δ 161.6, 153.7, 151.8, 149.4, 138.6, 130.6, 129.0, 126.2, 125.7, 123.5, 119.3, 111.2, 110.5, 67.4, 55.3, 51.2, 46.4, 11.7. Calcd for C_20_H_25_ClN_2_O_3_: C, 63.74; H, 6.69; N, 7.43; Found: C, 63.75; H, 6.68; N, 7.41.

*4-Chloro-2-((3-methoxy-4-(2-(pyrrolidin-1-yl)ethoxy)benzylidene)amino)phenol* **26**. Compound **26** was synthesized according to general procedure Section 3.1.2 from **6** (100 mg, 0.40 mmol), ZnO_NP_ (10 mg, 0.12 mmol) and 2-amino-4-chlorophenol (56 mg, 0.40 mmol). Compound **26** was obtained as a brown oil. (Method A: 125 mg, 83%; Method B: 131 mg, 78%; Method C: 124 mg, 74%; Method D: 135 mg, 80%; Method E: 82 mg, 49%). ^1^H-NMR (600 MHz, DMSO-*d*_6_) δ 8.99 (s, 1H), 8.60 (s, 1H, N=CH-7), 7.76 (d, *J* = 1.9 Hz, 1H, H-9), 7.45 (d, *J* = 1.9 Hz, 1H, H-13), 7.20 (d, *J* = 1.6 Hz, 1H, H-5), 7.10 (d, *J* = 8.3 Hz, 1H, H-12), 7.05 (d, *J* = 1.6 Hz, 1H, H-3), 6.89 (d, *J* = 8.1 Hz, 1H, H-6), 4.14 (t, *J* = 6.0 Hz, 2H, H-1′), 3.87 (s, 3H, OCH_3_), 2.82 (t, *J* = 6.0 Hz, 2H, H-2′), 2.54 (m, 4H, H-3′,3″), 1.69 (p, *J* = 3.1 Hz, 4H, H-4′,4″). ^13^C-NMR/APT (151 MHz, DMSO-*d*_6_) δ 160.4, 153.6, 150.8, 149.2, 139.4, 130.7, 129.8, 126.6, 123.3, 118.2, 114.5, 112.3, 110.6, 66.2, 57.0, 57.4, 56.6, 21.3. Calcd for C_20_H_23_ClN_2_O_3_: C, 64.08; H, 6.18; N, 7.47; Found: C, 64.09; H, 6.17; N, 7.45.

*4-Chloro-2-((3-methoxy-4-(2-(piperidin-1-yl)ethoxy)benzylidene)amino)phenol* **27**. Compound **27** was synthesized according to general procedure Section 3.1.2 from **7** (100 mg, 0.38 mmol), ZnO_NP_ (10 mg, 0.12 mmol) and 2-amino-4-chlorophenol (55 mg, 0.38 mmol). Compound **27** was obtained as a light brown oil. (Method A: 126 mg, 86%; Method B: 122 mg, 83%; Method C: 125 mg, 85%; Method D: 129 mg, 88%; Method E: 86 mg, 58%). ^1^H-NMR (600 MHz, DMSO-*d*_6_) δ 9.25 (s, 1H, OH), 8.62 (s, 1H, N=CH-7), 7.47 (d, *J* = 1.9 Hz, 1H, H-9), 7.26 (d, *J* = 2.6 Hz, 1H, H-13), 7.12 (d, *J* = 8.3 Hz, 1H, H-5), 7.09 (d, *J* = 2.6 Hz, 1H, H-12), 7.08 (d, *J* = 2.6 Hz, 1H, H-3), 6.90 (d, *J* = 8.6 Hz, 1H, H-6), 4.17 (t, *J* = 6.0 Hz, 2H, H-1′), 3.86 (s, 1H, OCH_3_), 2.76 (m, 2H, H-2′), 2.51 (m, 4H, H-3′,3″), 1.53 (p, *J* = 5.6 Hz, 4H, H-4′,4″), 1.38 (m, 2H, H-5′). ^13^C-NMR/APT (151 MHz, DMSO-*d*_6_) δ 161.0, 159.8 150.5, 140.1, 130.2, 129.6, 127.4, 126.5, 120.0, 119.7, 118.0, 112.4, 66.1, 66.5, 57.2, 57.6, 56.7. Calcd for C_21_H_25_ClN_2_O_3_: C, 64.86; H, 6.48; N, 7.20; Found: C, 64.84; H, 6.47; N, 7.22.

*4-Chloro-2-((3-methoxy-4-(2-morpholinoethoxy)benzylidene)amino)phenol* **28**. Compound **28** was synthesized according to general procedure Section 3.1.2 from **8** (100 mg, 0.37 mmol), ZnO_NP_ (10 mg, 0.12 mmol) and 2-amino-4-chlorophenol (54 mg, 0.37 mmol). Compound **28** was obtained as an orange oil. (Method A: 121 mg, 82%; Method B: 119 mg, 80%; Method C: 101 mg, 69%; Method D: 123 mg, 89%; Method E: 77 mg, 52%). ^1^H-NMR (600 MHz, DMSO-*d*_6_) δ 9.25 (s, 1H, OH), 8.62 (s, 1H, N=CH-7), 7.75 (d, *J* = 1.9 Hz, 1H, H-9), 7.47 (d, *J* = 1.9 Hz, 1H, H-13), 7.27 (d, *J* = 2.5 Hz, 1H, H-5), 7.12 (d, *J* = 8.3 Hz, 1H, H-12), 7.10 (d, *J* = 2.6 Hz, 1H, H-3), 6.90 (d, *J* = 8.6 Hz, 1H, H-6), 4.37 (t, *J* = 5.1 Hz, 2H, H-1′), 3.86 (s, 1H, OCH_3_), 3.59 (d, *J* = 4.7 Hz, 4H, H-4′,4″), 2.73 (dd, *J* = 7.0, 4.5 Hz, 2H, H-2′), 2.49 (m, 4H, H-3′,3″). ^13^C-NMR/APT (151 MHz, DMSO-*d*_6_) δ 160.5, 153.9, 150.7, 149.4, 142.1, 138.9, 130.0, 128.7, 126.1, 121.3, 118.4, 112.6, 109.3, 67.4, 65.4, 57.5, 55.1, 55.0. Calcd for C_21_H_25_ClN_2_O_4_: C, 61.46; H, 5.93; N, 7.17; Found: C, 61.44; H, 5.94; N, 7.19.

#### 3.1.3. General Procedure for the Synthesis of Benzoxazole Derivatives **29**–**48**

Method A: To a solution of Schiff bases **9**–**28** in *N*,*N*-dimethylformamide (DMF) (5 mL) sodium cyanide (NaCN) (5 mg, 0.1 mmol) was added. The reaction mixture was stirred for 7–9 h at room temperature in an open flask. The progress of the reaction was monitored by TLC. After completion of the reaction, the reaction mixture was filtered, and the solvent was evaporated under reduced pressure to obtain crude product which was purified by column chromatography (CH_2_Cl_2_:CH_3_OH = 20:1).

Method B: To a solution of Schiff bases **9**–**28** in DMF (5 mL) NaCN (5 mg, 0.1 mmol) was added and the reaction mixture was stirred in a microwave reactor for 3 h at 600 W and 50 °C. The progress of the reaction was monitored by TLC. After completion, the reaction mixture was filtered and the solvent was evaporated under reduced pressure to obtain a crude product which was purified by column chromatography (CH_2_Cl_2_:CH_3_OH = 20:1).

Method C: To a solution of Schiff bases **9**–**28** in DMF (5 mL) NaCN (5 mg, 0.1 mmol) was added. The reaction mixture was heated in an ultrasonic bath for 2 h at 50 °C. The progress of the reaction was monitored by TLC. After completion, the reaction mixture was filtered off and the solvent was evaporated under reduced pressure to obtain a crude product which was purified by column chromatography (CH_2_Cl_2_:CH_3_OH = 20:1).

Method D: To a solution of Schiff bases **9**–**28** in DMF (240 μL) NaCN (5 mg, 0.1 mmol) was added. The reaction mixture was ball milled for 2 h at 14 Hz at room temperature in a closed reaction system. The progress of the reaction was monitored by TLC. After completion, the reaction mixture was dissolved in CH_3_OH, filtered and the solvent was evaporated under reduced pressure to dryness to obtain a crude product which was purified by column chromatography (CH_2_Cl_2_:CH_3_OH = 50:1).

Method E: DES was prepared by mixing choline chloride (ChCl) and glycerol in a ratio of 1:2 at 40 °C until a clear viscous solution was formed. The Schiff bases **9**–**28** were dissolved in DES (10 mL) and NaCN (5 mg, 0.1 mmol) was added. The reaction mixture was stirred for 3 h at 40 °C. The progress of the reaction was monitored by TLC. After completion, the reaction mixture was extracted with CH_2_Cl_2_: H_2_O. The organic layer was dried over MgSO_4_, then filtered and the solvent was evaporated under reduced pressure to dryness. The obtained crude product was purified by column chromatography (CH_2_Cl_2_:CH_3_OH = 50:1).

*2-(4-(2-Diethylaminoethoxy)phenyl)benzo[d]oxazole* **29**. Compound **29** was synthesized according to general procedure Section 3.1.3 from **9** (50 mg, 0.16 mmol) and NaCN. After purification by column chromatography compound **29** was obtained as a white powder. (Method A: 45 mg, 86%; Method B: 42 mg, 85%; Method C: 48 mg, 96%; Method D: 39 mg, 79%; Method E: 74 mg, 96%, m.p. = 115–117 °C). ^1^H NMR (300 MHz, DMSO-*d*_6_) δ 8.17 (d, *J* = 8.8 Hz, 2H, H-9,13), 7.77 (dt, *J* = 6.7, 2.1 Hz, 2H, H-5,6), 7.39 (m, 2H, H-4,7), 7.21 (d, *J* = 8.9 Hz, 2H, H-10,12), 4.40 (s, 2H, H-1′), 3.05 (s, 2H, H-2′), 2.49 (m, 4H, H-3′,3″), 1.19 (t, *J* = 7.4 Hz, 6H, H-4′,4″). ^13^C-NMR (151 MHz, DMSO-*d*_6_) δ 161.9, 159.1, 150.34, 141.86, 129.41, 124.9, 124.1, 119.7, 119.2, 115.53, 110.4, 67.3, 51.5, 46.01, 9. EI^+^ mode: *m*/*z* = 310.9, [M^+^] (calcd for C_19_H_22_N_2_O_2_ = 310.17), Calcd for C_19_H_22_N_2_O_2_: C, 73.52; H, 7.14; N, 9.03. Found: C, 73.50; H, 7.19; N, 9.05.

*2-(4-(2-(Pyrrolidin-1-yl)ethoxy)phenyl)benzo[d]oxazole* **30**. Compound **30** was synthesized according to general procedure Section 3.1.3 from **10** (50 mg, 0.14 mmol) and NaCN. After purification by column chromatography compound **30** was obtained as a light-yellow powder. (Method A: 40 mg, 80%; Method B: 45 mg, 91%; Method C: 43 mg, 87%; Method D: 41 mg, 83%; Method E: 46 mg, 93%, m.p. = 142–144 °C). ^1^H-NMR (300 MHz, DMSO-*d*_6_) δ 8.15 (d, *J* = 8.9 Hz, 2H, H-9,13), 7.76 (m, 2H, H-5,6), 7.39 (m, 2H, H-4,7), 7.19 (d, *J* = 8.8 Hz, 2H, H-10,12), 4.24 (t, *J* = 5.8 Hz, 2H, H-1′), 2.70 (s, 2H, H-2′), 2.52 (m, 4H, H-3′,3″), 1.75 (m, 4H, H-4′,4″). ^13^C-NMR/APT (151 MHz, DMSO-*d*_6_) δ 163.7, 162.1, 149.7, 143.2, 129.7, 126.2, 125.9, 121.2, 118.2, 116.6, 111.4, 67.1, 54.8, 54.2, 22.3. Calcd for C_19_H_20_N_2_O_2_: C, 74.00; H, 6.54; N, 9.08. Found: C, 73.98; H, 6.53; N, 9.06.

*2-(4-(2-(Piperidin-1-yl)ethoxy)phenyl)benzo[d]oxazole* **31**. Compound **31** was synthesized according to general procedure Section 3.1.3 from **11** (50 mg, 0.15 mmol) and NaCN. After purification by column chromatography compound **31** was obtained as a white powder. (Method A: 43 mg, 86%; Method B: 45 mg, 90%; Method C: 41 mg, 83%; Method D: 36 mg, 73%; Method E: 38 mg, 77%, m.p. = 137–139 °C). ^1^H-NMR (300 MHz, DMSO-*d*_6_) δ 8.13 (d, *J* = 8.8 Hz, 2H, H-9,13), 7.76 (m, 2H, H-5,6), 7.39 (m, 2H, H-4,7), 7.16 (d, *J* = 8.8 Hz, 2H, H-10,12), 4.18 (t, *J* = 5.9 Hz, 2H, H-1′), 2.69 (t, *J* = 5.8 Hz, 2H, H-2′), 2.45 (t, *J* = 5.3 Hz, 4H, H-3′,3″), 1.50 (p, *J* = 5.5 Hz, 2H, H-4′,4″), 1.38 (m, 2H, H-5′). ^13^C-NMR/APT (151 MHz, DMSO-*d*_6_) δ 161.8, 160.3, 149.6, 143.9, 129.8, 125.0, 124.7, 121.3, 119.5, 114.1, 113.2, 110.3, 65.2, 57.2, 54.0, 25.4, 24.0. Calcd for C_20_H_22_N_2_O_2_: C, 74.51; H, 6.88; N, 8.69. Found: C, 74.49; H, 6.87; N, 8.71.

*2-(4-(2-Morpholinoethoxy)phenyl)benzo[d]oxazole* **32**. Compound **32** was synthesized according to general procedure Section 3.1.3 from **12** (50 mg, 0.15 mmol) and NaCN. After purification by column chromatography compound **32** was obtained as a white powder. (Method A: 42 mg, 84%; Method B: 44 mg, 89%; Method C: 41 mg, 82%; Method D: 38 mg, 80%; Method E: 42 mg, 85%, m.p. = 156–158 °C). ^1^H-NMR (600 MHz, DMSO-*d*_6_) δ 8.13 (d, *J* = 8.8 Hz, 2H, H-9,13), 7.75 (m, 2H, H-5,6), 7.39 (m, 2H, H-4,7), 7.16 (d, *J* = 8.8 Hz, 2H, H-10,12), 4.20 (t, *J* = 5.7 Hz, 2H, H-1′), 3.58 (t, *J* = 4.7 Hz, 4H, H-4′,4″), 2.72 (t, *J* = 5.7 Hz, 2H, H-2′), 2.48 (m, 4H, H-3′,3″). ^13^C-NMR/APT (151 MHz, DMSO-*d*_6_) δ 162.6, 162.0, 159.6, 150.4, 142.1, 129.2, 125.9, 124.1, 120.4, 119.6, 118.2, 114.4, 110.2, 67.2, 65.2, 47.3, 44.1. EI^+^ mode: *m*/*z* = 324.9, [M^+^] (calcd for C_19_H_20_N_2_O_3_ = 324.15), Calcd for C_19_H_20_N_2_O_3_: C, 70.35; H, 6.21; N, 8.64; Found: C, 70.36; H, 6.23; N, 8.63.

*5-Chloro-2-(4-(2-diethylaminoethoxy)phenyl)benzo[d]oxazole* **33**. Compound **33** was synthesized according to general procedure Section 3.1.3 from **13** (50 mg, 0.14 mmol) and NaCN. After purification by column chromatography compound **33** was obtained as a yellowish powder. (Method A: 39 mg, 80%; Method B: 38 mg, 76%; Method C: 41 mg, 92%; Method D: 43 mg, 87%; Method E: 40 mg, 81%, m.p. = 130–132 °C). ^1^H-NMR (600 MHz, DMSO-*d*_6_) δ 7.86 (d, *J* = 2.1 Hz, 1H, H-4), 7.80 (d, *J* = 8.6 Hz, 2H, H-9,13), 7.67 (d, *J* = 2.0 Hz, 1H, H-7), 7.43 (dd, *J* = 8.6, 2.1 Hz, 1H, H-6), 7.21 (d, *J* = 8.4 Hz, 2H, H-10,12), 4.14 (t, *J* = 6.2 Hz, 2H, H-1′), 2.86 (t, *J* = 6.1 Hz, 2H, H-2′), 2.60 (q, *J* = 7.1 Hz, 4H, H-3′,3″), 1.00 (t, *J* = 7.2 Hz, 6H, H-4′,4″). ^13^C-NMR (151 MHz, DMSO-*d*_6_) δ 162.4, 161.2, 149.4, 143.3, 129.6, 127.7, 126.3, 122.0, 116.7, 113.1, 112.5, 64.2, 54.5, 45.6, 12.8. Calcd for C_19_H_21_ClN_2_O_2_: C, 66.18; H, 6.14; N, 8.12. Found: C, 66.19; H, 6.15; N, 8.10.

*5-Chloro-2-(4-(2-(pyrrolidin-1-yl)ethoxy)phenyl)benzo[d]oxazole* **34**. Compound **34** was synthesized according to general procedure Section 3.1.3 from **14** (50 mg, 0.15 mmol) and NaCN. After purification by column chromatography compound **34** was obtained as a light-yellow powder. (Method A: 41 mg, 81%; Method B: 43 mg, 87%; Method C: 46 mg, 93%; Method D: 40 mg, 81%; Method E: 43 mg, 83%, m.p. = 146–148 °C). ^1^H-NMR (300 MHz, DMSO-*d*_6_) δ 8.12 (d, *J* = 8.9 Hz, 2H, H-9,13), 7.99 (d, *J* = 2.0 Hz, 1H, H-4), 7.74 (d, *J* = 8.6 Hz, 1H, H-7), 7.55 (dd, *J* = 8.6, 2.0 Hz, 1H, H-6), 7.17 (d, *J* = 8.9 Hz, 2H, H-10,12), 4.18 (t, *J* = 5.9 Hz, 2H, H-1′), 2.68 (t, *J* = 5.9 Hz, 2H, H-2′), 2.42 (m, 4H, H-3′,3″), 1.50 (p, *J* = 5.5 Hz, 4H, H-4′,4″). ^13^C-NMR/APT (151 MHz, DMSO-*d*_6_) δ 161.9, 159.9, 153.6, 143.5, 129.8, 125.6, 124.8, 122.0, 121.1, 114.6, 109.3, 66.2, 59.5, 54.3, 26.5. Calcd for C_19_H_19_ClN_2_O_2_: C, 66.57; H, 5.59; N, 8.17;. Found: C, 66.55; H, 5.61; N, 8.15.

*5-Chloro-2-(4-(2-(piperidin-1-yl)ethoxy)phenyl)benzo[d]oxazole* **35**. Compound **35** was synthesized according to general procedure Section 3.1.3 from **15** (50 mg, 0.14 mmol) and NaCN. After purification by column chromatography compound **35** was obtained as a white powder. (Method A: 30 mg, 67%; Method B: 42 mg, 84%; Method C: 39 mg, 78%; Method D: 41 mg, 82%; Method E: 35 mg, 70%, m.p. = 141–143 °C). ^1^H-NMR (600 MHz, DMSO-*d*_6_) δ 7.76 (m, 3H, H-7,9,13), 7.70 (d, *J* = 2.0 Hz, 1H, H-4), 7.40 (d, *J* = 3.7 Hz, 1H, H-6), 7.21 (d, *J* = 8.5 Hz, 2H, H-10,12), 4.18 (t, *J* = 5.9 Hz, 2H, H-1′), 2.74 (t, *J* = 4.7 Hz, 2H, H-2′), 2.48 (m, 4H, H-3′,3″), 1.52 (t, *J* = 5.6 Hz, 4H, H-4′,4″), 1.39 (m, 2H, H-5′). ^13^C-NMR/APT (151 MHz, DMSO-*d*_6_) δ 162.9, 159.7, 149.2, 143.6, 131.6, 128.7, 126.7, 121.9, 116.5, 113.1, 110.2, 64.3, 57.6 54.2, 38.0. Calcd for C_20_H_21_ClN_2_O_2_: C, 67.32; H, 5.93; N, 7.85. Found: C, 67.30; H, 5.94; N, 7.84.

*5-Chloro-2-(4-(2-morpholinoethoxy)phenyl)benzo[d]oxazole* **36**. Compound **36** was synthesized according to general procedure Section 3.1.3 from **16** (50 mg, 0.14 mmol) and NaCN. After purification by column chromatography compound **36** was obtained as a yellowish powder. (Method A: 35 mg, 75%; Method B: 39 mg, 80%; Method C: 37 mg, 80%; Method D: 42 mg, 84%; Method E: 30 mg, 59%, m.p. = 141–143 °C). ^1^H-NMR (300 MHz, DMSO-*d*_6_) δ 8.12 (d, *J* = 8.9 Hz, 2H, H-9,13), 7.85 (d, *J* = 2.2 Hz, 1H, H-4), 7.78 (d, *J* = 8.6 Hz, 1H, H-7), 7.42 (dd, *J* = 8.7, 2.2 Hz, 1H, H-6), 7.17 (d, *J* = 8.9 Hz, 2H, H-10,12), 4.21 (t, *J* = 5.7 Hz, 2H, H-1′), 3.59 (t, *J* = 4.6 Hz, 4H, H-4′,4″), 2.75 (t, *J* = 4.4 Hz, 2H, H-2′), 2.49 (m, 4H, H-3′,3″). ^13^C-NMR/APT (151 MHz, DMSO-*d*_6_) δ 162.0, 159.1, 149.3, 143.1, 129.8, 129.1, 127.7, 121.1, 118.2, 116.6, 100.02, 66.1, 65.8, 56.8, 53.6. Calcd for C_19_H_19_ClN_2_O_3_: C, 63.60; H, 5.34; N, 7.81. Found: C, 63.61; H, 5.32; N, 7.80.

*5-Bromo-2-(4-(2-diethylaminoethoxy)phenyl)benzo[d]oxazole* **37**. Compound **37** was synthesized according to general procedure Section 3.1.3 from **17** (50 mg, 0.14 mmol) and NaCN. After purification by column chromatography compound **37** was obtained as a beige powder. (Method A: 39 mg, 78%; Method B: 37 mg, 74%; Method C: 35 mg, 70%; Method D: 42 mg, 84%; Method E: 44 mg, 87%, m.p. = 139–141 °C). ^1^H-NMR (600 MHz, DMSO-*d*_6_) δ 7.86 (s, 1H, H-4), 7.80 (d, *J* = 8.6 Hz, 2H, H-9,13), 7.77 (dd, *J* = 8.4, 2.0 Hz, 1H, H-7), 7.43 (dd, *J* = 8.6, 2.1 Hz, 1H, H-6), 7.21 (d, *J* = 8.4 Hz, 2H, H-10,12), 4.14 (s, 2H, H-1′), 2.86 (t, *J* = 6.1 Hz, 2H, H-2′), 2.60 (q, *J* = 7.1 Hz, 4H, H-3′,3″), 1.00 (t, *J* = 7.2 Hz, 6H, H-4′,4″). ^13^C-NMR (151 MHz, DMSO-*d*_6_) δ 162.4, 160.1, 151.2, 143.9, 130.2, 127.2, 126.5, 121.3, 118.2, 115.5, 100.4, 67.1, 54.6, 44.2, 13.3. Calcd for C_19_H_21_BrN_2_O_2_: C, 58.62; H, 5.44; N, 7.20. Found: C, 58.62; H, 5.43; N, 7.22.

*5-Bromo-2-(4-(2-(pyrrolidin-1-yl)ethoxy)phenyl)benzo[d]oxazole* **38**. Compound **38** was synthesized according to general procedure Section 3.1.3 from **18** (50 mg, 0.13 mmol) and NaCN. After purification by column chromatography compound **38** was obtained as a beige powder. (Method A: 31 mg, 61%; Method B: 42 mg, 84%; Method C: 39 mg, 78%; Method D: 41 mg, 82%; Method E: 35 mg, 70%, m.p. = 157–159 °C). ^1^H-NMR (600 MHz, DMSO-*d*_6_) δ 8.12 (d, *J* = 8.9 Hz, 2H, H-9,13), 7.99 (d, *J* = 2.0 Hz, 1H, H-4), 7.74 (d, *J* = 8.7 Hz, 1H, H-7), 7.55 (dd, *J* = 8.6, 2.0 Hz, 1H, H-6), 7.17 (d, *J* = 8.9 Hz, 2H, H-10,12), 4.18 (t, *J* = 5.9 Hz, 2H, H-1′), 2.68 (t, *J* = 5.9 Hz, 2H, H-2′), 2.44 (t, *J* = 5.2 Hz, 4H, H-3′,3″), 1.49 (d, *J* = 5.4 Hz, 4H, H-4′,4″). ^13^C-NMR/APT (151 MHz, DMSO-*d*_6_) δ 162.4, 160.1, 151.5, 143.5, 129.8, 126.5, 125.7, 121.7, 120.3, 117.1, 101.3, 67.8, 57.8, 39.8, 23.6. Calcd for C_19_H_19_BrN_2_O_2_: C, 58.93; H, 4.95; N, 7.23. Found: C, 58.95; H, 4.97; N, 7.25.

*5-Bromo-2-(4-(2-(piperidin-1-yl)ethoxy)phenyl)benzo[d]oxazole* **39**. Compound **39** was synthesized according to general procedure Section 3.1.3 from **19** (50 mg, 0.14 mmol) and NaCN. After purification by column chromatography compound **39** was obtained as a yellowish powder. (Method A: 34 mg, 69%; Method B: 38 mg, 76%; Method C: 35 mg, 70%; Method D: 31 mg, 62%; Method E: 36 mg, 72%, m.p. = 155–157 °C). ^1^H-NMR (300 MHz, DMSO-*d*_6_) δ 8.12 (d, *J* = 8.4 Hz, 2H, H-9,13), 8.00 (s, 1H, H-4), 7.75 (d, *J* = 8.5 Hz, 1H, H-7), 7.55 (d, *J* = 8.6 Hz, 1H, H-6), 7.18 (d, *J* = 8.5 Hz, 2H, H-10,12), 4.19 (t, *J* = 5.8 Hz, 2H, H-1′), 2.69 (t, *J* = 5.9 Hz, 2H, H-2′), 2.44 (m, 4H, H-3′,3″), 1.55 (m, 4H, H-4′,4″), 1.39 (m, 1H, H-5′). ^13^C-NMR/APT (151 MHz, DMSO-*d*_6_) δ 163.7, 162.2, 149.2, 143.7, 129.5, 126.2, 125.7, 120.6, 118.2, 116.6, 113.1, 65.1, 57.1, 39.5, 23.9. Calcd for C_20_H_21_BrN_2_O_2_: C, 59.86; H, 5.27; N, 6.98. Found: C, 59.83; H, 5.28; N, 6.96.

*5-Bromo-2-(4-(2-morpholinoethoxy)phenyl)benzo[d]oxazole* **40**. Compound **40** was synthesized according to general procedure Section 3.1.3 from **20** (50 mg, 0.12 mmol) and NaCN. After purification by column chromatography compound **40** was obtained as a light-yellow powder. (Method A: 37 mg, 74%; Method B: 29 mg, 58%; Method C: 31 mg, 62%; Method D: 28 mg, 56%; Method E: 32 mg, 64%, m.p. = 173–175 °C). ^1^H-NMR (300 MHz, DMSO-*d*_6_) δ 7.76 (m, 3H, H-7,9,13), 7.70 (d, *J* = 2.0 Hz, 1H, H-4), 7.39 (m, 1H, H-6), 7.22 (d, *J* = 8.5 Hz, 2H, H-10,12), 4.19 (t, *J* = 5.9 Hz, 2H, H-1′), 3.59 (t, *J* = 4.7 Hz, 4H, H-4′,4″), 2.74 (t, *J* = 5.8 Hz, 2H, H-2′), 2.48 (m, 4H, H-3′,3″). ^13^C-NMR/APT (151 MHz, DMSO-*d*_6_) δ 161.6, 159.1, 149.8, 143.1, 132.4, 130.2, 127.2, 122.0, 118.9, 117.0, 113.2, 112.4, 111.5, 66.3, 54.3, 51.7, 39.5. EI^+^ mode: *m*/*z* = 404.6, [M^+^] (calcd for C_19_H_19_BrN_2_O_3_ = 404.06), Calcd for C_19_H_19_BrN_2_O_3_: C, 56.59; H, 4.75; N, 6.95. Found: C, 56.60; H, 4.74; N, 6.94.

*2-(4-(2-Diethylaminoethoxy)-3-methoxyphenyl)benzo[d]oxazole* **41**. Compound **41** was synthesized according to general procedure Section 3.1.3 from **21** (50 mg, 0.15 mmol) and NaCN. After purification by column chromatography compound **41** was obtained as a yellow powder. (Method A: 41 mg, 82%; Method B: 45 mg, 90%; Method C: 42 mg, 85%; Method D: 48 mg, 48%; Method E: 45 mg, 91%, m.p. = 160–162 °C). ^1^H-NMR (600 MHz, DMSO-*d*_6_) δ 7.78 (m, 3H, H-5,6,13), 7.70 (d, *J* = 2.0 Hz, 1H, H-9), 7.39 (m, 2H, H-4,7), 7.21 (d, *J* = 8.4 Hz, 1H, H-12), 4.13 (t, *J* = 6.2 Hz, 2H, H-1′), 3.90 (s, 3H, OCH_3_), 2.60 (m, 2H, H-2′), 2.51 (m, 2H, H-3′,3″), 1.00 (t, *J* = 7.1 Hz, 6H). ^13^C-NMR/APT (151 MHz, DMSO-*d*_6_) δ 161.7, 152.0, 150.7, 149.7, 143.9, 127.5, 122.2, 121.9, 118.3, 116.4, 113.2, 112.5, 100.9, 67.3, 55.5, 51.0, 47.0, 20.7. Calcd for C_20_H_24_N_2_O_3_: C, 70.57; H, 7.11; N, 8.23. Found: C, C, 70.59; H, 7.13; N, 8.21.

*2-(3-Methoxy-4-(2-(pyrrolidin-1-yl)ethoxy)phenyl)benzo[d]oxazole* **42**. Compound **42** was synthesized according to general procedure Section 3.1.3 from **22** (50 mg, 0.15 mmol) and NaCN. After purification by column chromatography compound **42** was obtained as a white powder. (Method A: 28 mg, 56%; Method B: 27 mg, 54%; Method C: 31 mg, 62%; Method D: 26 mg, 52%; Method E: 24 mg, 48%, m.p. = 167–169 °C). ^1^H-NMR (600 MHz, DMSO-*d*_6_) δ 7.88 (d, *J* = 2.1 Hz, 1H, H-9), 7.81 (d, *J* = 8.6 Hz, 2H, H-5,6), 7.78 (dd, *J* = 8.4, 2.0 Hz, 1H, H-13), 7.68 (d, *J* = 2.0 Hz, 1H, H-7), 7.44 (dd, *J* = 8.6, 2.2 Hz, 1H, H-4), 7.23 (d, *J* = 8.5 Hz, 1H, H-12), 4.18 (t, *J* = 6.0 Hz, 2H, H-1′), 3.90 (s, 1H, OCH_3_), 2.70 (t, *J* = 6.0 Hz, 2H, H-2′), 2.45 (s, 4H, H-3′,3″), 1.50 (p, *J* = 5.6 Hz, 4H, H-4′,4″). ^13^C-NMR/APT (151 MHz, DMSO-*d*_6_) δ 163.1, 151.3, 149.4, 149.3, 141.28, 127.7, 122.0, 122.4, 118.4, 116.7, 113.2, 112.0, 100.1, 67.7, 55.5, 54.1, 54.0, 22.1. Calcd for C_20_H_22_N_2_O_3_: C, 70.99; H, 6.55; N, 8.28;. Found: C, 70.97; H, 6.54; N, 8.29.

*2-(3-Methoxy-4-(2-(piperidin-1-yl)ethoxy)phenyl)benzo[d]oxazole* **43**. Compound **43** was synthesized according to general procedure 3.1.3 from **23** (50 mg, 0.14 mmol) and NaCN. After purification by column chromatography compound **43** was obtained as a white powder. (Method A: 41 mg, 76%; Method B: 35 mg, 58%; Method C: 39 mg, 64%; Method D: 42 mg, 69%; Method E: 47 mg, 72%, m.p. = 167–169 °C). ^1^H-NMR (300 MHz, DMSO-*d*_6_) δ 7.78 (m, 3H, H-5,6,13), 7.41 (m, 2H, H-9,12), 7.26 (d, *J* = 8.5 Hz, 2H, H-4,7), 4.40 (s, 2H, H-1′), 3.92 (s, 3H, OCH_3_), 3.02 (s, 1H, H-2′), 2.48 (m, 4H, H-3′,3″), 1.68 (m, 4H, H-4′,4″), 1.50 (s, 2H, H-5′). ^13^C-NMR/APT (151 MHz, DMSO-*d*_6_) δ 161.8, 152.3, 150.3, 149.5, 141.4, 124.3, 122.1, 117.8, 116.0, 113.5, 111.4, 112.1, 110.7, 67.2, 55.4, 54.2, 53.9. Calcd for C_21_H_24_N_2_O_3_: C, 71.57; H, 6.86; N, 7.95. Found: C, 71.59; H, 6.85; N, 7.97.

*2-(3-Methoxy-4-(2-morpholinoethoxy)phenyl)benzo[d]oxazole* **44**. Compound **44** was synthesized according to general procedure Section 3.1.3 from **24** (50 mg, 0.14 mmol) and NaCN. After purification by column chromatography compound **44** was obtained as a white powder. (Method A: 36 mg, 71%; Method B: 35 mg, 70%; Method C: 31 mg, 62%; Method D: 33 mg, 66%; Method E: 40 mg, 81%, m.p. = 190–192 °C). ^1^H-NMR (300 MHz, DMSO-*d*_6_) δ 7.80 (m, 2H, H-5,6), 7.70 (d, *J* = 2.0 Hz, 1H, H-9), 7.38 (m, 2H, H-12,13), 7.22 (d, *J* = 8.5 Hz, 2H, H-4,7), 4.19 (t, *J* = 5.9 Hz, 2H, H-1′), 3.90 (s, 1H, OCH_3_), 3.59 (t, *J* = 4.7 Hz, 2H, H-4′,4″), 2.74 (t, *J* = 5.8 Hz, 2H, H-2′), 2.48 (m, 2H, H-3′,3″). ^13^C-NMR (151 MHz, DMSO-*d*_6_) δ 161.7, 152.9, 150.3, 149.7, 143.6, 127.6, 126.9, 121.1, 117.6, 116.0, 113.1, 112.2, 111.6, 66.5, 56.2, 54.2, 52.4, 39.5. EI^+^ mode: *m*/*z* = 354.9, [M^+^] (calcd for C_20_H_22_N_2_O_4_ = 354.16), Calcd for C_20_H_22_N_2_O_4_: C, 67.78; H, 6.26; N, 7.80. Found: C, 67.90; H, 6.25; N, 7.91.

*5-Chloro-2-(4-(2-diethylaminoethoxy)-3-methoxyphenyl)benzo[d]oxazole* **45**. Compound **45** was synthesized according to general procedure Section 3.1.3 from **25** (50 mg, 0.15 mmol) and NaCN. After purification by column chromatography compound **45** was obtained as a white powder. (Method A: 40 mg, 80%; Method B: 45 mg, 90%; Method C: 42 mg, 85%; Method D: 48 mg, 48%; Method E: 45 mg, 91%, m.p. = 165–167 °C). ^1^H-NMR (300 MHz, DMSO-*d*_6_) δ 7.83 (m, 1H, H-6), 7.74 (m, 1H, H-13), 7.65 (d, *J* = 2.0 Hz, 1H, H-9), 7.43 (m, 2H, H-4,7), 7.18 (d, *J* = 8.5 Hz, 1H, H-12), 4.10 (t, *J* = 6.2 Hz, 2H, H-1′), 3.89 (s, 1H, OCH_3_), 2.83 (t, *J* = 6.2 Hz, 2H, H-2′), 2.58 (m, 1H, H-3′,3″), 0.99 (t, *J* = 7.1 Hz, 6H, H-4′,4″). ^13^C-NMR (151 MHz, DMSO-*d*_6_) δ 162.6, 152.4, 150.9, 149.5, 143.6, 127.7, 125.1, 121.9, 118.2, 116.6, 113.4, 112.5, 111.2, 67.3, 55.5, 51.7, 46.2, 11.3. Calcd for C_20_H_23_ClN_2_O_3_: C, 64.08; H, 6.18; N, 7.47. Found: C, 64.05; H, 6.20; N, 7.49.

*5-Chloro-2-(3-methoxy-4-(2-(pyrrolidin-1-yl)ethoxy)phenyl)benzo[d]oxazole* **46**. Compound **46** was synthesized according to general procedure Section 3.1.3 from **26** (50 mg, 0.13 mmol) and NaCN. After purification by column chromatography compound **46** was obtained as a beige powder. (Method A: 39 mg, 78%; Method B: 42 mg, 84%; Method C: 38 mg, 76%; Method D: 29 mg, 58%; Method E: 38 mg, 76%, m.p. = 163–165 °C). ^1^H-NMR (300 MHz, DMSO-*d*_6_) δ 7.88 (d, *J* = 2.1 Hz, 1H, H-4), 7.82 (s, 1H, H-9), 7.78 (m, 1H, H-6), 7.68 (d, *J* = 2.0 Hz, 1H, H-7), 7.44 (dd, *J* = 8.6, 2.2 Hz, 2H, H-13,12), 4.18 (t, *J* = 6.0 Hz, 2H, H-2′), 3.90 (s, 1H,OCH_3_), 2.70 (t, *J* = 6.0 Hz, 2H, H-2′), 2.45 (s, 4H, H-3′,3″), 1.50 (p, *J* = 5.6 Hz, 4H, H-4′,4″). ^13^C-NMR/APT (151 MHz, DMSO-*d*_6_) δ 161.2, 152.2, 150.4, 149.7, 143.6, 130.2, 127.6, 123.4, 121.1, 119.5, 113.1, 112.2, 110.6, 108.8, 66.5, 58.1.2, 56.7, 54.4, 39.5. Calcd for C_20_H_21_ClN_2_O_3_: C, 64.43; H, 5.68; N, 7.51;. Found: C, 64.45; H, 5.70; N, 7.49.

*5-Chloro-2-(3-methoxy-4-(2-(piperidin-1-yl)ethoxy)phenyl)benzo[d]oxazole* **47**. Compound **47** was synthesized according to general procedure Section 3.1.3 from **27** (50 mg, 0.12 mmol) and NaCN. After purification by column chromatography compound **47** was obtained as a white powder. (Method A: 34 mg, 69%; Method B: 23 mg, 46%; Method C: 26 mg, 52%; Method D: 30 mg, 60%; Method E: 25 mg, 50%, m.p. = 172–174 °C). ^1^H-NMR (600 MHz, DMSO-*d*_6_) δ 7.88 (d, *J* = 2.1 Hz, 1H, H-4), 7.82 (s, 1H, H-9), 7.78 (dd, *J* = 8.4, 2.0 Hz, 1H, H-13), 7.68 (d, *J* = 2.0 Hz, 1H, H-6), 7.44 (dd, *J* = 8.6, 2.2 Hz, 1H, H-12), 7.23 (d, *J* = 8.5 Hz, 1H, H-7), 4.18 (t, *J* = 6.0 Hz, 2H, H-1′), 3.90 (s, 3H, OCH_3_), 2.70 (t, *J* = 6.0 Hz, 2H, H-2′), 2.45 (m, 4H, H-3′,3″), 1.50 (m, 4H, H-4′,4″), 1.39 (m, 2H, H-5′). ^13^C-NMR/APT (151 MHz, DMSO-*d*_6_) δ 162.1, 152.9, 151.7, 149.1, 143.3, 130.2, 126.4, 121.1, 117.9, 116.5, 114.3, 112.5, 111.8, 67.0, 58.1, 56.2, 55.1, 22.4, 21.3. Calcd for C_21_H_23_ClN_2_O_3_: C, 65.20; H, 5.99; N, 7.24; Found: C, 65.21; H, 6.01; N, 7.22.

*5-Chloro-2-(3-methoxy-4-(2-morpholinoethoxy)phenyl)benzo[d]oxazole* **48**. Compound **48** was synthesized according to general procedure Section 3.1.3 from **28** (50 mg, 0.13 mmol) and NaCN. After purification by column chromatography compound **48** was obtained as a white powder. (Method A: 32 mg, 64%; Method B: 44 mg, 88%; Method C: 41 mg, 82%; Method D: 32 mg, 64%; Method E: 45 mg, 91%, m.p. = 166–168 °C). ^1^H-NMR (600 MHz, DMSO-*d*_6_) δ 7.87 (d, *J* = 2.1 Hz, 1H, H-4), 7.79 (s, 1H, H-9), 7.77 (dd, *J* = 8.4, 2.1 Hz, 1H, H-13), 7.68 (d, *J* = 2.1 Hz, 1H, H-6), 7.44 (dd, *J* = 8.6, 2.2 Hz, 1H, H-12), 7.22 (d, *J* = 8.5 Hz, 1H, H-7), 4.20 (t, *J* = 5.9 Hz, 2H, H-1′), 3.90 (s, 3H, OCH_3_), 3.59 (t, *J* = 4.7 Hz, 4H, H-4′,4″), 2.74 (t, *J* = 5.8 Hz, 2H, H-2′), 2.52 (m, 4H, H-3′,3″). ^13^C-NMR/APT (151 MHz, DMSO-*d*_6_) δ 163.3, 152.3, 151.8, 148.3, 143.7, 129.6, 127.8, 121.6, 118.1, 116.1, 113.1, 112.8, 111.0, 66.4, 56.8, 56.4, 55.8. Calcd for C_20_H_21_ClN_2_O_4_: C, 61.78; H, 5.44; N, 7.20. Found: C, 61.76; H, 5.46; N, 7.23.

### 3.2. Biological Evaluations

#### 3.2.1. In Vitro Antiproliferative Evaluation

For proliferation assays, adherent cell lines LN-229, HCT-116, NCI-H460, and Capan-1 cells were seeded in 384-well tissue culture plates (Greiner, Kremsmünster, Austria) at a density between 500 and 1500 cells per well (500 cells per well for Capan-1, 1000 cells per well for LN-229 and HCT-116, and 1500 cells per well for NCI-H460). The cells were treated with seven different concentrations of the test compounds in a 5-fold dilution series ranging from 100 to 0.006 μM after overnight incubation. Suspension cell lines HL-60, K-562, Z-138, and DND-41 were seeded at densities ranging from 2500 to 5500 cells per well (2500 cells per well for HL-60, K-562, and Z-138, and 5500 cells per well for DND-41) in 384-well culture plates containing the test compounds at the same concentration points. All conditions were incubated for 72 h before measuring the cell viability by the CellTiter 96^®^ Aqueous Non-Radioactive Cell Proliferation Assay (Promega, Madison, WA, USA) according to the manufacturer’s instructions. After 3 h, the absorbance of all conditions was measured at 490 nm using a SpectraMax Plus 384 (Molecular Devices, San Jose, CA, USA), and the OD values were used to calculate the 50% inhibitory concentration (IC50). Compounds were tested in two independent experiments [45].

#### 3.2.2. In Vitro Antibacterial Evaluation

2-arylbenzoxazole derivatives **29**–**48** were evaluated against Gram-negative bacteria *Escherichia coli* (ATCC 25922), *Pseudomonas aeruginosa* (ATCC 9027) and *Klebsiella pneumoniae* (ATCC 27736), as well as Gram-positive bacteria *Staphylococcus aureus* (ATCC 25923) and *Enterococcus faecalis* (ATCC 29212). Activities are expressed as minimum inhibitory concentration (MIC, μg/mL). Standard antibiotics ceftazidime (CAZ) and ciprofloxacin (CIP) were used as reference controls.

Preparation of stock solutions for compounds

The stock solutions of tested compounds were prepared under sterile conditions by dissolving the compound (5.12 mg) in DMSO (1 mL) and homogenization until the sample was completely dissolved. The stock solution had an initial concentration of 5120 μg/mL, from which serial dilutions were prepared to obtain solutions with concentrations of 256 μg/mL, 128 μg/mL, 64 μg/mL, 32 μg/mL, 16 μg/mL, 8 μg/mL, 4 μg/mL, 2 μg/mL, 1 μg/mL, and 0.05 μg/mL.

Preparation of inoculum

Pure bacterial cultures were inoculated onto nutrient agar and incubated at 37 °C the day before setting up the experiment. A suspension of bacteria *Escherichia coli* (ATCC 25922), *Enterococcus faecalis* (ATCC 29212), *Klebsiella pneumoniae* (ATCC 27736), *Pseudomonas aeruginosa* (ATCC 9027) and *Staphylococcus aureus* (ATCC 25923) was prepared into Mueller-Hinton broth (MHB). Pure and separate colonies of the grown bacterial culture were transferred with a sterile microbiological loop to MHB. The concentration of the bacterial suspension that was inoculated into the test tubes was 1 × 10^6^ st/mL, or in the system 5 × 10^5^ st/mL.

Determination of the minimum inhibitory concentration (MIC) using the macrodilution method

The MIC was determined by the macrodilution method according to 03-CLSI-M07-A9-2012 (Methods for Dilution Antimicrobial Susceptibility Tests for Bacteria That Grow Aerobically) [46]. Sterile glass test tubes measuring 13 × 100 mm were used for the experiment. 1 mL of the prepared suspension (1 × 10^6^ st/mL) a certain volume of the tested compound and sterile water were added to each test tube. Inoculum in 1% DMSO were used as positive control, and MHB as a negative control. The total volume of suspension, test compound and sterile water was 2 mL. The test tubes are then homogenized on a homogenizer and incubated at a temperature of 37 °C for 16–20 h. The optical density was observed at 600 nm after 24 h.

## 4. Conclusions

In this study, we describe the sustainable synthesis, antiproliferative and antibacterial evaluation of novel benzoxazole derivatives substituted at position 2 with various 4-*O*-aminoalkylated phenyl substituents and at position 5 of benzoxazole with bromine or chlorine atoms. By applying environmentally friendly synthetic methodologies such as microwave, ultrasound, and mechanochemical reactions, as well as reactions in deep eutectic solvents, *O*-alkylated derivatives of benzaldehyde **1**–**8**, Schiff bases **9**–**28**, and 2-(3,4-disubstituted phenyl)benzoxazole derivatives **29**–**48** were synthesized with high yields in a significantly reduced time compared to conventional synthesis. These methods can be effectively and widely used in the synthesis of other similar derivatives, offering an advantage over conventional synthesis methods for this type of reaction.

The in vitro antiproliferative activity evaluation of the newly synthesised benzoxazole derivatives **29**–**48** against a diverse panel of human cancer cell lines such as LN-229, Capan-1, HCT-116, NCI-H460, DND-41, HL-60, K-562, and Z-138, revealed that the majority of these benzoxazole derivatives displayed promising anticancer activity against the non-small cell lung cancer (NSCLC) cell line NCI-H460. Thus, among all the tested compounds, benzoxazole derivatives **40** and **45** exhibited the most pronounced antiproliferative activity against the NCI-H460 cell lines (IC_50_ = 0.4 and 0.9 µM, respectively). Notably, compounds **30**, **33**, **36**, **39**, **40**, **43** and **45**–**47** showed more pronounced activity (IC_50_ = 0.4–3.8 µM) compared to the reference drug etoposide (IC_50_ = 6.1 µM). Moreover, compounds **36**, **43**, **45** and **46** exhibited promising antiproliferative activities against HCT-116 and LN229 cell lines (IC_50_ = 2.2–14.3 µM). Considering the influence of substituents at positions 3 and 4 of the phenyl ring on antiproliferative activity, it is evident that derivatives bearing a methoxy group **41**–**48** are generally more active than compounds **29**–**40** with an unsubstituted position 3. Furthermore, derivatives **32**, **36**, **40**, **44** and **48** bearing a morpholine substituent show better activity than compounds **29**, **33**, **37** and **41** with an *N*,*N*-diethyl substituent.

The in vitro antibacterial evaluation against Gram-positive and Gram-negative bacteria revealed that most of the tested benzoxazole derivatives lacked antibacterial activity. However, benzoxazole derivative **47** enhanced pronounced activity against the Gram-negative bacterium *P. aeruginosa* (MIC = 0.25 µg/mL) and the Gram-positive bacterium *Enterococcus faecalis* (MIC = 0.5 µg/mL), surpassing the efficacy of the standard antibiotics ceftazidime (CAZ) and ciprofloxacin (CIP).

All presented results point out that this class of benzoxazoles can be effectively prepared using sustainable synthetic methods and present promising candidates for further design and optimization to develop potent antiproliferative agents.

## Figures and Tables

**Table 1:** In vitro antiproliferative activity of novel benzoxazole derivatives **29**–**48**.

IC_50_ (µM)								
Compound	Capan-1	HCT-116	LN229	NCI-H460	DND-41	HL-60	K562	Z138
**29**	74.6 ± 4.9	>100	>100	41.6 ± 3.4	>100	>100	>100	>100
**30**	70.1 ± 1.0	62.0 ± 0.8	33.9 ± 3.0	**1.7** ± 0.2	>100	74.3 ± 4.4	>100	>100
**31**	55.3 ± 7.6	>100	≥59.7	62.8 ± 8.6	≥98.8	≥80.7	>100	≥66.1
**32**	35.2 ± 0.7	36.0 ± 2.4	39.0 ± 2.3	**9.4** ± 0.8	84.3 ± 0.8	44.1 ± 7.3	64.2 ± 5.8	71.3 ± 2.3
**33**	≥96.7	>100	≥81.1	**1.1** ± 0.0	>100	>100	>100	≥56.4
**34**	>100	>100	>100	73.2 ± 0.2	>100	>100	>100	>100
**35**	≥71.5	>100	>100	55.5	>100	>100	>100	≥70.8
**36**	**2.0** ± 0.0	**5.7** ± 1.2	**2.2** ± 0.7	**1.3** ± 0.1	60.4 ± 4.0	52.4 ± 5.8	45.9 ± 1.4	86.2 ± 4.7
**37**	>100	>100	>100	33.1 ± 4.7	>100	>100	>100	≥87.6
**38**	51.7 ± 4.1	>100	>100	30.7 ± 7.0	79.4 ± 1.4	59.0 ± 1.5	>100	56.0 ± 3.1
**39**	≥76.6	>100	>100	**3.8** ± 1.5	>100	>100	>100	>100
**40**	15.7 ± 0.1	>100	>100	**0.4** ± 0.0	77.6 ± 0.6	60.6 ± 4.7	>100	11.2 ± 1.3
**41**	46.2 ± 8.8	16.3 ± 3.0	>100	17.2 ± 0.1	>100	≥51.8	>100	14.4 ± 0.4
**42**	45.8 ± 1.3	>100	>100	14.4 ± 2.8	78.0 ± 1.1	39.9 ± 4.0	>100	>100
**43**	15.4 ± 4.7	**4.7** ± 3.1	**5.6** ± 5.1	**1.8** ± 0.4	>100	53.0 ± 5.1	>100	>100
**44**	74.2 ± 9.5	15.6 ± 0.3	20.3 ± 2.9	16.5 ± 6.0	>100	17.6 ± 5.0	>100	26.4 ± 2.3
**45**	23.6 ± 1.5	**2.4** ± 1.6	14.3 ± 3.0	**0.9** ± 0.1	>100	73.1 ± 4.1	>100	≥53.4
**46**	12.8 ± 2.5	**6.0** ± 5.1	**3.8** ± 2.4	**1.1** ± 0.0	76.4 ± 9.6	24.2 ± 1.6	>100	11.7 ± 3.9
**47**	43.4 ± 9.2	>100	>100	**1.3** ± 0.4	>100	14.8 ± 0.4	>100	16.0 ± 3.9
**48**	≥39.8	>100	≥50.0	15.2 ± 5.2	>100	>100	>100	≥41.9
Eto	0.03 ± 0.0	3.4 ± 0.1	3.7 ± 0.1	6.1 ± 0.4	1.0 ± 0.1	0.8 ± 0.1	4.0 ± 0.6	0.7 ± 0.1
Noco	0.02 ± 0.0	0.04 ± 0.0	0.4 ± 0.3	0.5 ± 0.1	0.7 ± 0.1	0.04 ± 0.0	0.04 ± 0.0	0.04 ± 0.0

Bold: The most pronounced antiproliferative activity.

**Table 2:** In vitro antibacterial activity of novel benzoxazole derivatives **29**–**48**.

MIC (μg/mL)					
Compound	*E. coli*	*P. aeruginosa*	*K. pneumoniae*	*S. aureus*	*E. faecalis*
**29**	>256	>256	>256	>256	**8**
**30, 31**	>256	>256	>256	>256	>256
**32**	256	>256	>256	>256	>256
**33**–**38**	>256	>256	>256	>256	>256
**39**	>256	>256	256	>256	>256
**40**	>256	>256	128	>256	>256
**41**–**46**	>256	>256	>256	>256	>256
**47**	256	**0.25**	>256	>256	**0.5**
**48**	>256	>256	>256	>256	>256
CAZ	0.5	2	256	64	256
CIP	<0.125	0.5	>256	0.5	1

Bold: The most pronounced antibacterial activity.

## Data Availability

The data supporting this article are available in the Supplementary Materials. Additional data may be obtained from the corresponding author upon request (tgazivod@fkit.unizg.hr).

## Supplementary Materials

The following supporting information can be downloaded at: https://www.mdpi.com/article/10.3390/molecules30081767/s1, Supplementary Materials include Figures S1–S89 of ^1^H and ^13^C NMR spectra and Figures S90–S93 of mass spectra.
