# Supplementary material for: A Sustainable Synthesis of Novel 2-(3,4-Disubstituted phenyl)benzoxazole Derivatives and Their Antiproliferative and Antibacterial Evaluation
Source: Molecules. 2025 Apr 15;30(8):1767. doi: 10.3390/molecules30081767 (PMC12029548; doi:10.3390/molecules30081767)
Supplement: Supplementary file 1 [file molecules-30-01767-s001.zip › molecules-3528904-supplementary.pdf]

Supplementary Data

A Sustainable Synthesis of Novel 2-(3,4-Disubstituted phenyl)benzoxazole Derivatives and Their Antiproliferative and Antibacterial Evaluation

Anja Rakas [1], Leentje Persoons [2], Dirk Daelemans [2], Dajana Kučić Grgić [3] and Tatjana Gazivoda Kraljević [1,4,*]

[1] *Department of Organic Chemistry, Faculty of Chemical Engineering and Technology, University of Zagreb, Marulićev Trg 20, 10000 Zagreb*

[2] *Molecular Genetics and Therapeutics in Virology and Oncology Research Group, Department of Microbiology, Immunology and Transplantation, Rega Institute for Medical Research, KU Leuven, 3000 Leuven, Belgium*

[3] *Department of Industrial Ecology, Faculty of Chemical Engineering and Technology, University of Zagreb, Marulićev Trg 19, 10000 Zagreb, Croatia*

[4] *Department for Packaging, Recycling and Environmental Protection, University North, Trg dr. Žarka Dolinara 1, 48000 Koprivnica, Croatia*

Table of Contents

Figure S1. ^{1}H NMR spectrum of compound **1** 5
Figure S2. ^{13}C NMR (APT) spectrum of compound **1** 5
Figure S3. ^{1}H NMR spectrum of compound **2** 6
Figure S4. ^{13}C NMR (APT) spectrum of compound **2** 6
Figure S5. ^{1}H NMR spectrum of compound **3** 7
Figure S6. ^{13}C NMR spectrum of compound **3** 7
Figure S7. ^{1}H NMR spectrum of compound **4** 8
Figure S8. ^{13}C NMR spectrum of compound **4** 8
Figure S9. ^{1}H NMR spectrum of compound **5** 9
Figure S10. ^{13}C NMR (APT) spectrum of compound **5** 9
Figure S11. ^{1}H NMR spectrum of compound **6** 10
Figure S12. ^{13}C NMR (APT) spectrum of compound **6** 10
Figure S13. ^{1}H NMR spectrum of compound **7** 11
Figure S14. ^{13}C NMR (APT) spectrum of compound **7** 11
Figure S15. ^{1}H NMR spectrum of compound **8** 12
Figure S16. ^{13}C NMR (APT) spectrum of compound **8** 12

Figure S17. ^{1}H NMR spectrum of compound **9** 13
Figure S18. ^{13}C NMR (APT) spectrum of compound **9**........ 13
Figure S19. ^{1}H NMR spectrum of compound **10** 14
Figure S20. ^{13}C NMR (APT) spectrum of compound **10**........ 14
Figure S21. ^{1}H NMR spectrum of compound **11** 15
Figure S22. ^{13}C NMR (APT) spectrum of compound **11**........ 15
Figure S23. ^{1}H NMR spectrum of compound **12** 16
Figure S24. ^{13}C NMR (APT) spectrum of compound **12**........ 16
Figure S25. ^{1}H NMR spectrum of compound **13** 17
Figure S26. ^{13}C NMR (APT) spectrum of compound **13**........ 17
Figure S27. ^{1}H NMR spectrum of compound **14** 18
Figure S28. ^{1}H NMR spectrum of compound **15** 18
Figure S29. ^{1}H NMR spectrum of compound **16** 19
Figure S30. ^{13}C NMR (APT) spectrum of compound **16**........ 19
Figure S31. ^{1}H NMR spectrum of compound **17** 20
Figure S32. ^{13}C NMR (APT) spectrum of compound **17**........ 20
Figure S33. ^{1}H NMR spectrum of compound **18** 21
Figure S34. ^{13}C NMR (APT) spectrum of compound **18**........ 21
Figure S35. ^{1}H NMR spectrum of compound **19** 22
Figure S36. ^{1}H NMR spectrum of compound **20** 22
Figure S37. ^{13}C NMR (APT) spectrum of compound **20**........ 23
Figure S38. ^{1}H NMR spectrum of compound **21** 23
Figure S39. ^{1}H NMR spectrum of compound **22** 24
Figure S40. ^{1}H NMR spectrum of compound **23** 24
Figure S41. ^{13}C NMR (APT) spectrum of compound **23**........ 25
Figure S42. ^{1}H NMR spectrum of compound **24** 25
Figure S43. ^{1}H NMR spectrum of compound **25** 26
Figure S44. ^{1}H NMR spectrum of compound **26** 26
Figure S45. ^{13}C NMR (APT) spectrum of compound **26**........ 27
Figure S46. ^{1}H NMR spectrum of compound **27** 27
Figure S47. ^{13}C NMR (APT) spectrum of compound **27**........ 28
Figure S48. ^{1}H NMR spectrum of compound **28** 28
Figure S49. ^{13}C NMR (APT) spectrum of compound **28**........ 29

Figure S50. ^{1}H NMR spectrum of compound **29** 29
Figure S51. ^{13}C NMR spectrum of compound **29** 30
Figure S52. ^{1}H NMR spectrum of compound **30** 30
Figure S53. ^{13}C NMR (APT) spectrum of compound **30**........ 31
Figure S54. ^{1}H NMR spectrum of compound **31** 31
Figure S55. ^{13}C NMR (APT) spectrum of compound **31**........ 32
Figure S56. ^{1}H NMR spectrum of compound **32** 32
Figure S57. ^{13}C NMR (APT) spectrum of compound **32**........ 33
Figure S58. ^{1}H NMR spectrum of compound **33** 33
Figure S59. ^{13}C NMR spectrum of compound **33** 34
Figure S60. ^{1}H NMR spectrum of compound **34** 34
Figure S61. ^{13}C NMR (APT) spectrum of compound **34**........ 35
Figure S62. ^{1}H NMR spectrum of compound **35** 35
Figure S63. ^{13}C NMR (APT) spectrum of compound **35**........ 36
Figure S64. ^{1}H NMR spectrum of compound **36** 36
Figure S65. ^{13}C NMR (APT) spectrum of compound **36**........ 37
Figure S66. ^{1}H NMR spectrum of compound **37** 37
Figure S67. ^{13}C NMR spectrum of compound **37** 38
Figure S68. ^{1}H NMR spectrum of compound **38** 38
Figure S69. ^{13}C NMR (APT) spectrum of compound **38**........ 39
Figure S70. ^{1}H NMR spectrum of compound **39** 39
Figure S71. ^{13}C NMR (APT) spectrum of compound **39**........ 40
Figure S72. ^{1}H NMR spectrum of compound **40** 40
Figure S73. ^{13}C NMR (APT) spectrum of compound **40**........ 41
Figure S74. ^{1}H NMR spectrum of compound **41** 41
Figure S75. ^{13}C NMR (APT) spectrum of compound **41**........ 42
Figure S76. ^{1}H NMR spectrum of compound **42** 42
Figure S77. ^{13}C NMR (APT) spectrum of compound **42**........ 43
Figure S78. ^{1}H NMR spectrum of compound **43** 43
Figure S79. ^{13}C NMR (APT) spectrum of compound **43**........ 44
Figure S80. ^{1}H NMR spectrum of compound **44** 44
Figure S81. ^{13}C NMR spectrum of compound **44** 45
Figure S82. ^{1}H NMR spectrum of compound **45** 45

Figure S83. ^{13}C NMR spectrum of compound **45** 46
Figure S84. ^{1}H NMR spectrum of compound **46** 46
Figure S85. ^{13}C NMR (APT) spectrum of compound **46** 47
Figure S86. ^{1}H NMR spectrum of compound **47** 47
Figure S87. ^{13}C NMR (APT) spectrum of compound **47** 48
Figure S88. ^{1}H NMR spectrum of compound **48** 48
Figure S89. ^{13}C NMR (APT) spectrum of compound **48** 49
Figure S90. Mass spectrum of compund **29** 49
Figure S91. Mass spectrum of compound **32** 50
Figure S92. Mass spectrum of compound **40** 50
Figure S93. Mass spectrum of compound **44** 51

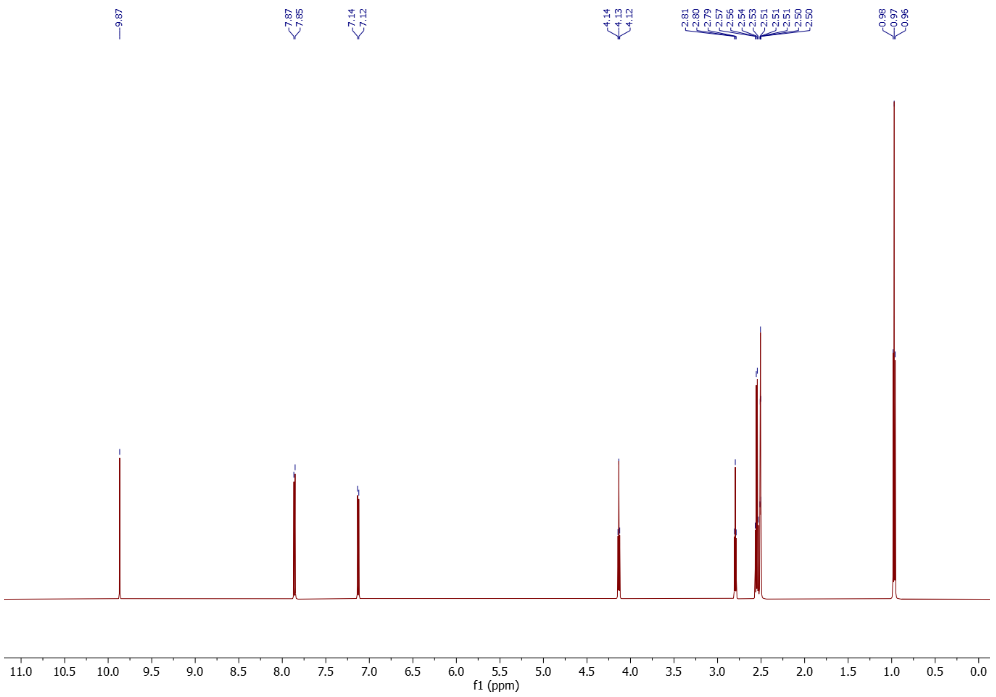

Figure S1. ^{1}H NMR spectrum of compound **1**

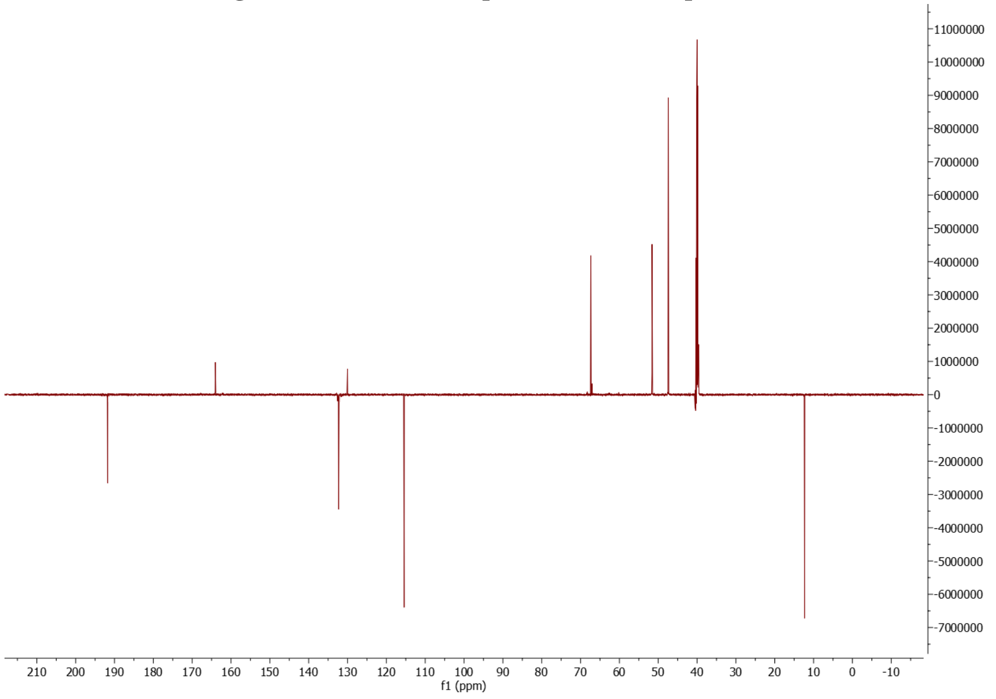

Figure S2. ^{13}C NMR (APT) spectrum of compound **1**

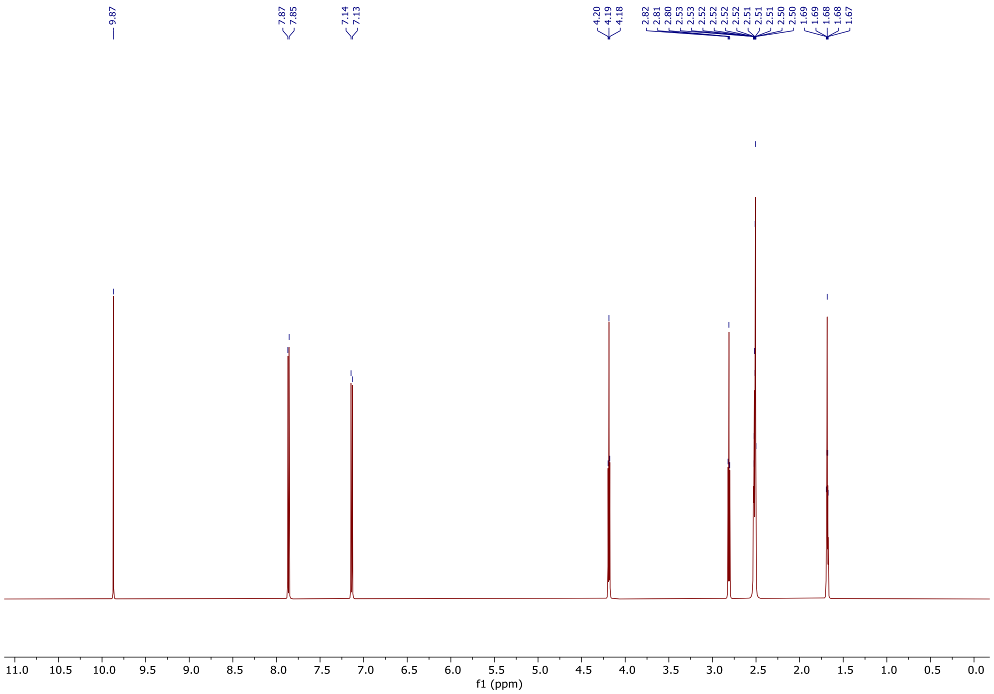

Figure S3. ^{1}H NMR spectrum of compound **2**

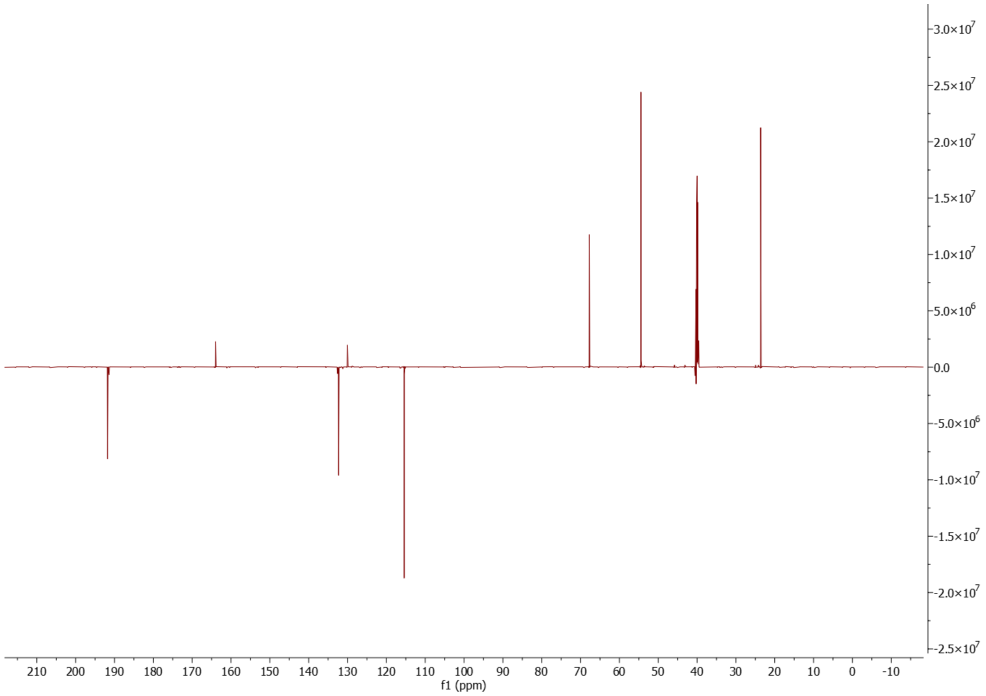

Figure S4. ^{13}C NMR (APT) spectrum of compound **2**

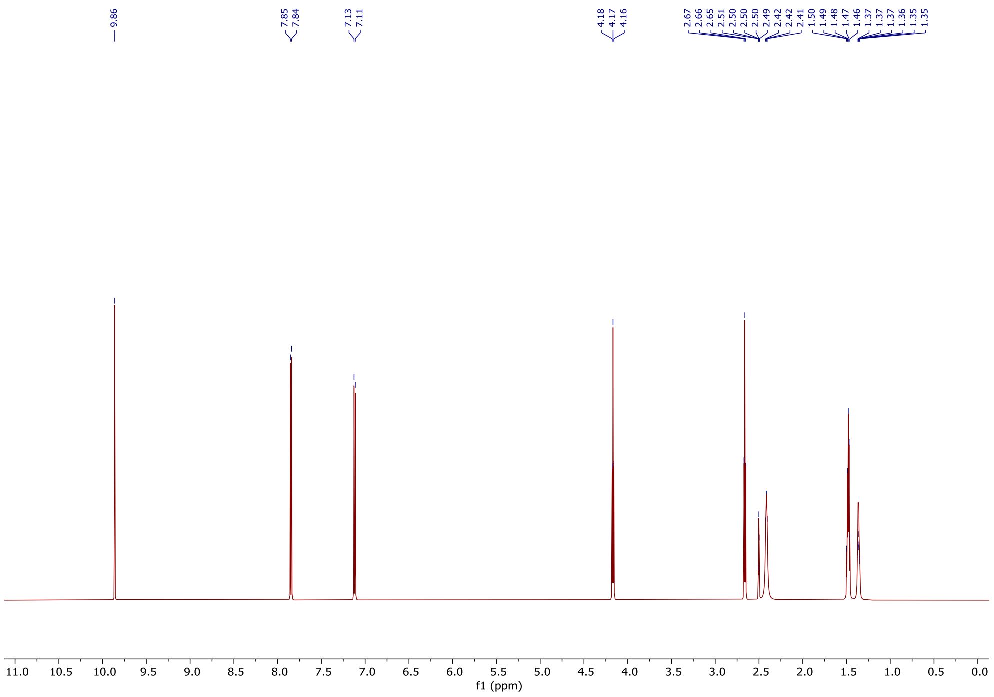

Figure S5. ^{1}H NMR spectrum of compound **3**

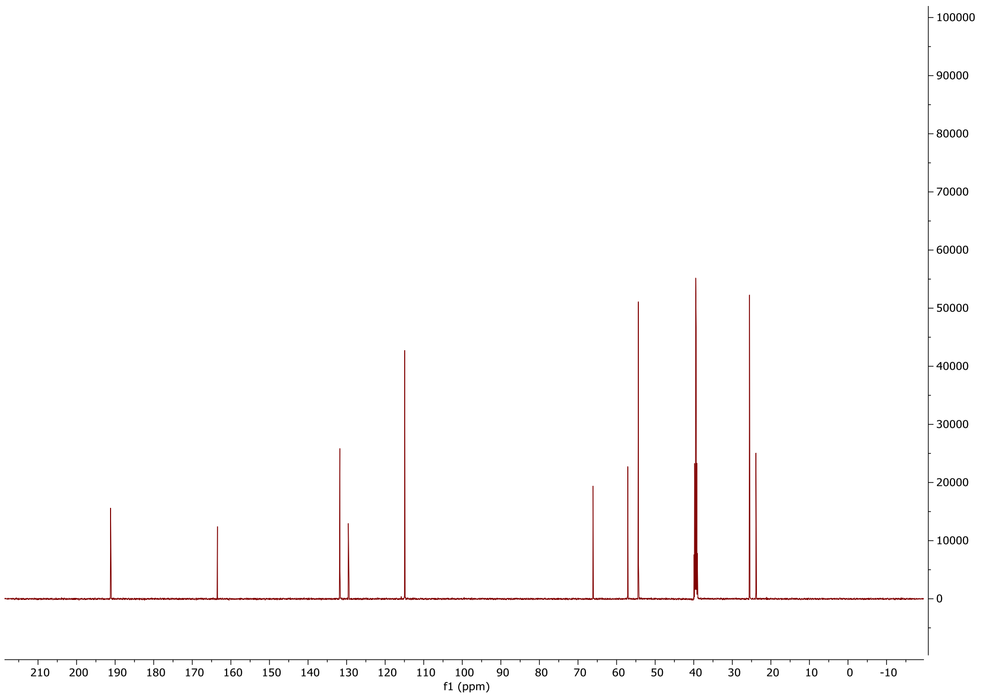

Figure S6. ^{13}C NMR spectrum of compound **3**

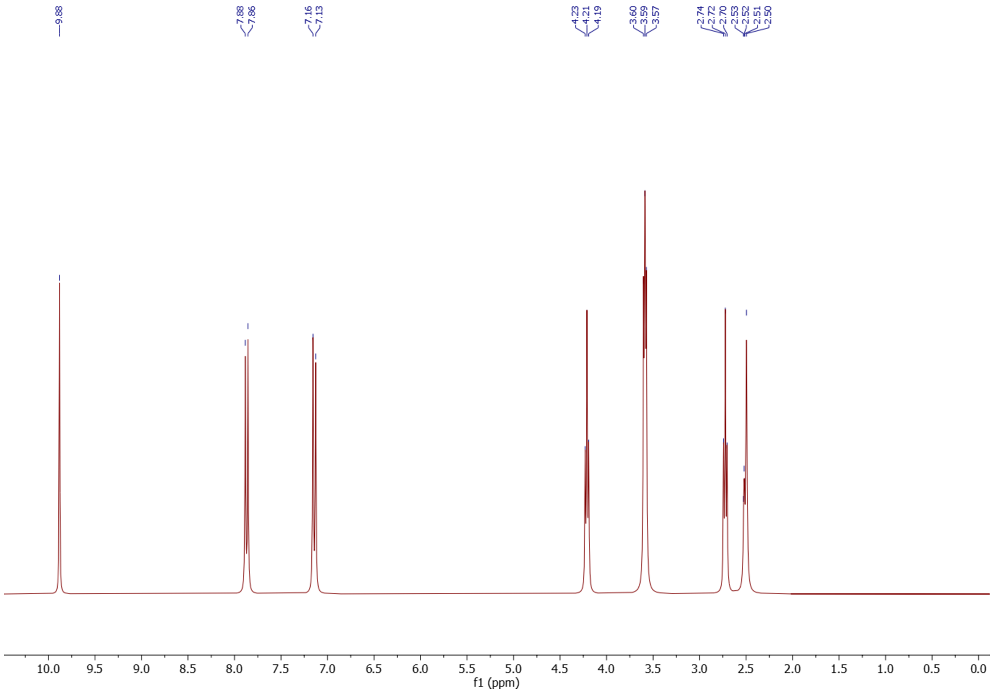

Figure S7. ^{1}H NMR spectrum of compound **4**

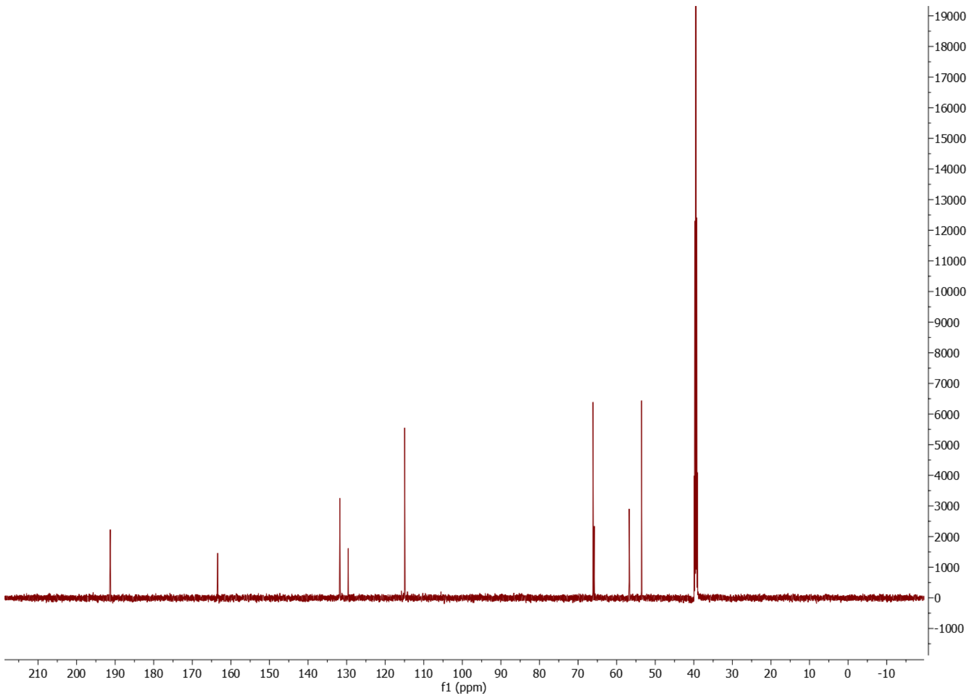

Figure S8. ^{13}C NMR spectrum of compound **4**

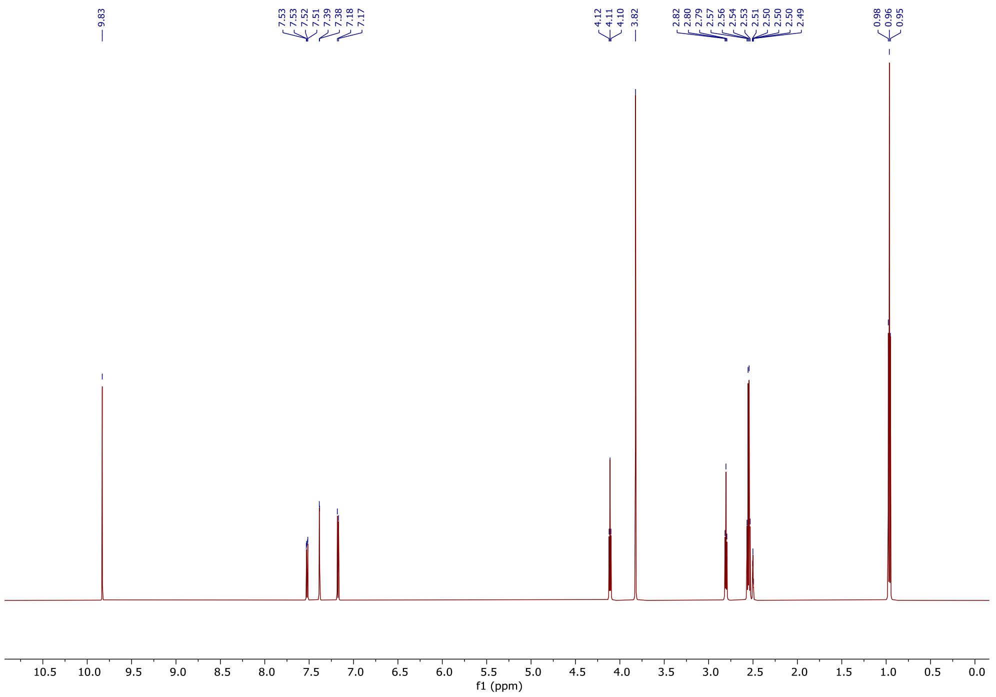

Figure S9. ^{1}H NMR spectrum of compound **5**

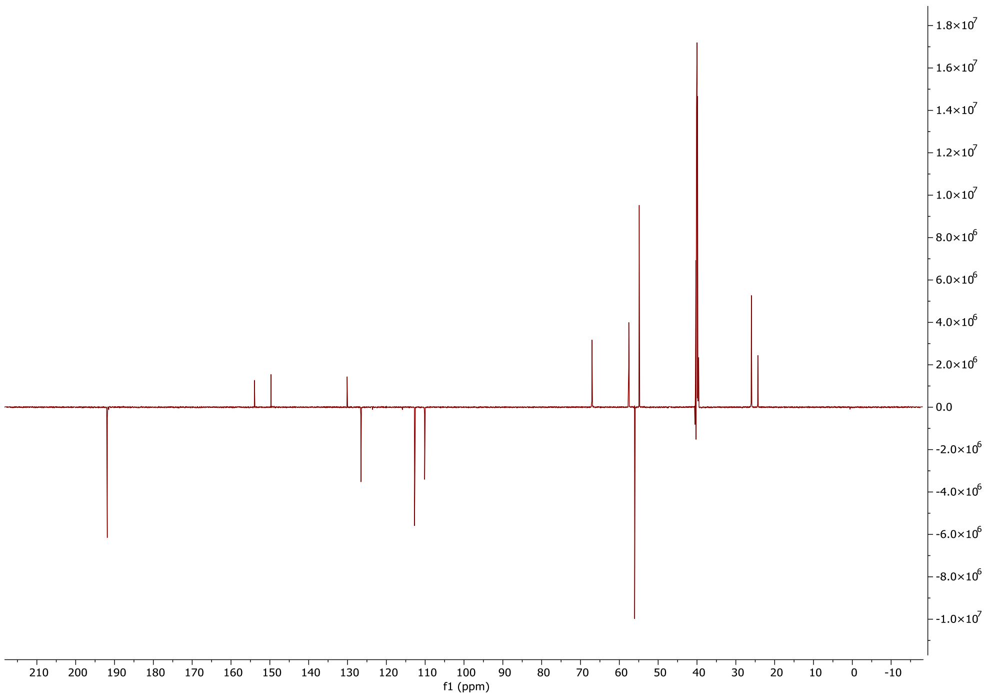

Figure S10. ^{13}C NMR (APT) spectrum of compound **5**

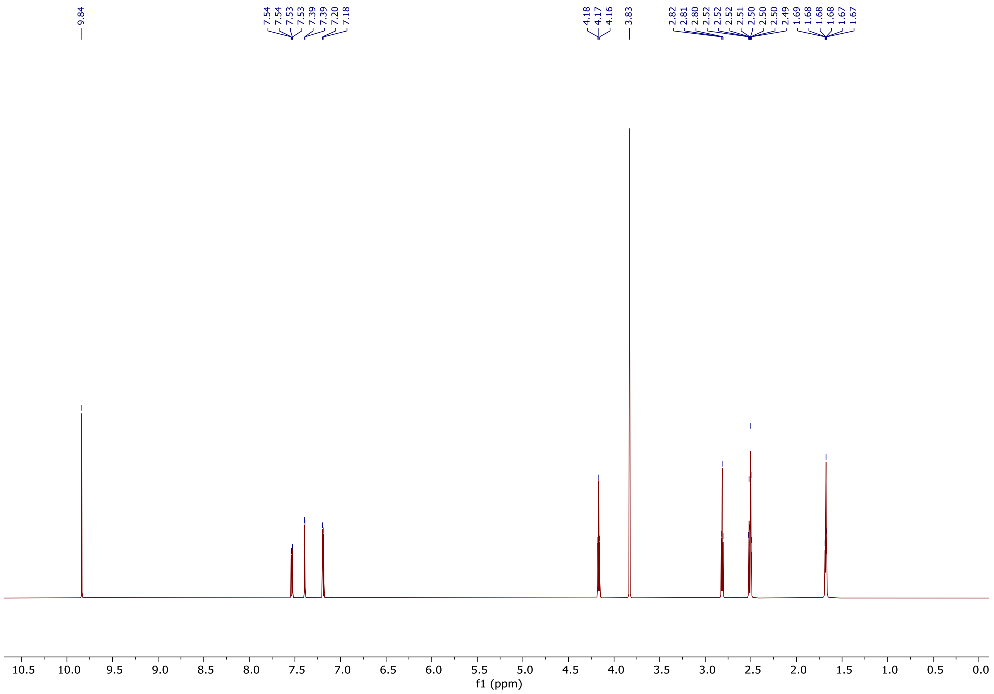

Figure S11. ^{1}H NMR spectrum of compound **6**

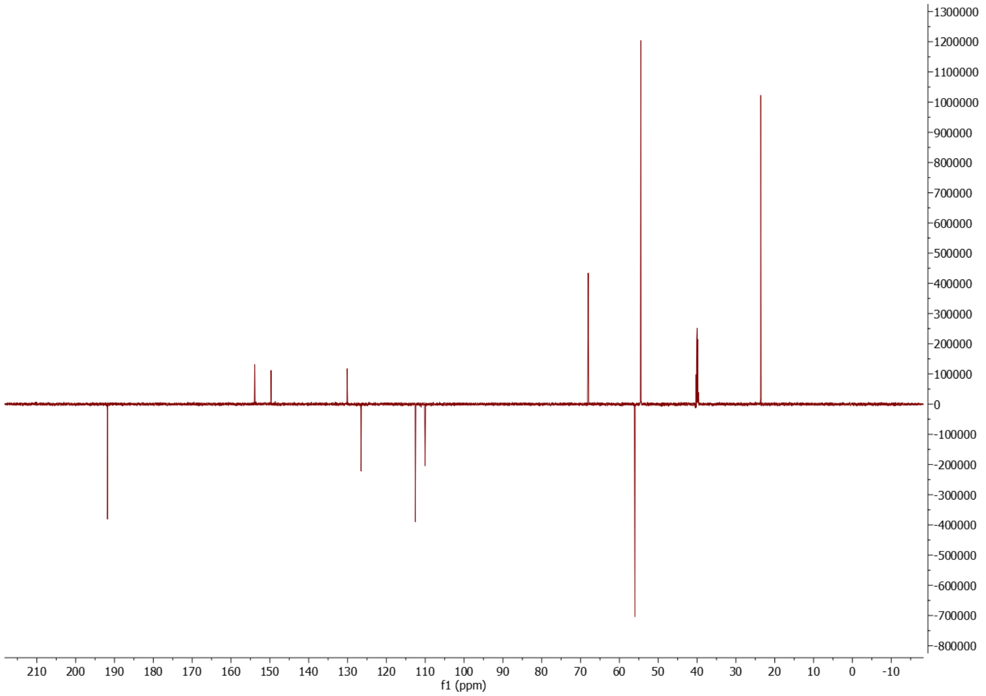

Figure S12. ^{13}C NMR (APT) spectrum of compound **6**

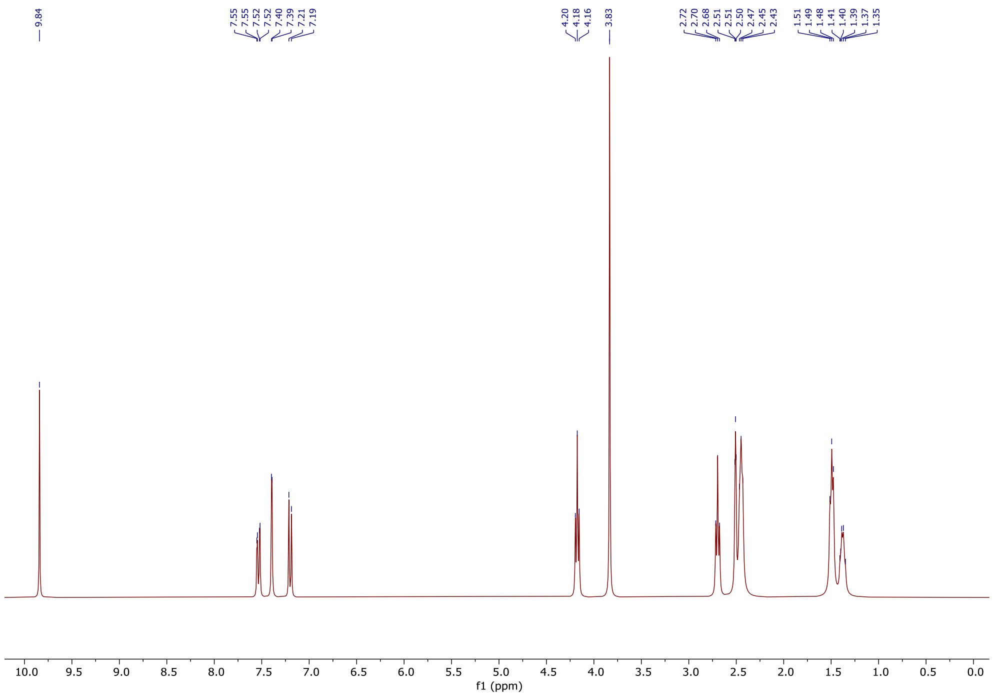

Figure S13. ^{1}H NMR spectrum of compound **7**

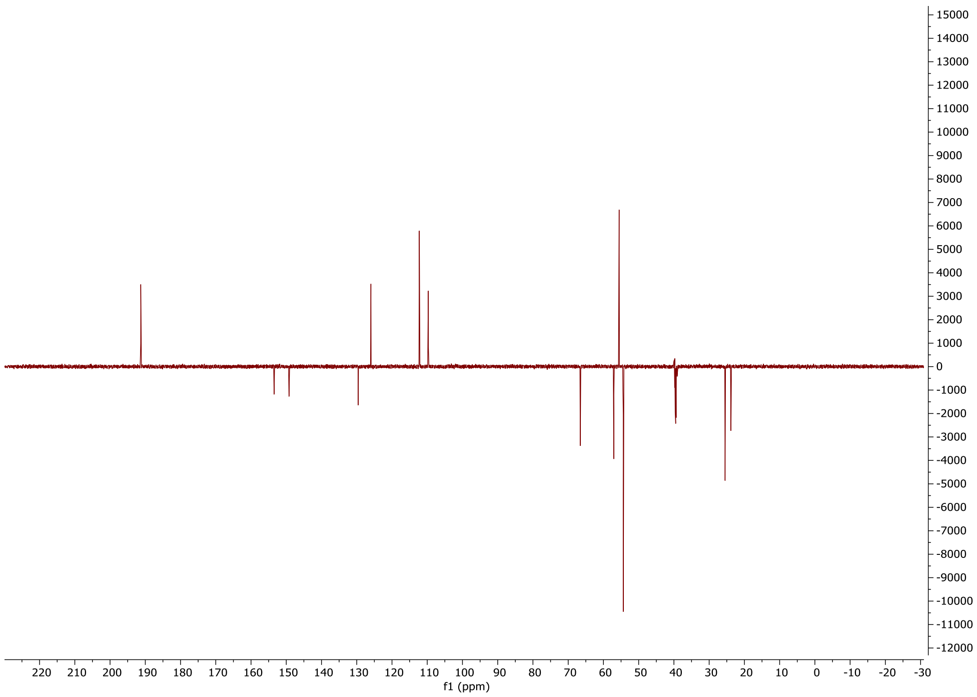

Figure S14. ^{13}C NMR (APT) spectrum of compound **7**

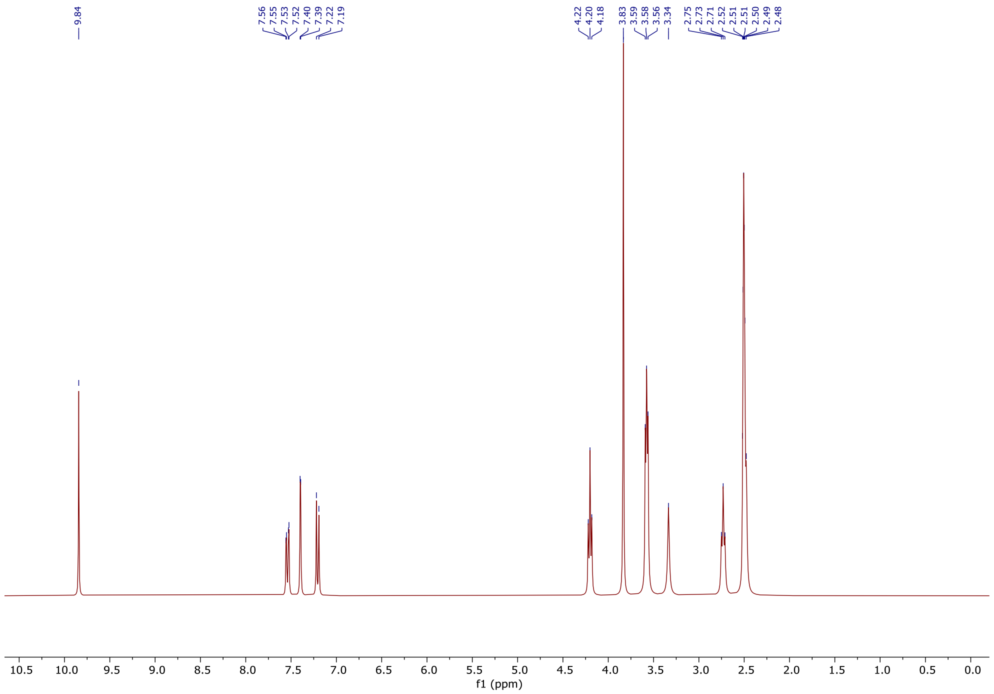

Figure S15. ^{1}H NMR spectrum of compound **8**

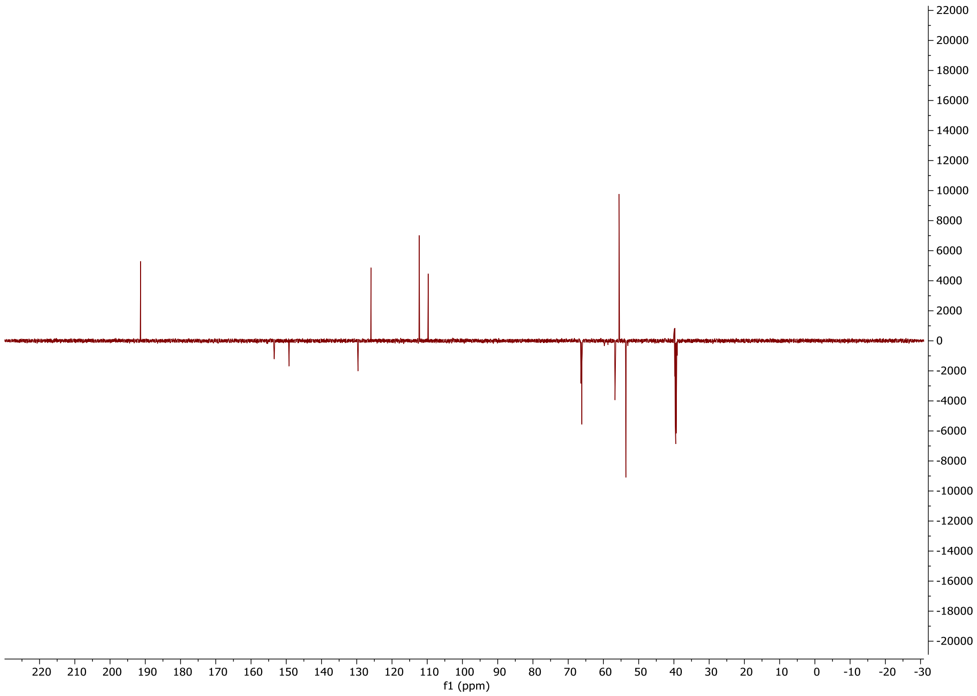

Figure S16. ^{13}C NMR (APT) spectrum of compound **8**

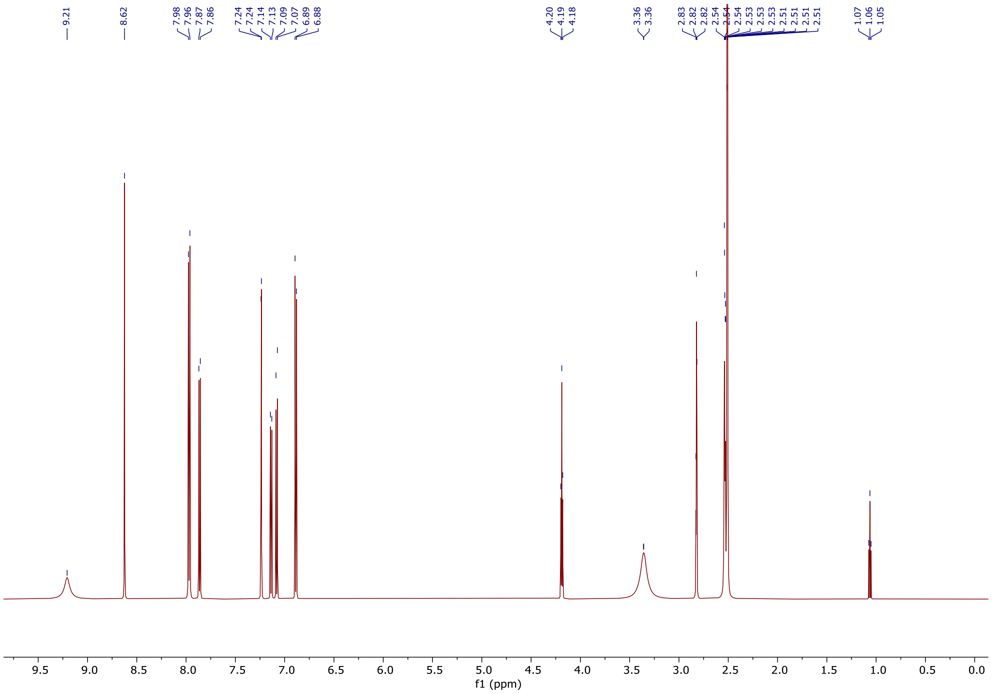

Figure S17. ^{1}H NMR spectrum of compound **9**

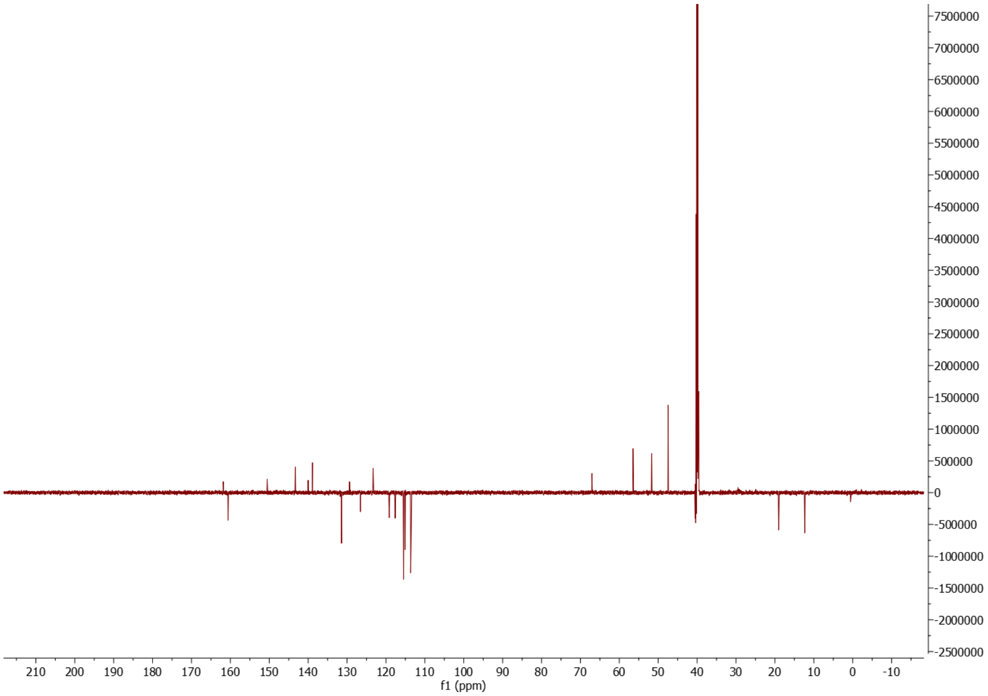

Figure S18. ^{13}C NMR (APT) spectrum of compound **9**

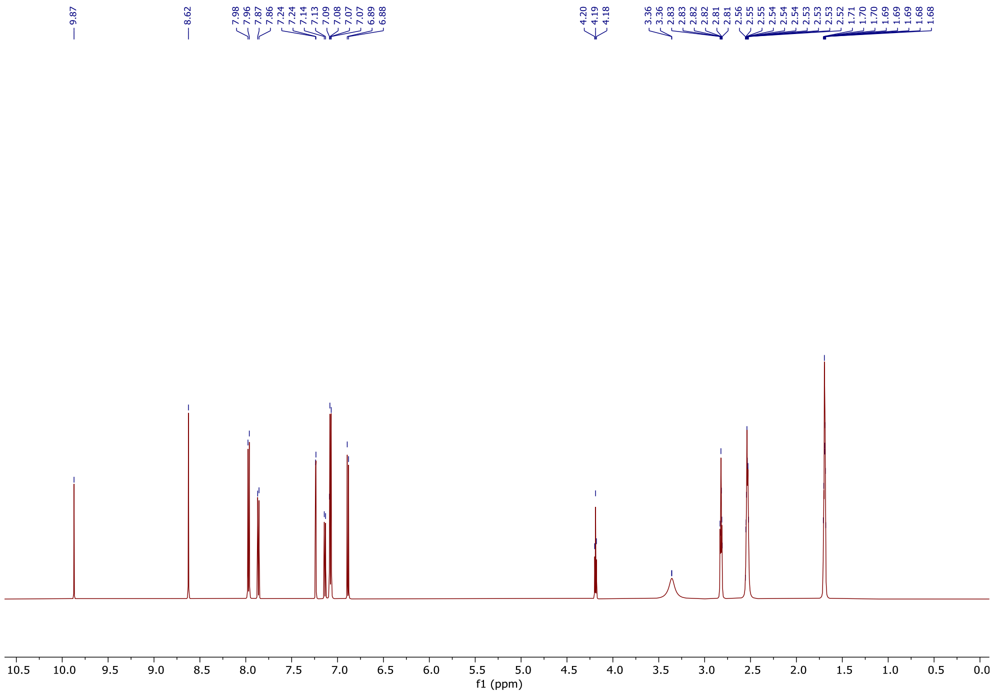

Figure S19. ^{1}H NMR spectrum of compound **10**

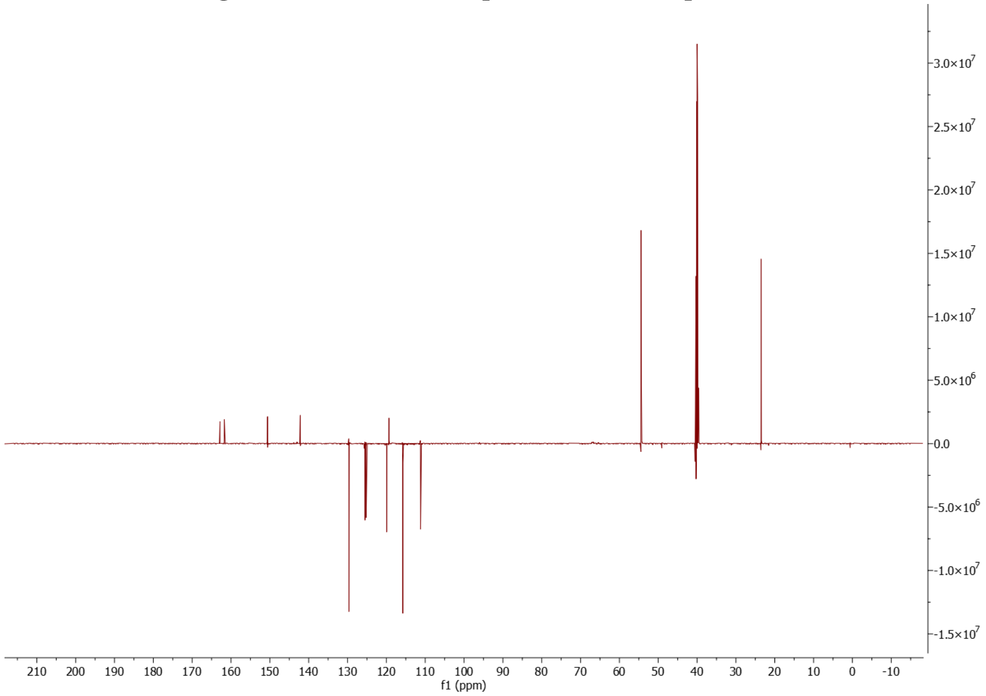

Figure S20. ^{13}C NMR (APT) spectrum of compound **10**

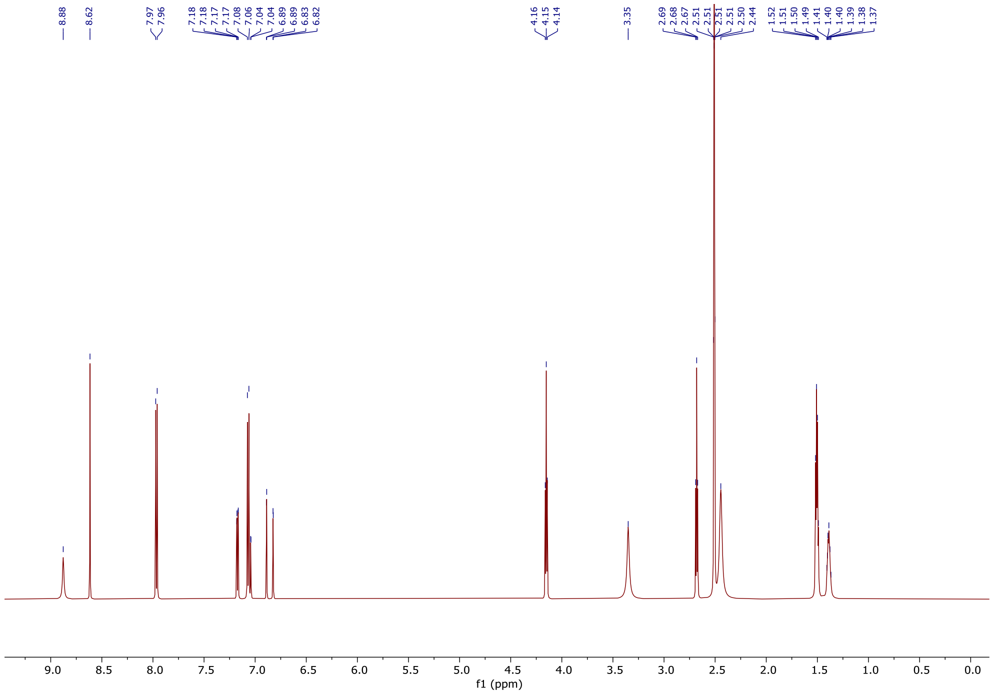

Figure S21. ^{1}H NMR spectrum of compound **11**

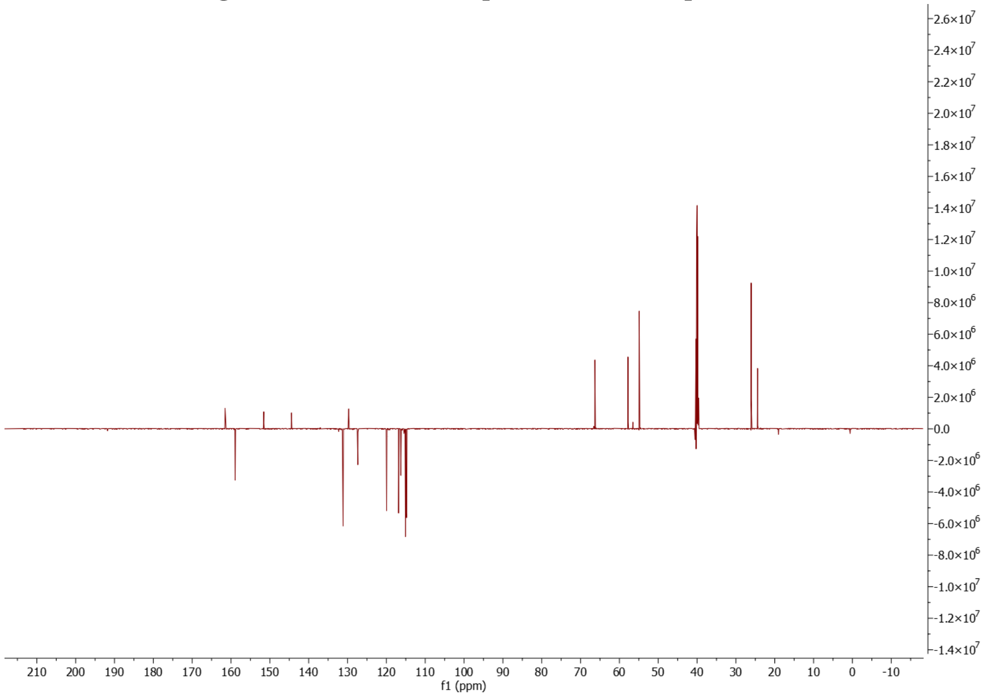

Figure S22. ^{13}C NMR (APT) spectrum of compound **11**

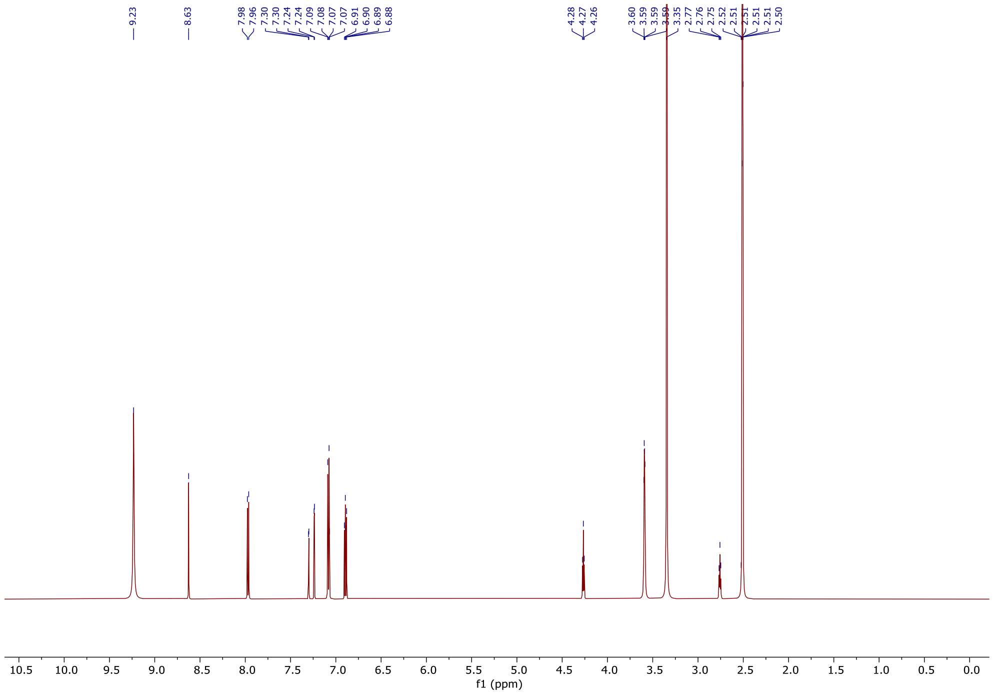

Figure S23. ^{1}H NMR spectrum of compound **12**

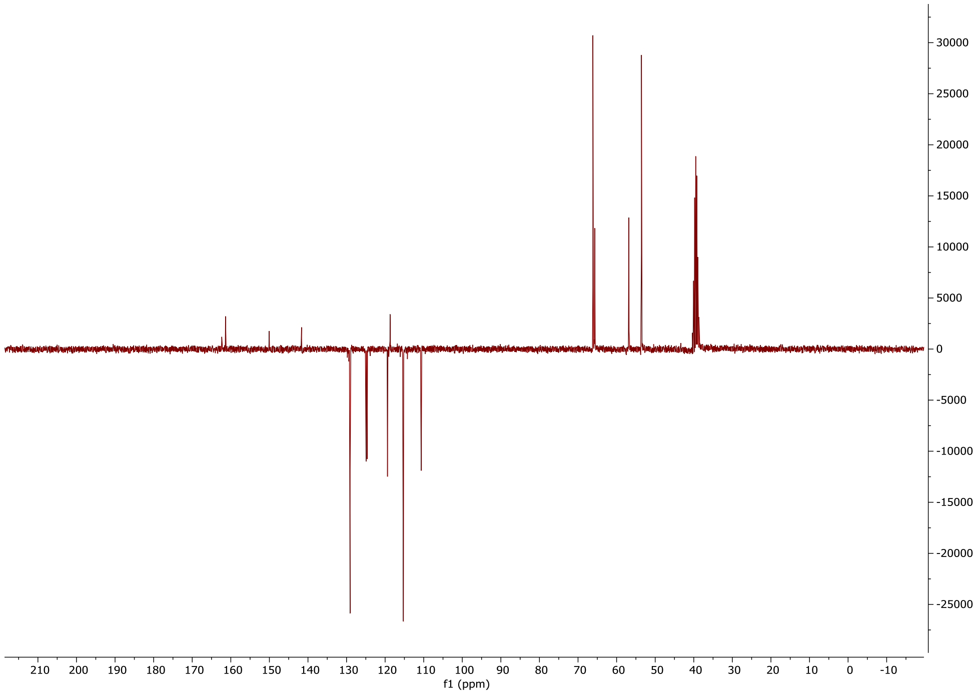

Figure S24. ^{13}C NMR (APT) spectrum of compound **12**

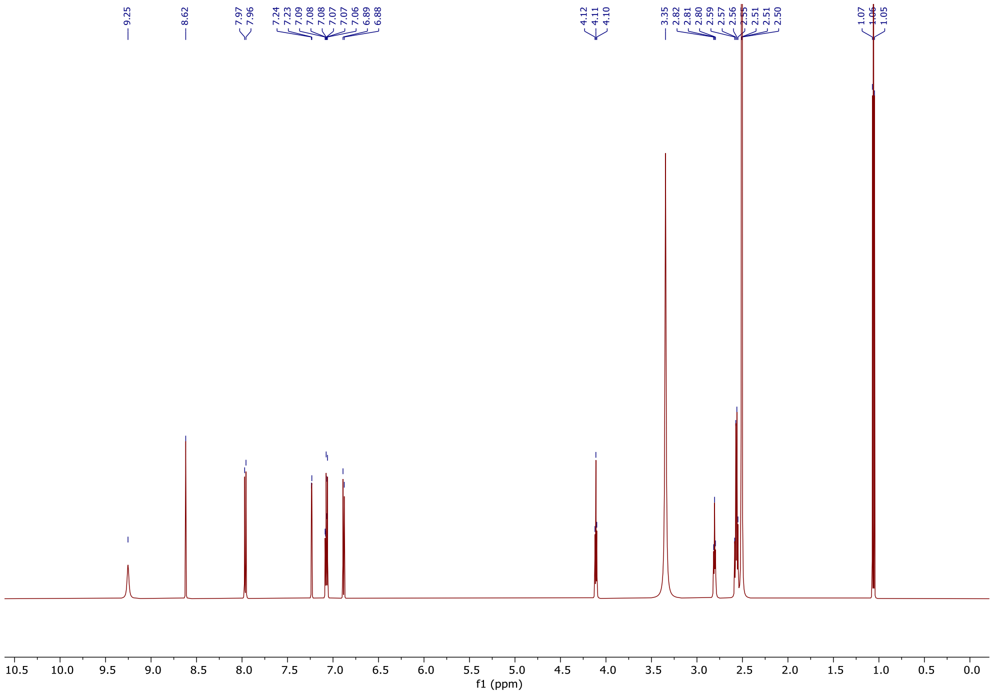

Figure S25. ^{1}H NMR spectrum of compound **13**

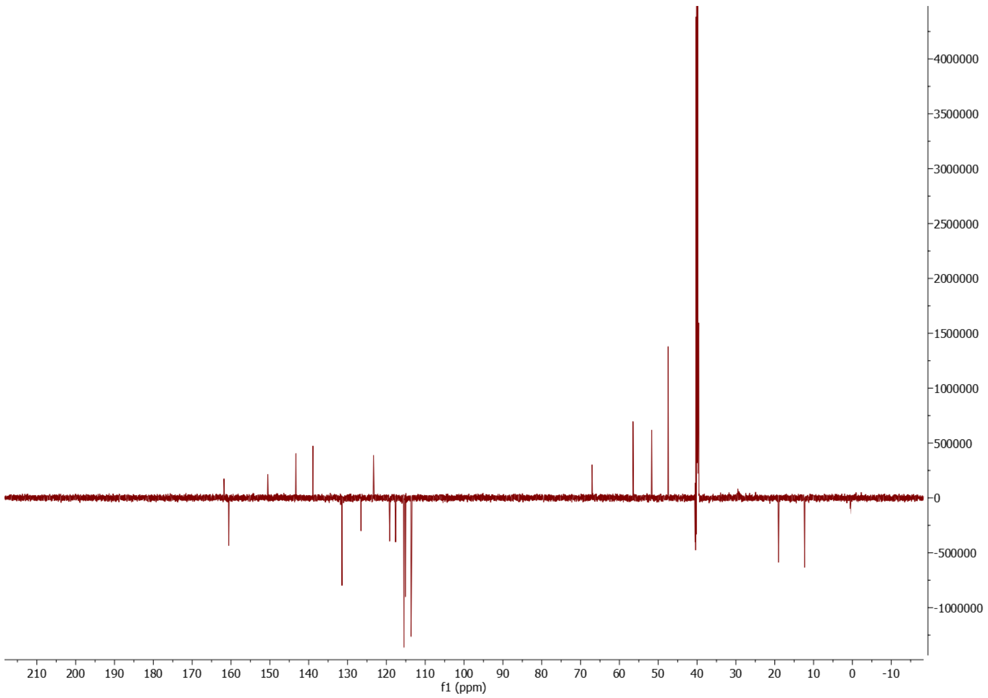

Figure S26. ^{13}C NMR (APT) spectrum of compound **13**

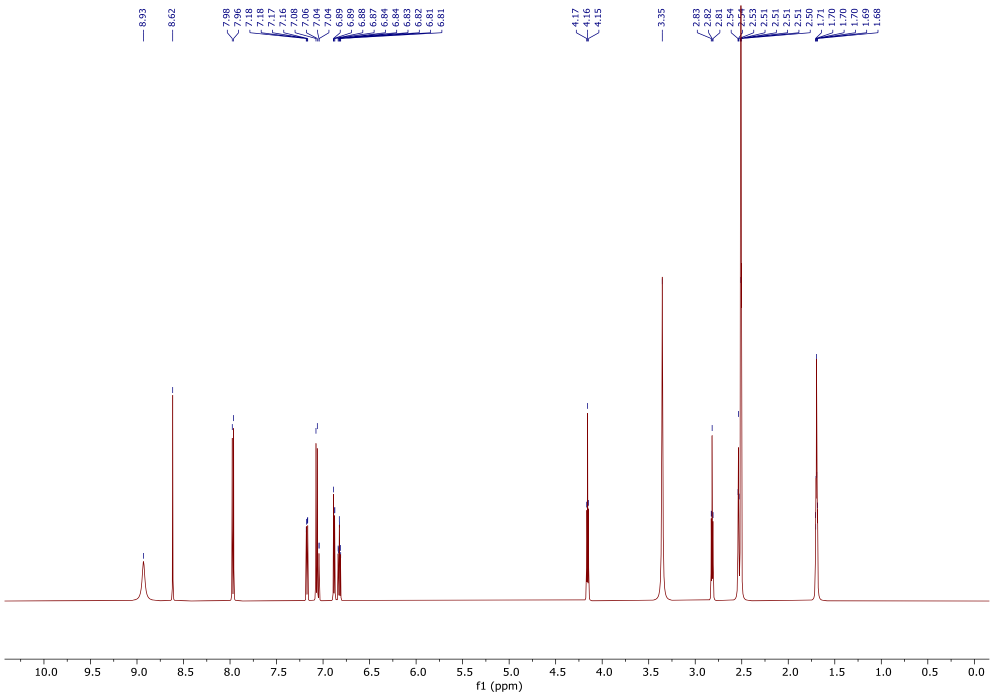

Figure S27. ^{1}H NMR spectrum of compound **14**

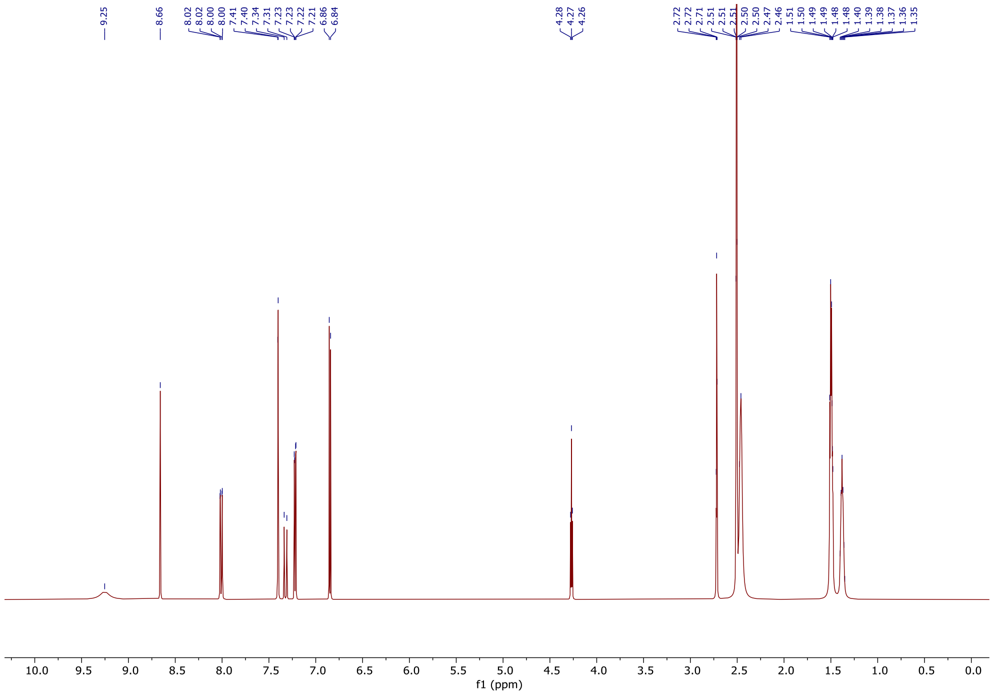

Figure S28. ^{1}H NMR spectrum of compound **15**

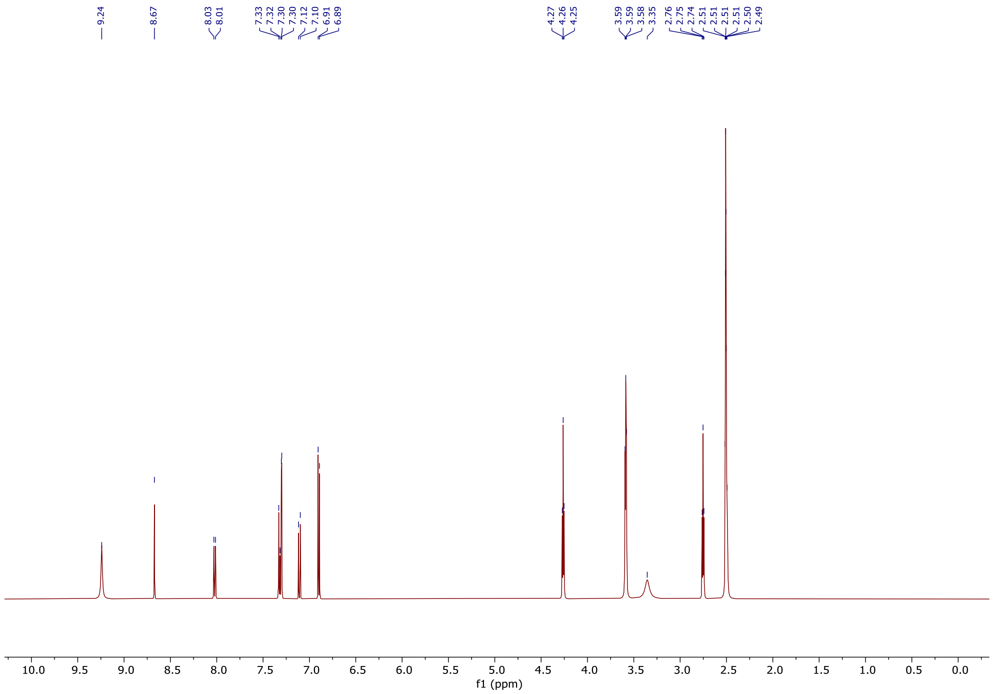

Figure S29. ^{1}H NMR spectrum of compound **16**

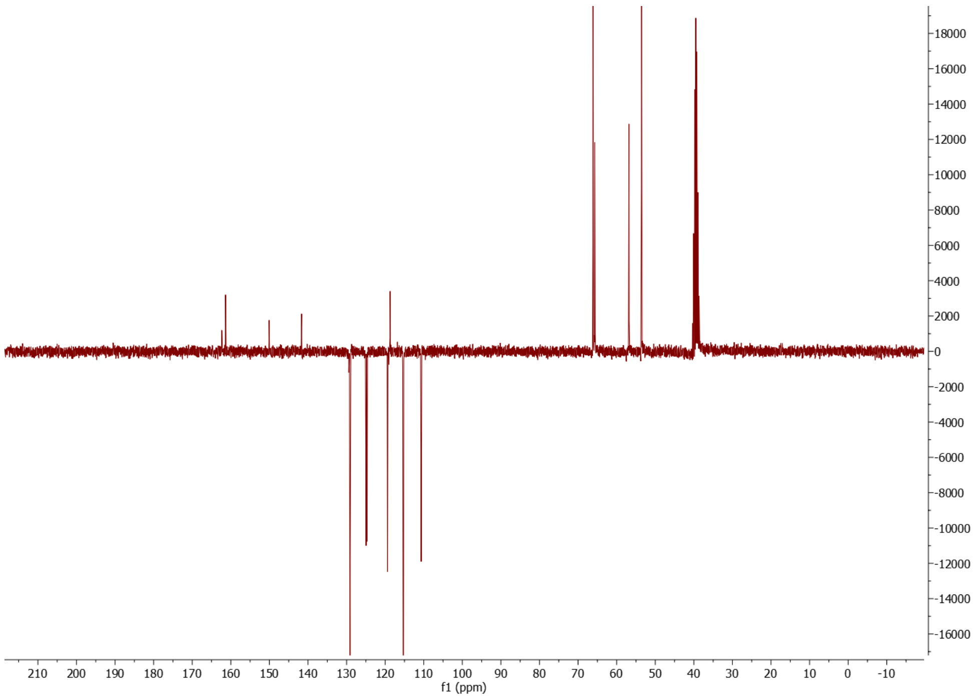

Figure S30. ^{13}C NMR (APT) spectrum of compound **16**

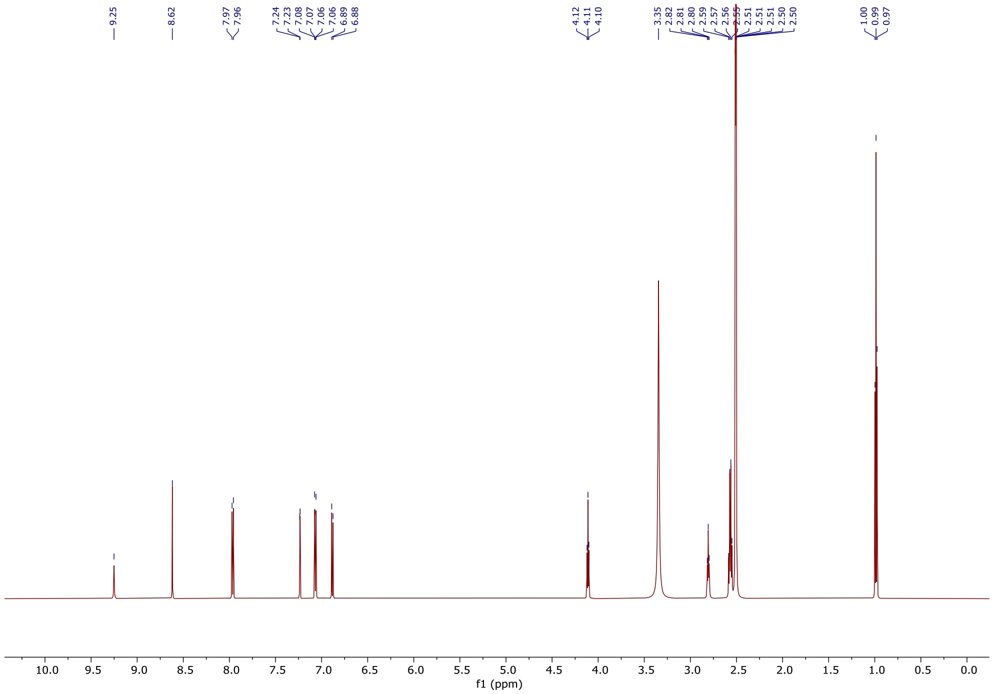

Figure S31. ^{1}H NMR spectrum of compound **17**

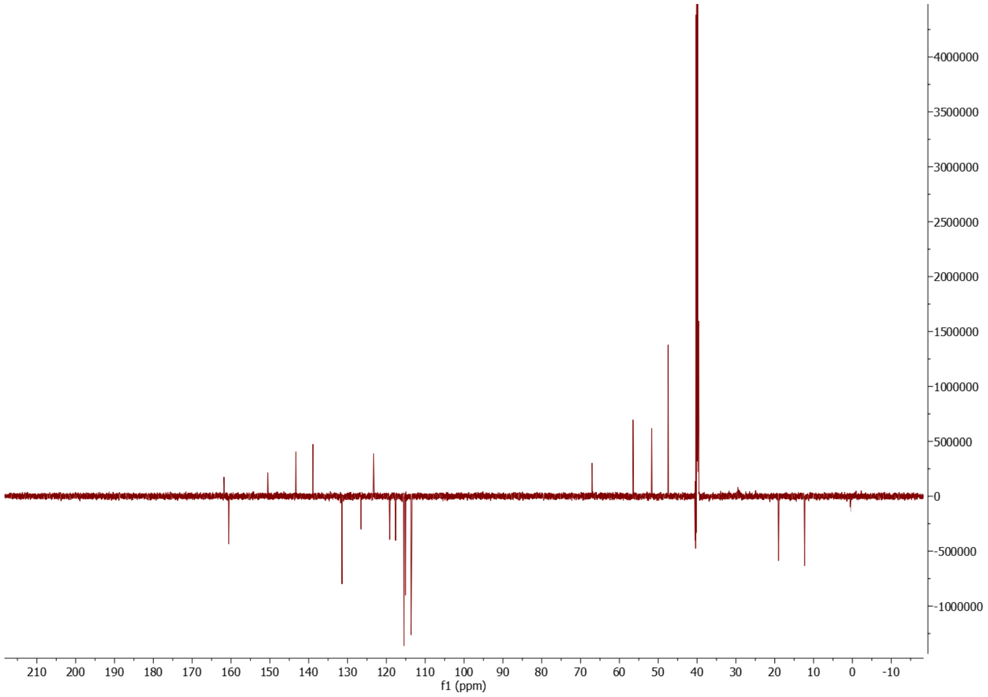

Figure S32. ^{13}C NMR (APT) spectrum of compound **17**

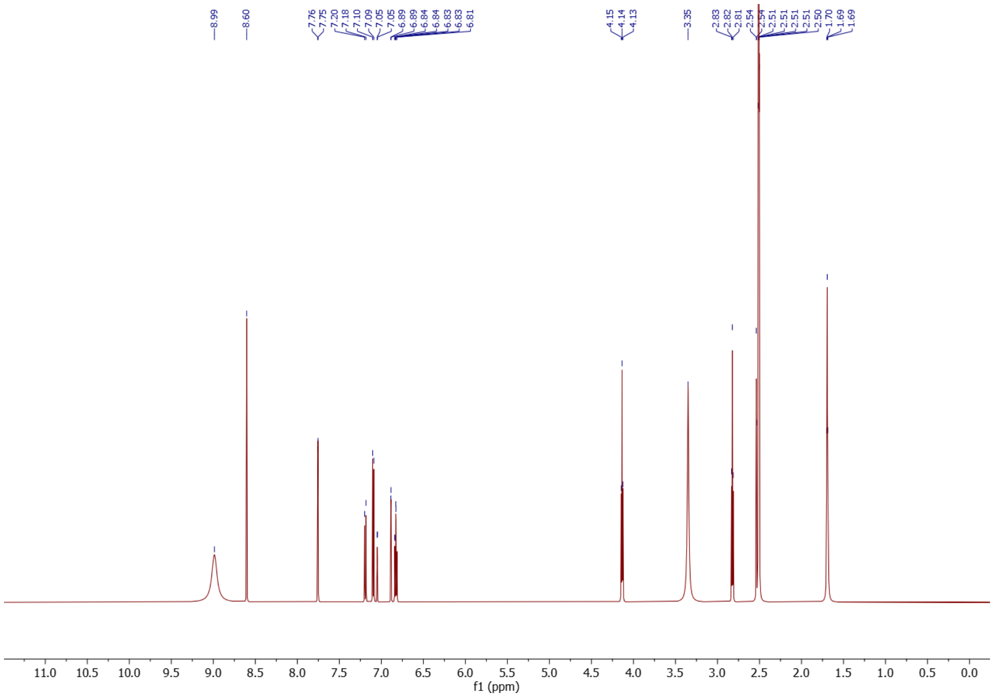

Figure S33. ^{1}H NMR spectrum of compound **18**

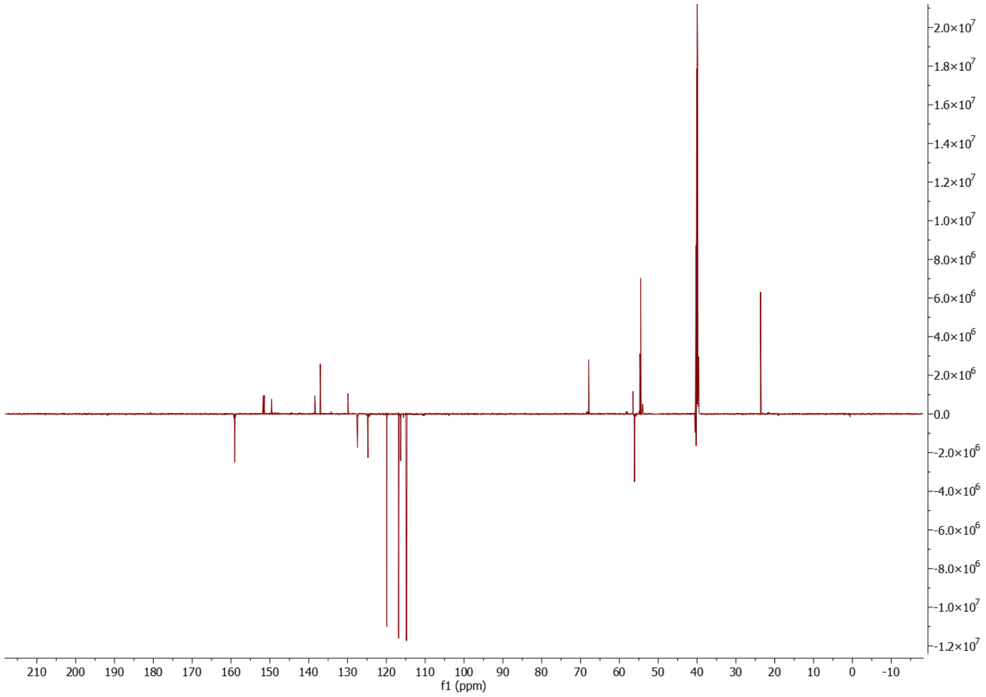

Figure S34. ^{13}C NMR (APT) spectrum of compound **18**

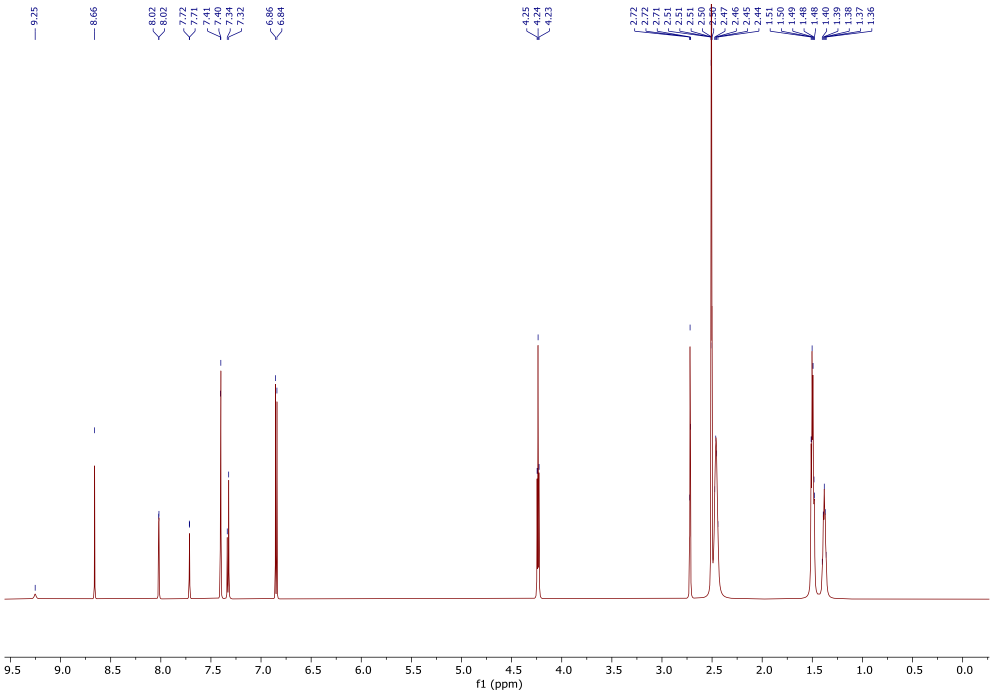

Figure S35. ^{1}H NMR spectrum of compound **19**

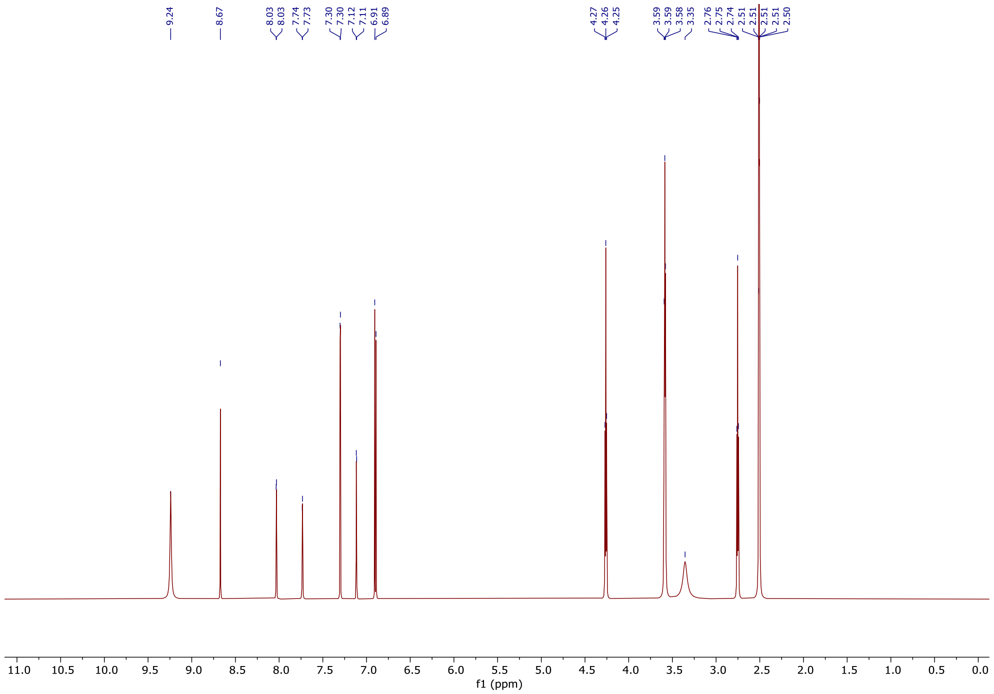

Figure S36. ^{1}H NMR spectrum of compound **20**

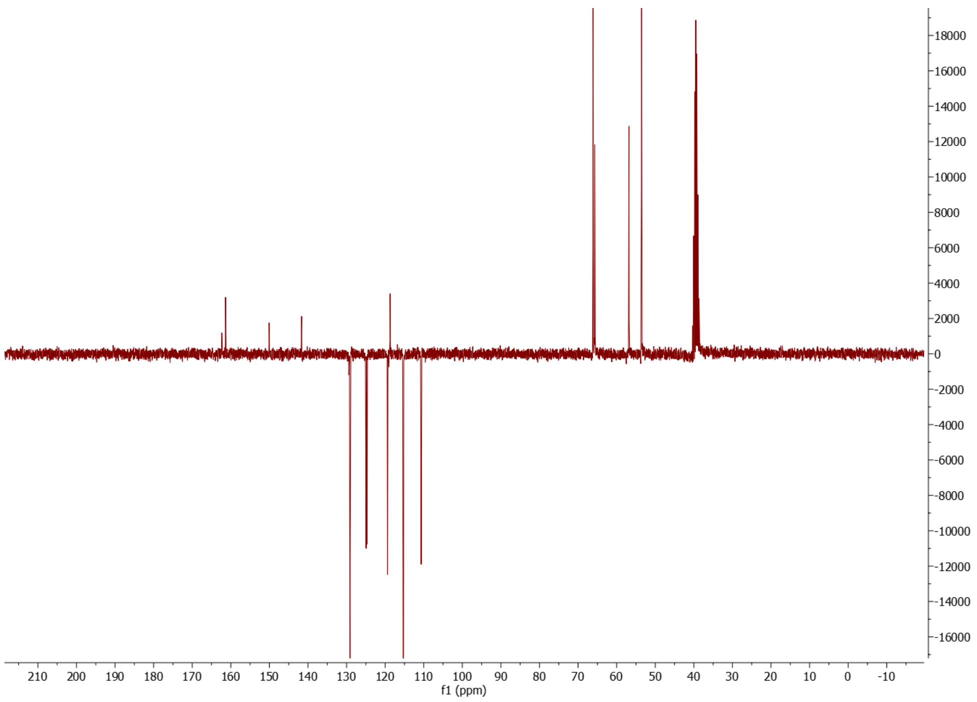

Figure S37. ^{13}C NMR (APT) spectrum of compound **20**

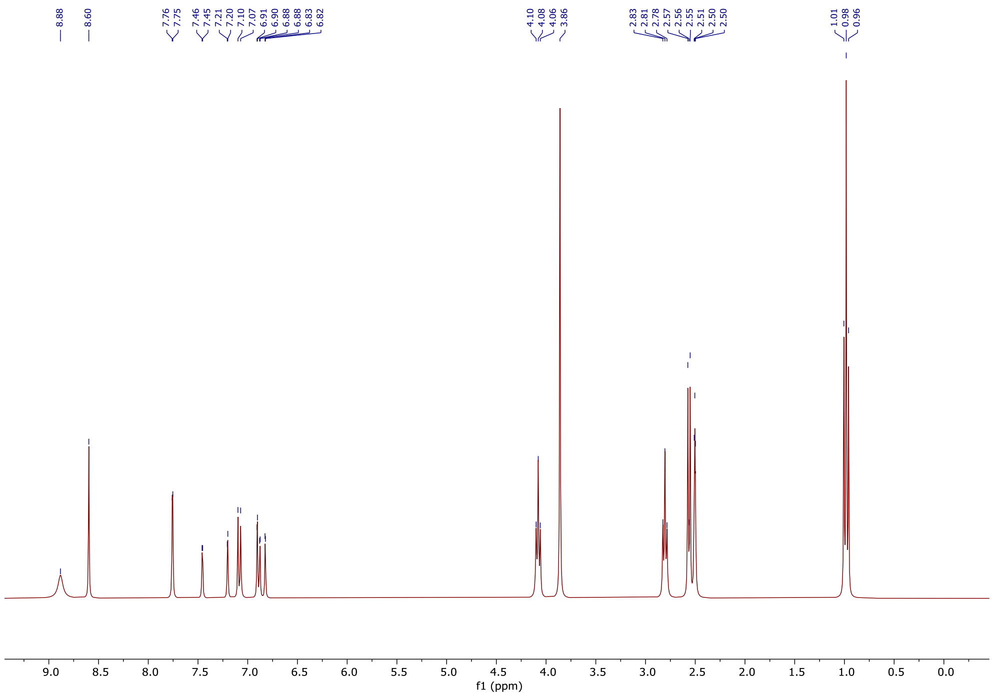

Figure S38. ^{1}H NMR spectrum of compound **21**

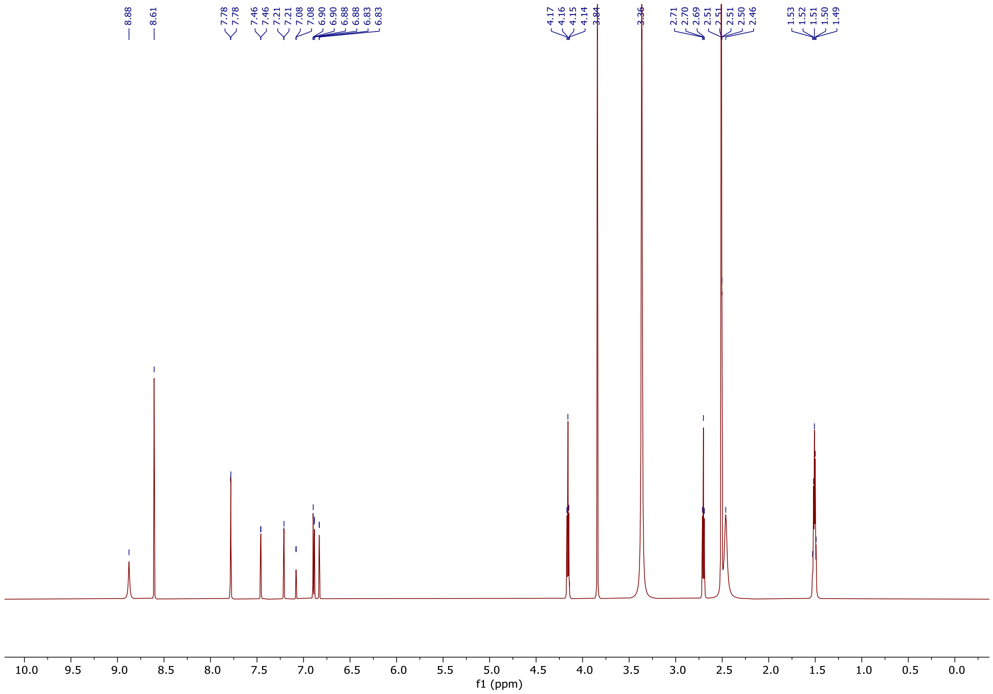

Figure S39. ^{1}H NMR spectrum of compound **22**

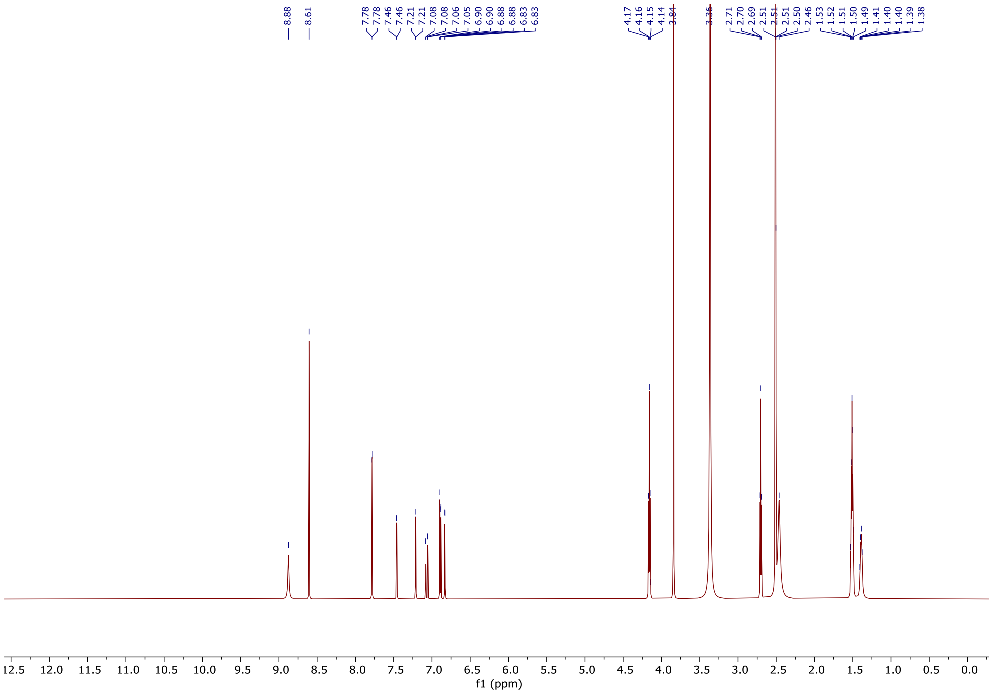

Figure S40. ^{1}H NMR spectrum of compound **23**

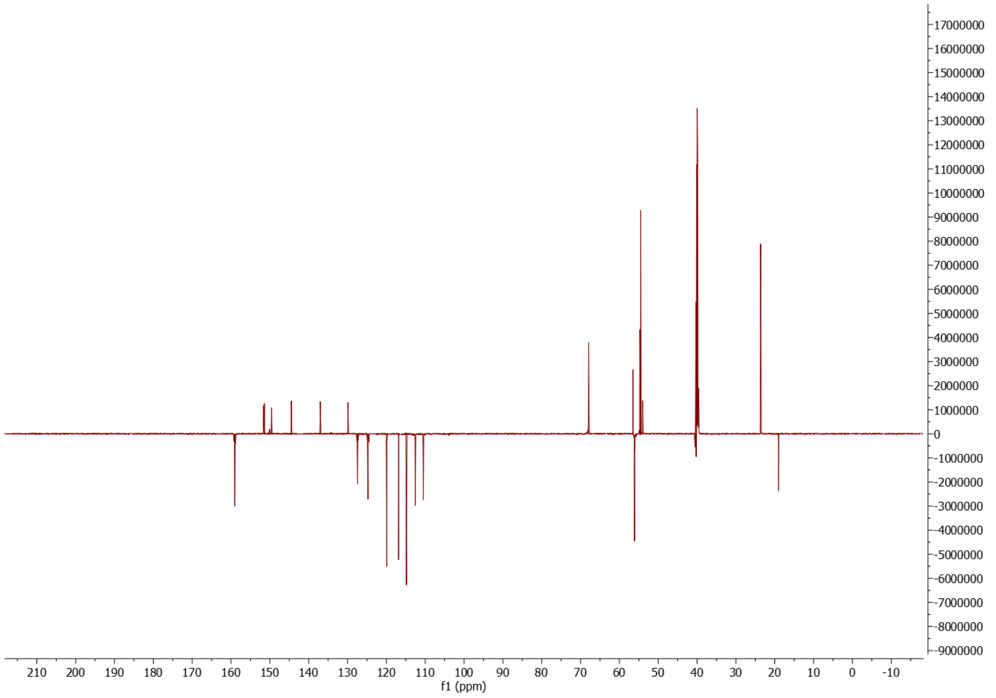

Figure S41. ^{13}C NMR (APT) spectrum of compound **23**

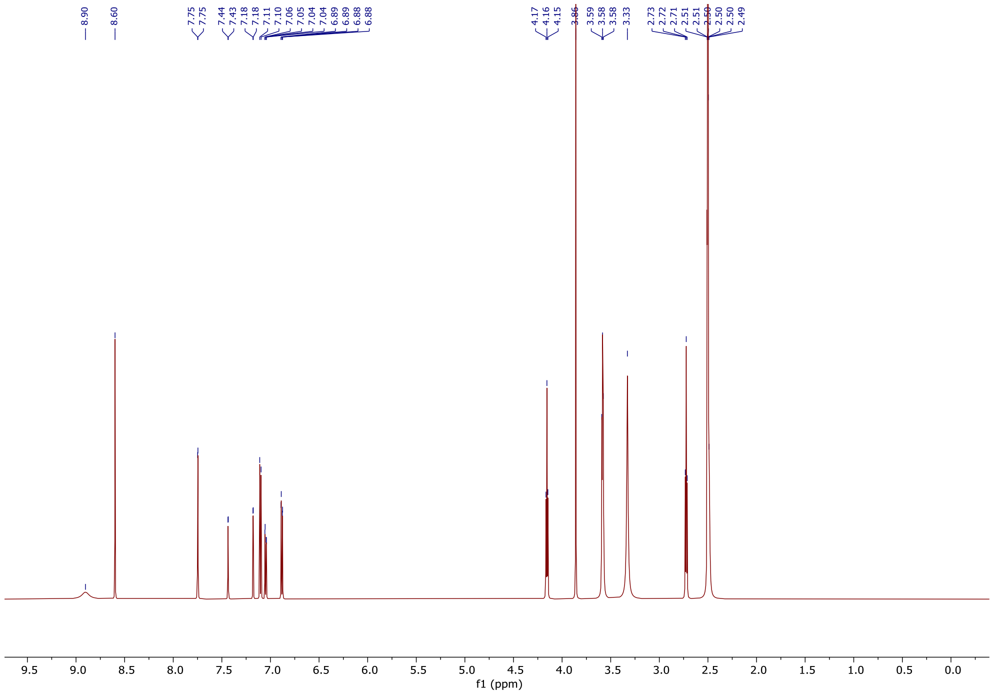

Figure S42. ^{1}H NMR spectrum of compound **24**

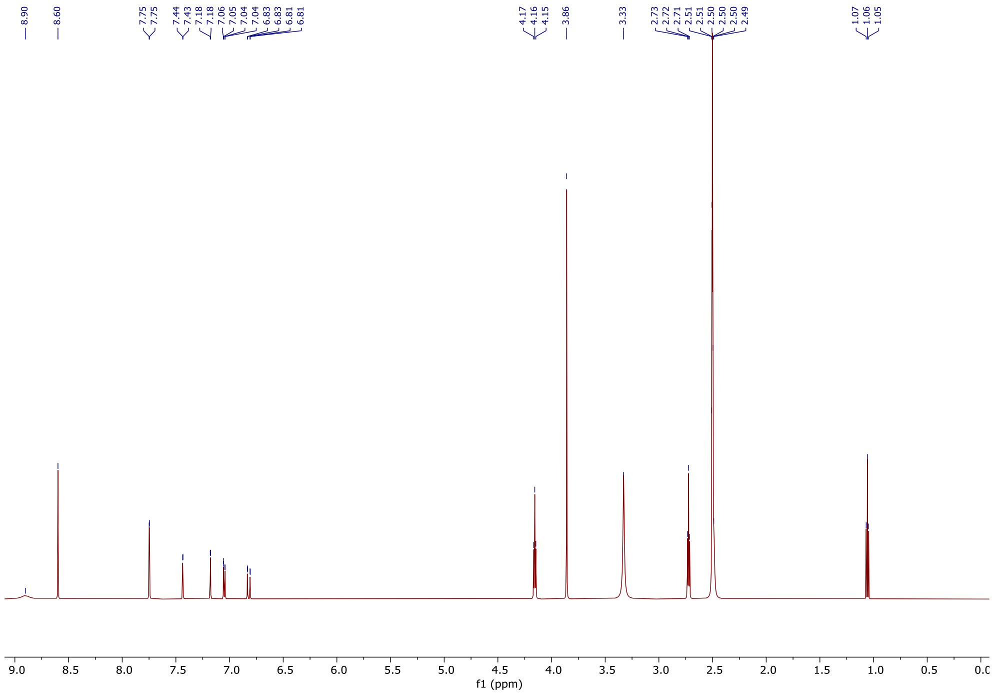

Figure S43. ^{1}H NMR spectrum of compound **25**

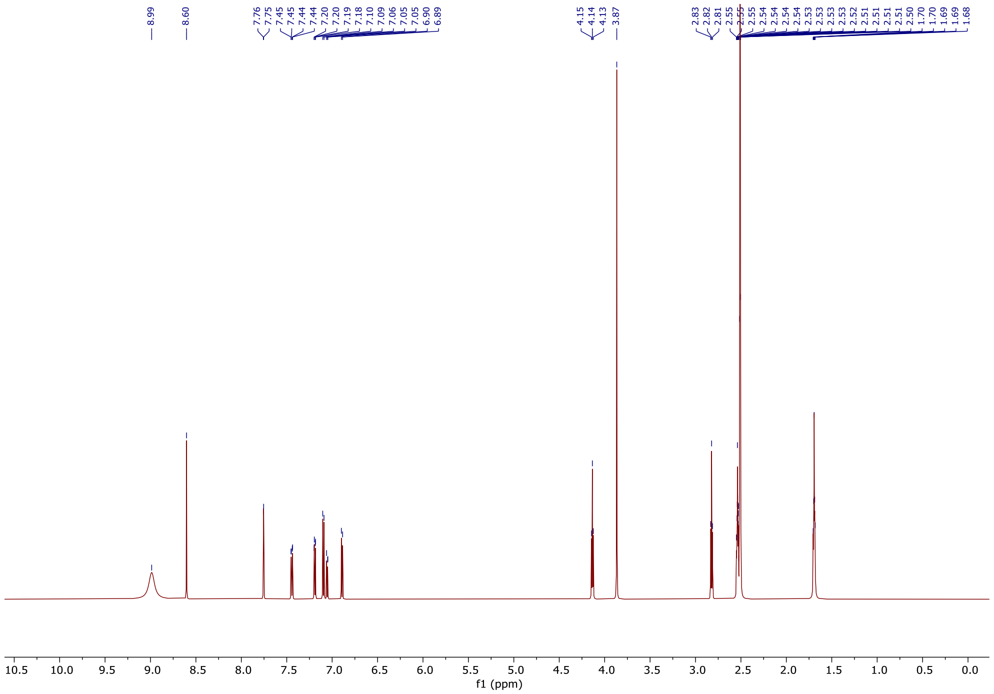

Figure S44. ^{1}H NMR spectrum of compound **26**

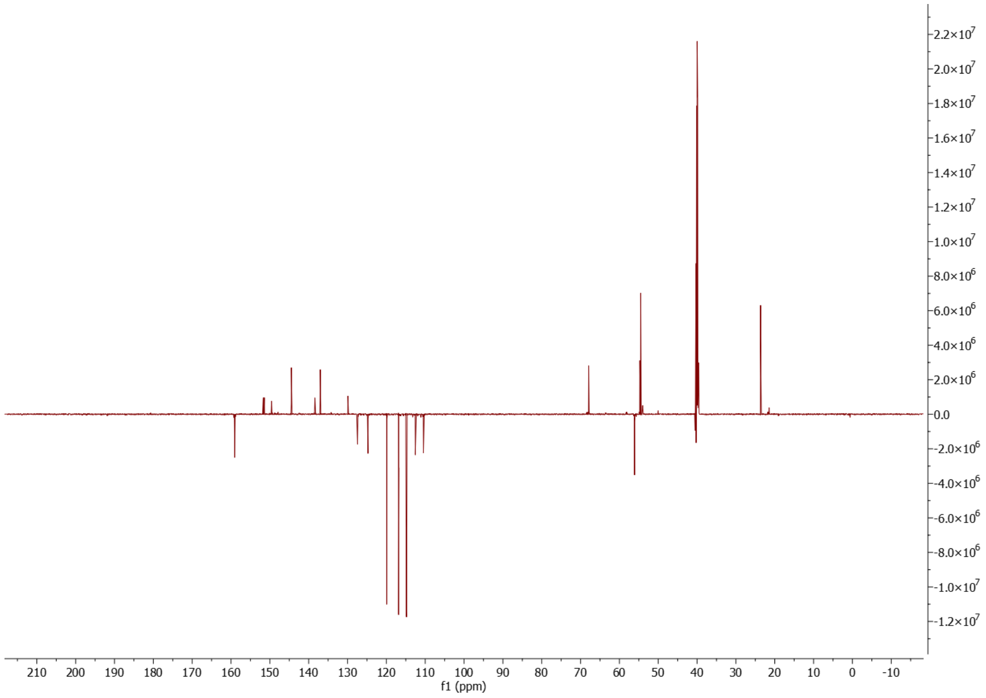

Figure S45. ^{13}C NMR (APT) spectrum of compound **26**

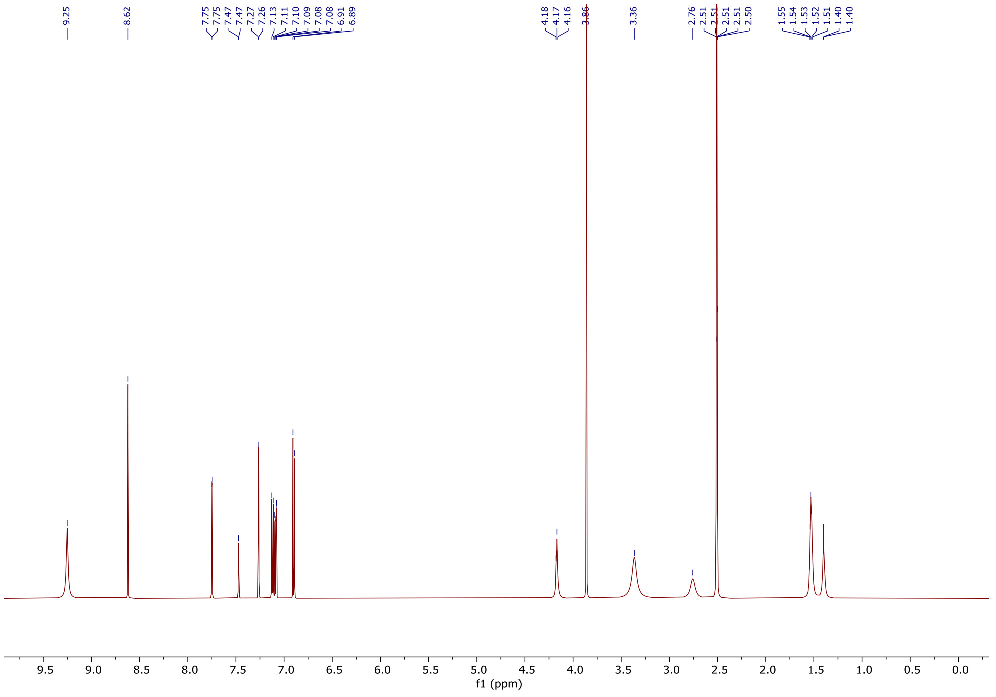

Figure S46. ^{1}H NMR spectrum of compound **27**

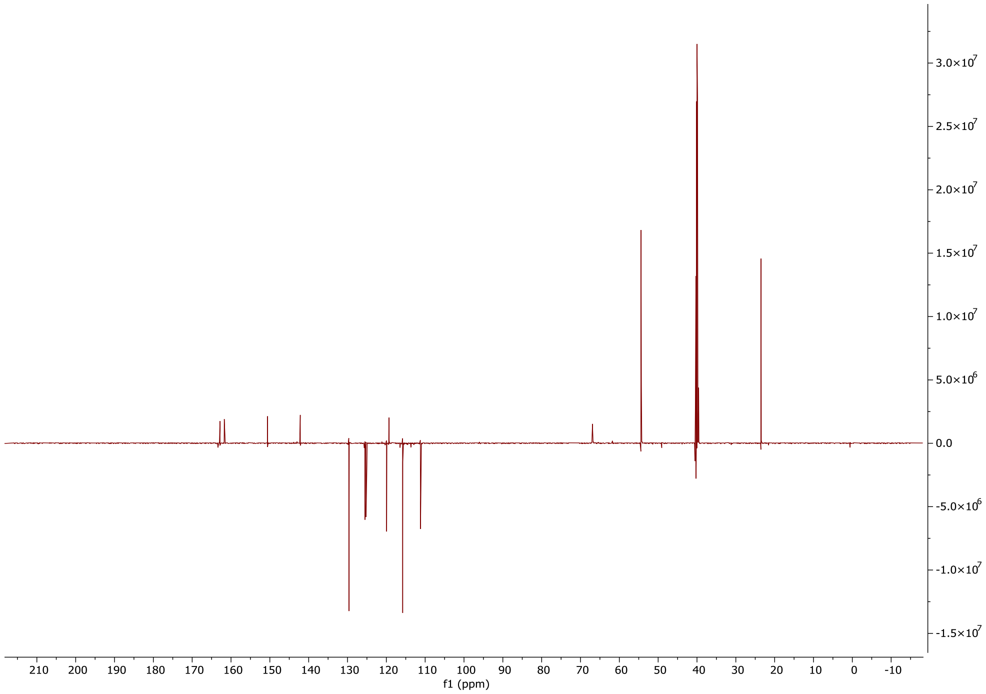

Figure S47. ^{13}C NMR (APT) spectrum of compound **27**

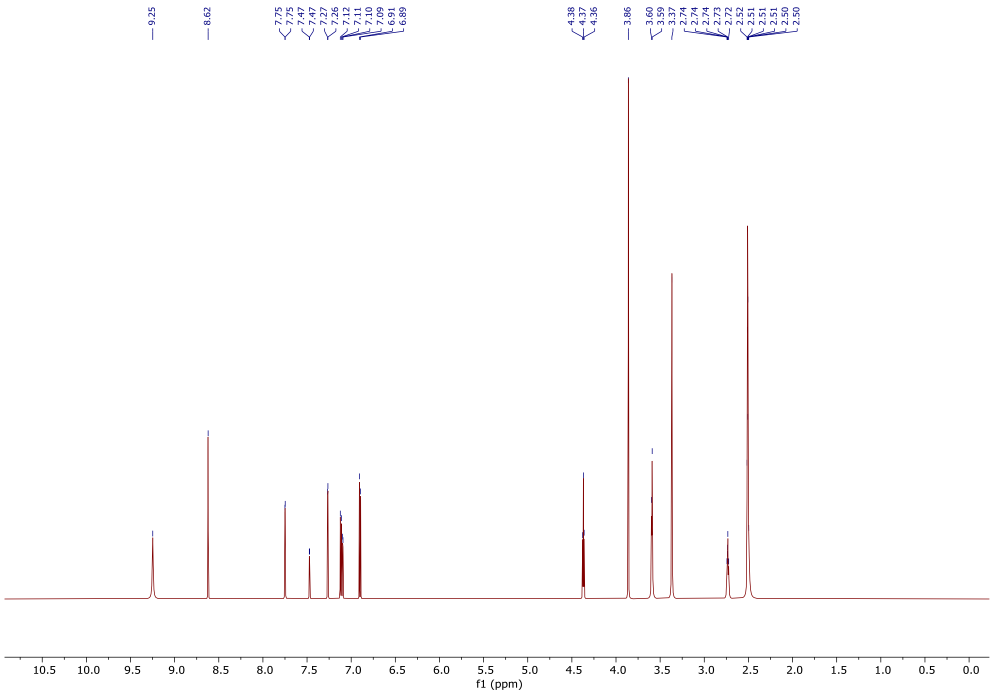

Figure S48. ^{1}H NMR spectrum of compound **28**

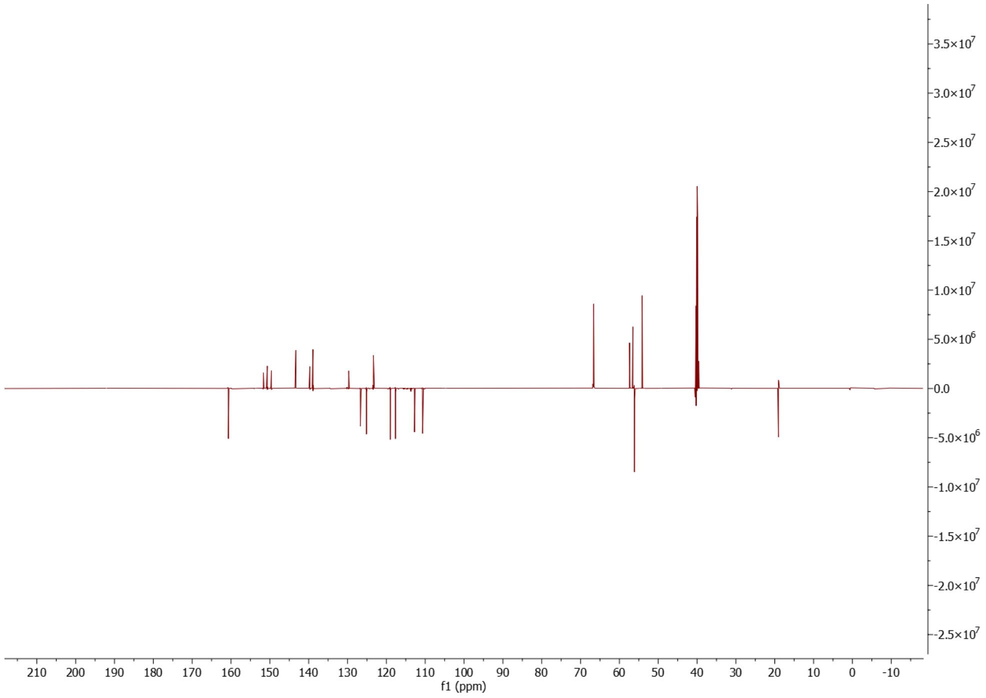

Figure S49. ^{13}C NMR (APT) spectrum of compound **28**

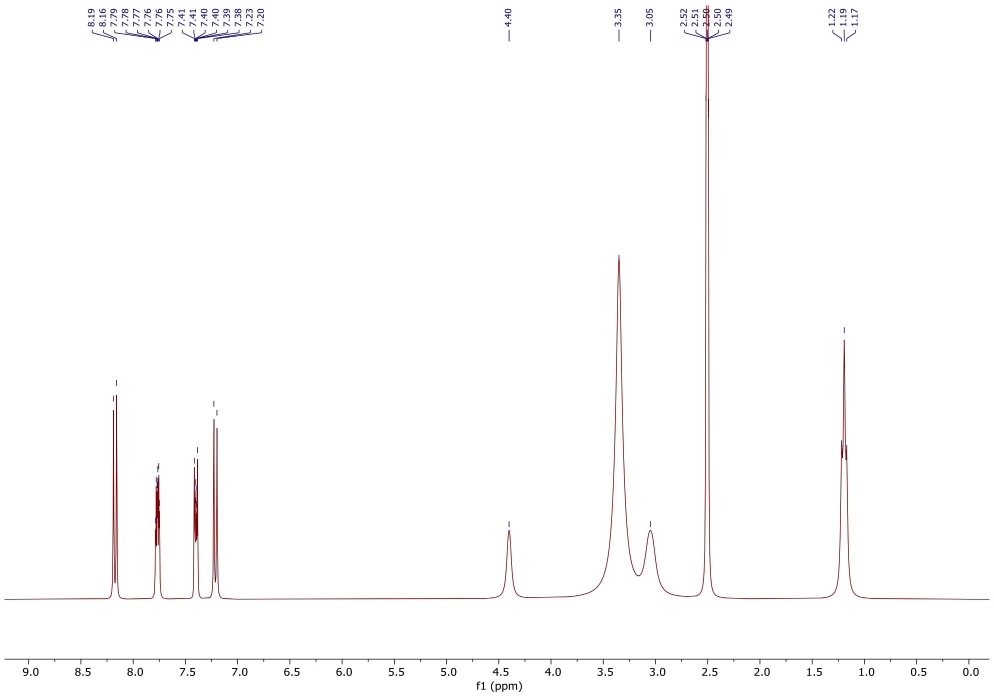

Figure S50. ^{1}H NMR spectrum of compound **29**

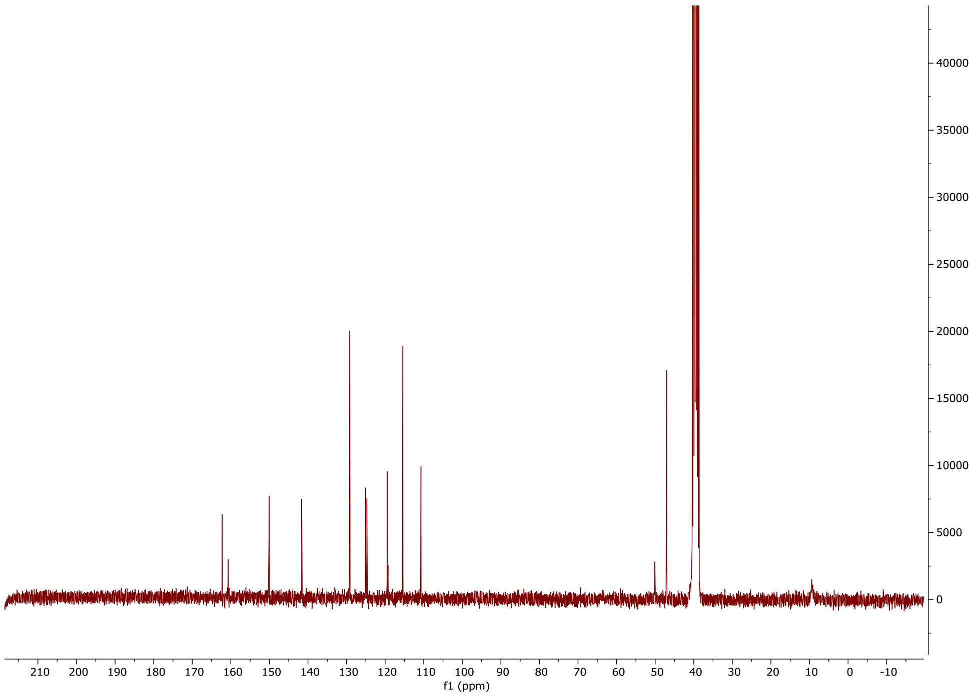

Figure S51. ^{13}C NMR spectrum of compound **29**

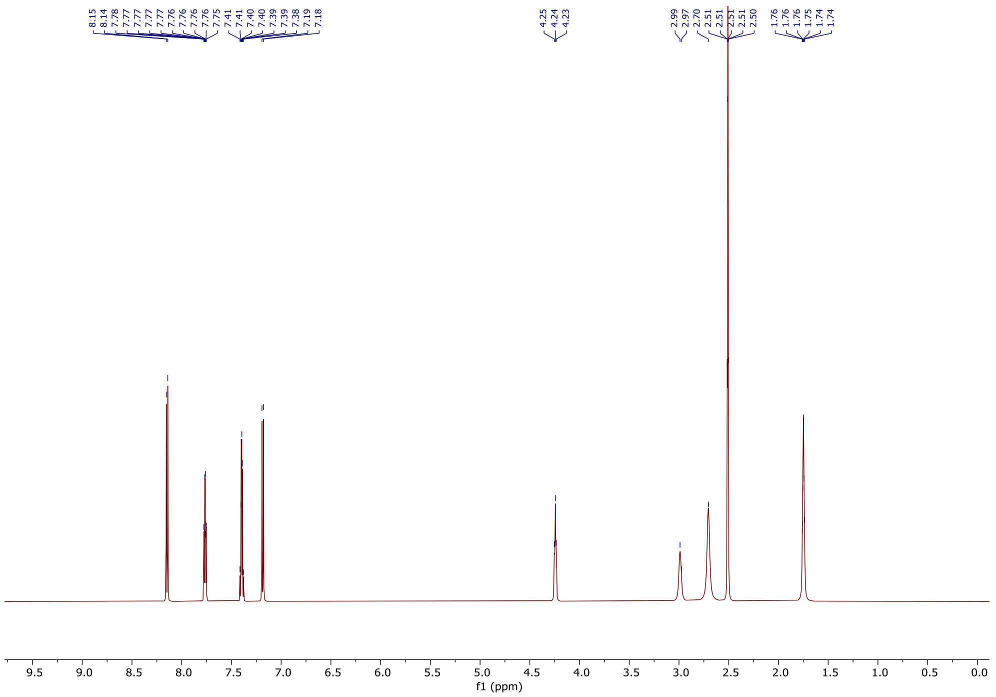

Figure S52. ^{1}H NMR spectrum of compound **30**

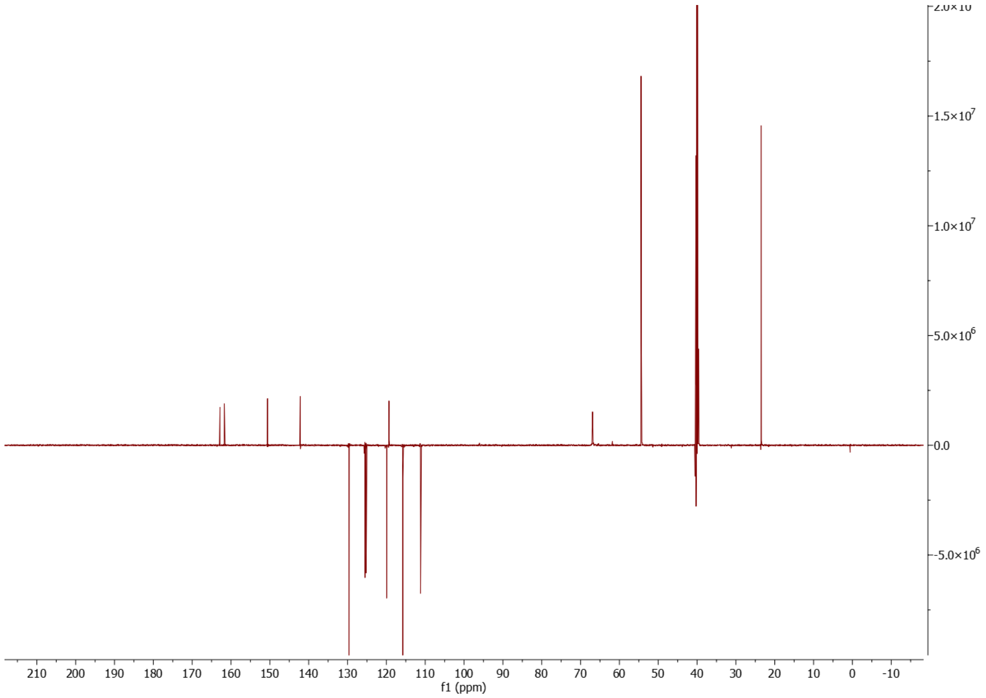

Figure S53. ^{13}C NMR (APT) spectrum of compound **30**

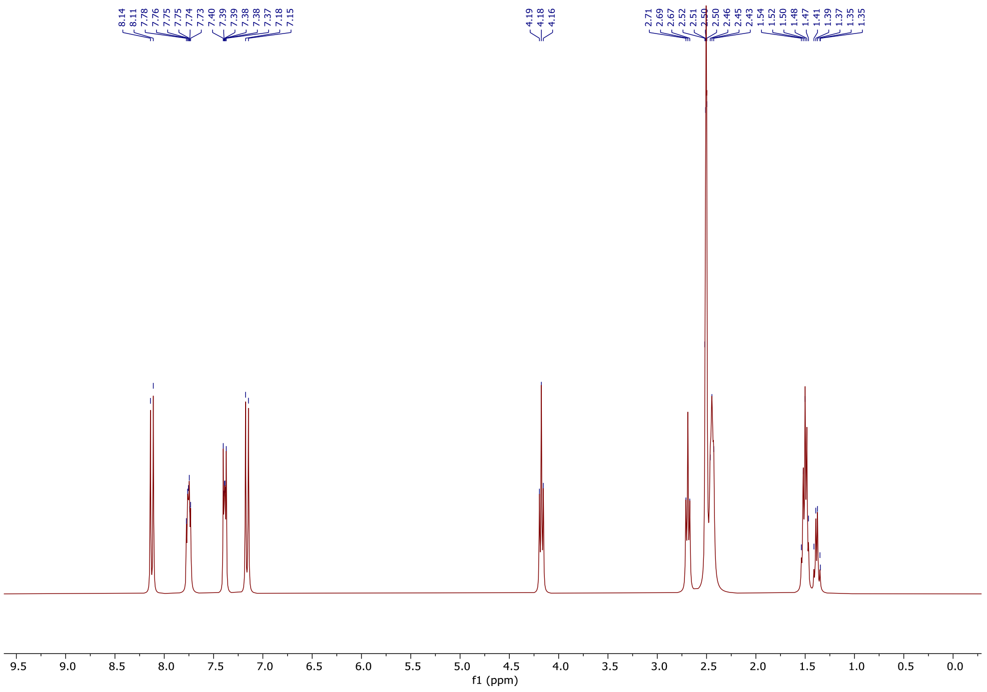

Figure S54. ^{1}H NMR spectrum of compound **31**

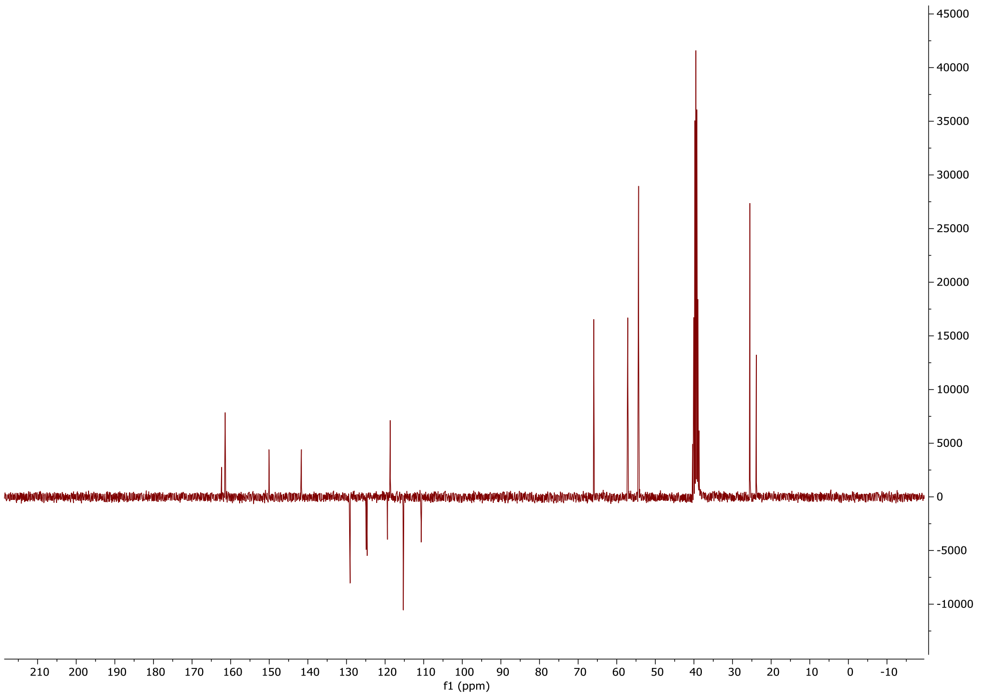

Figure S55. ^{13}C NMR (APT) spectrum of compound **31**

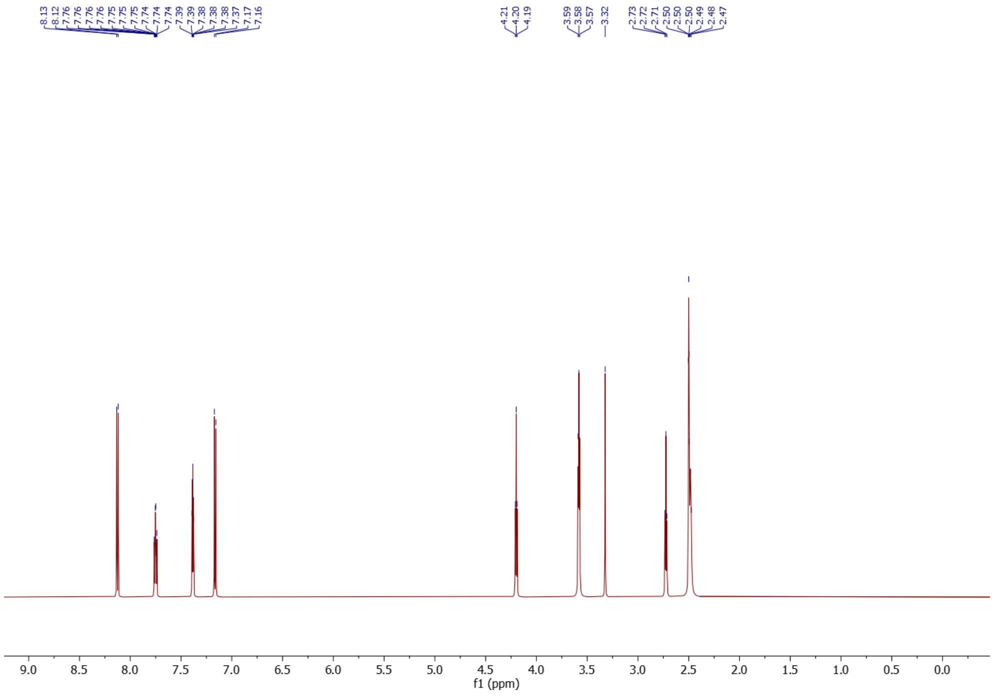

Figure S56. ^{1}H NMR spectrum of compound **32**

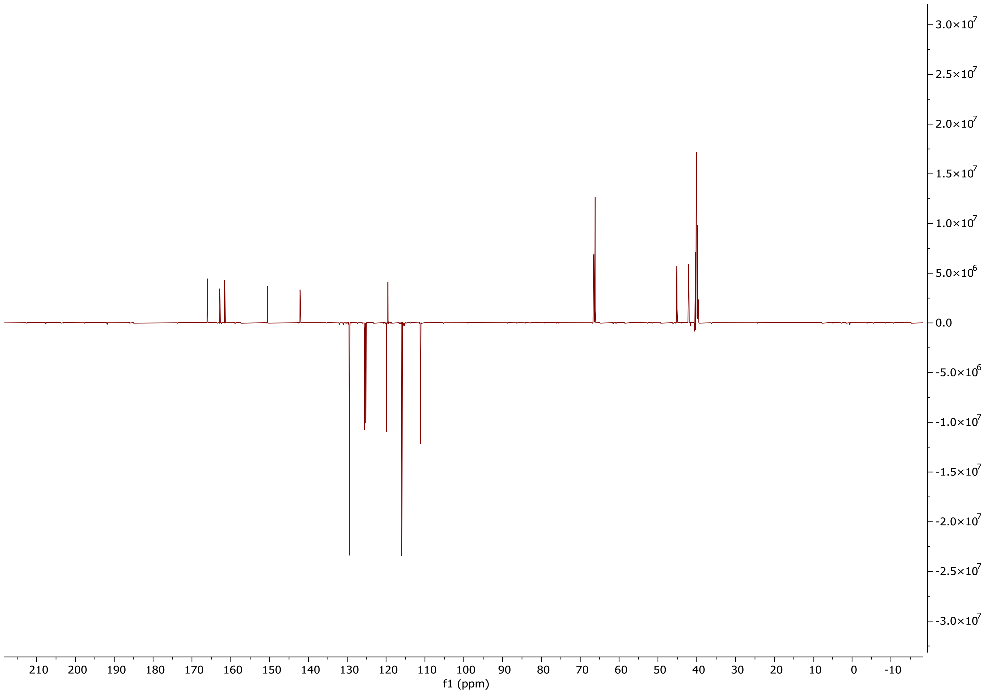

Figure S57. ^{13}C NMR (APT) spectrum of compound **32**

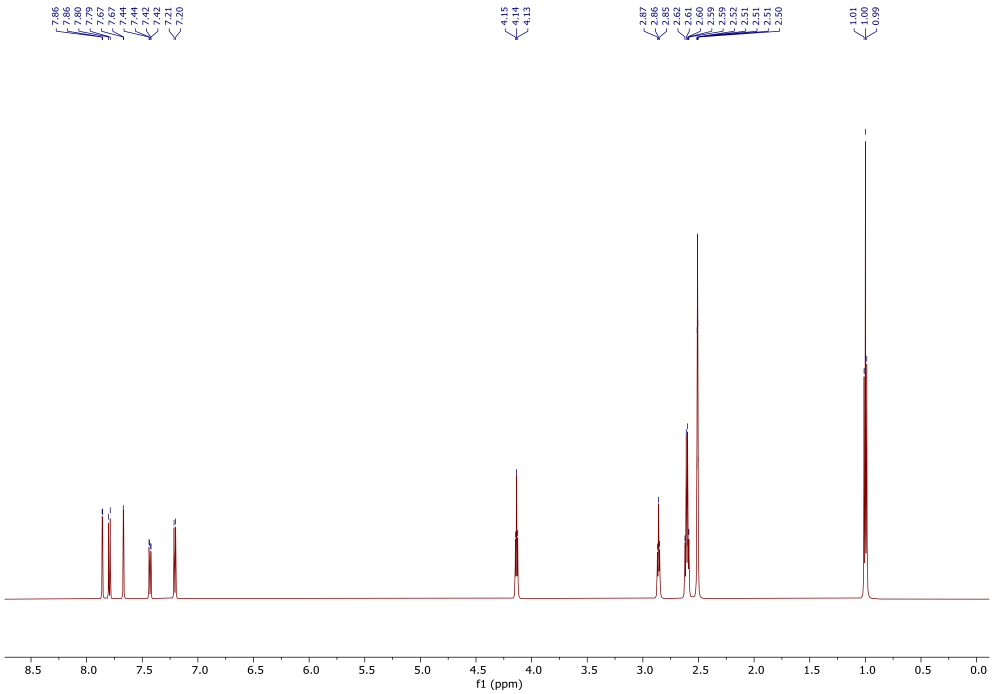

Figure S58. ^{1}H NMR spectrum of compound **33**

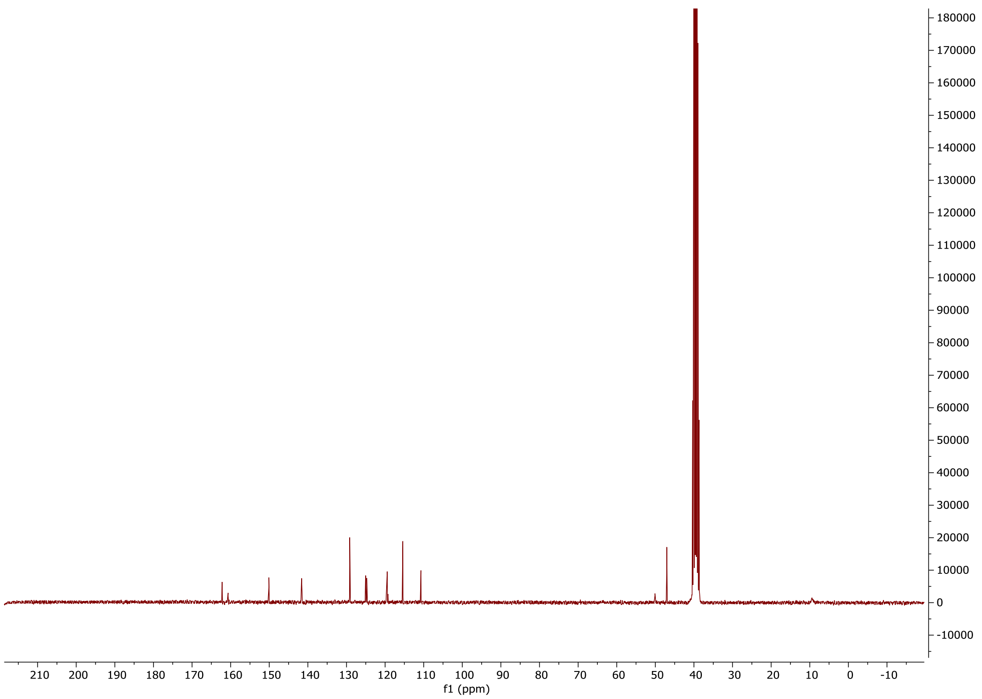

Figure S59. ^{13}C NMR spectrum of compound **33**

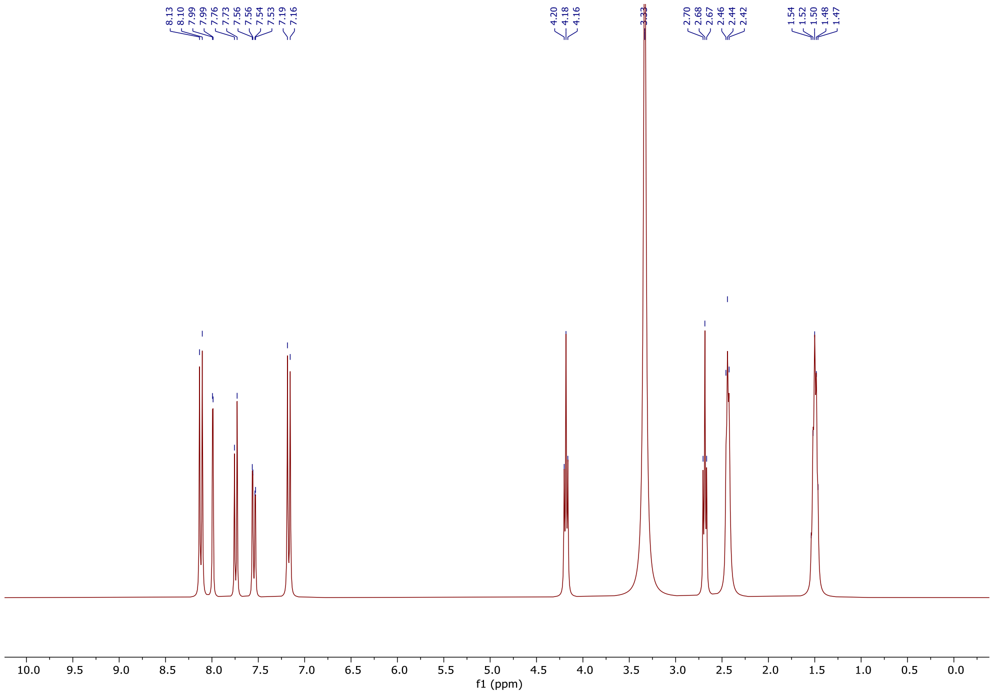

Figure S60. ^{1}H NMR spectrum of compound **34**

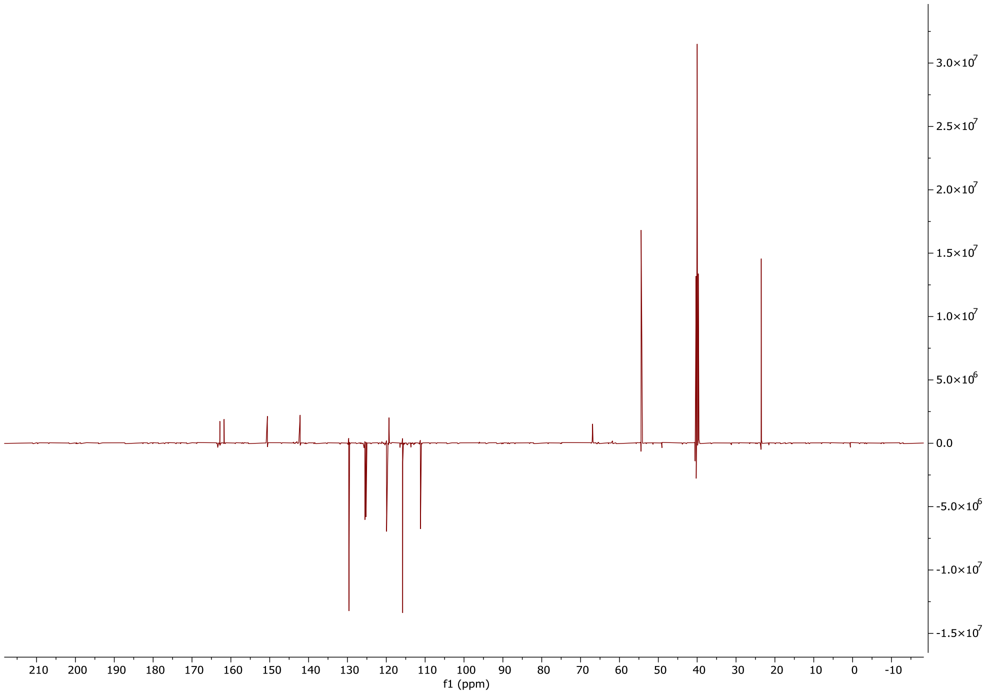

Figure S61. ^{13}C NMR (APT) spectrum of compound **34**

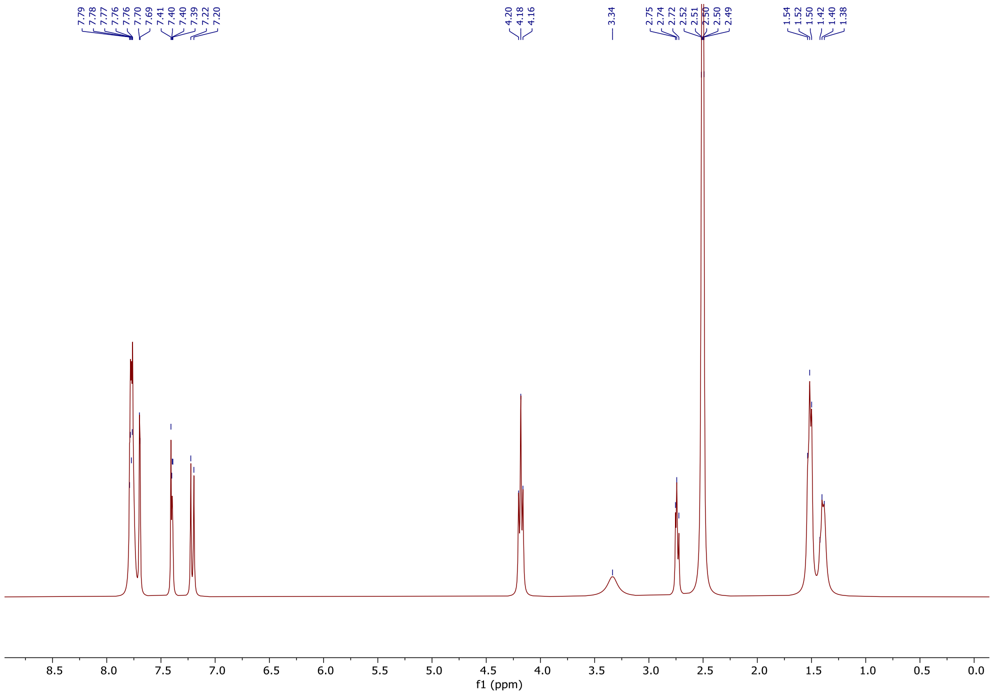

Figure S62. ^{1}H NMR spectrum of compound **35**

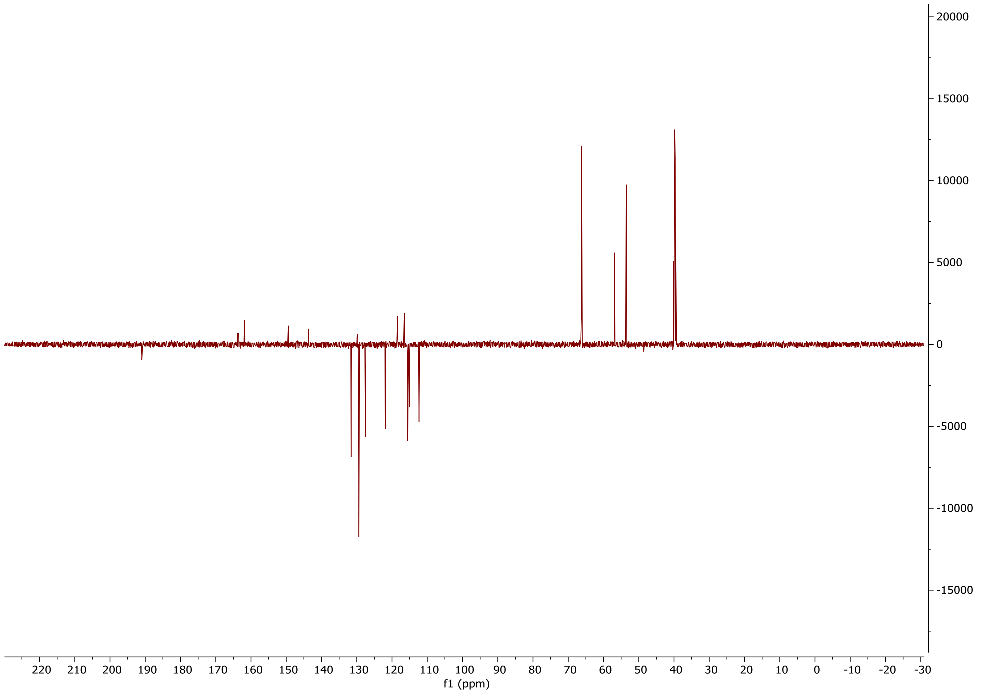

Figure S63. ^{13}C NMR (APT) spectrum of compound **35**

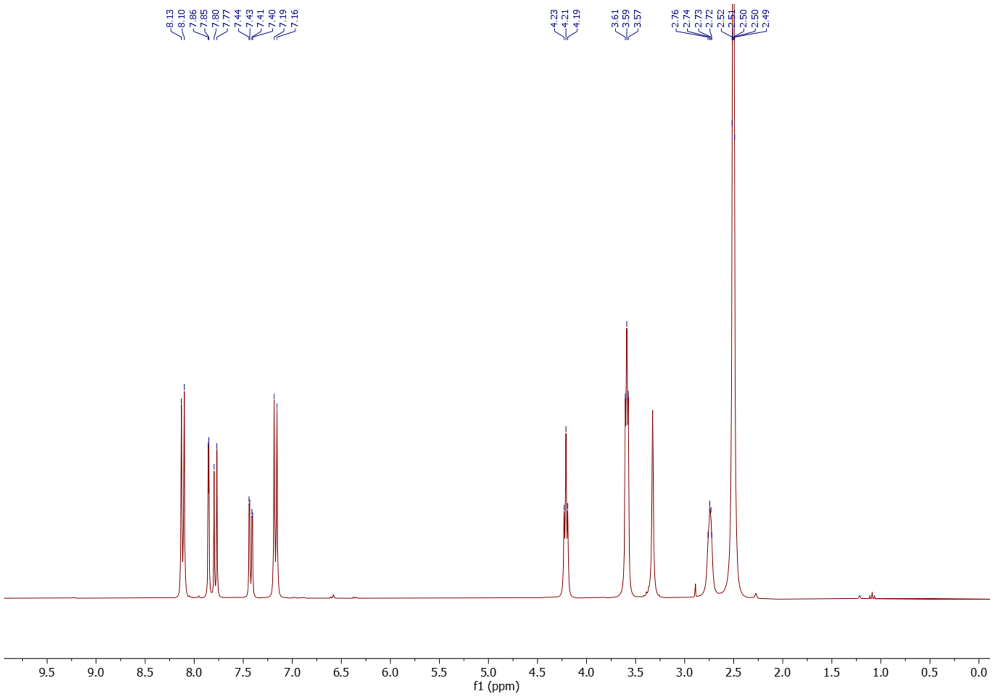

Figure S64. ^{1}H NMR spectrum of compound **36**

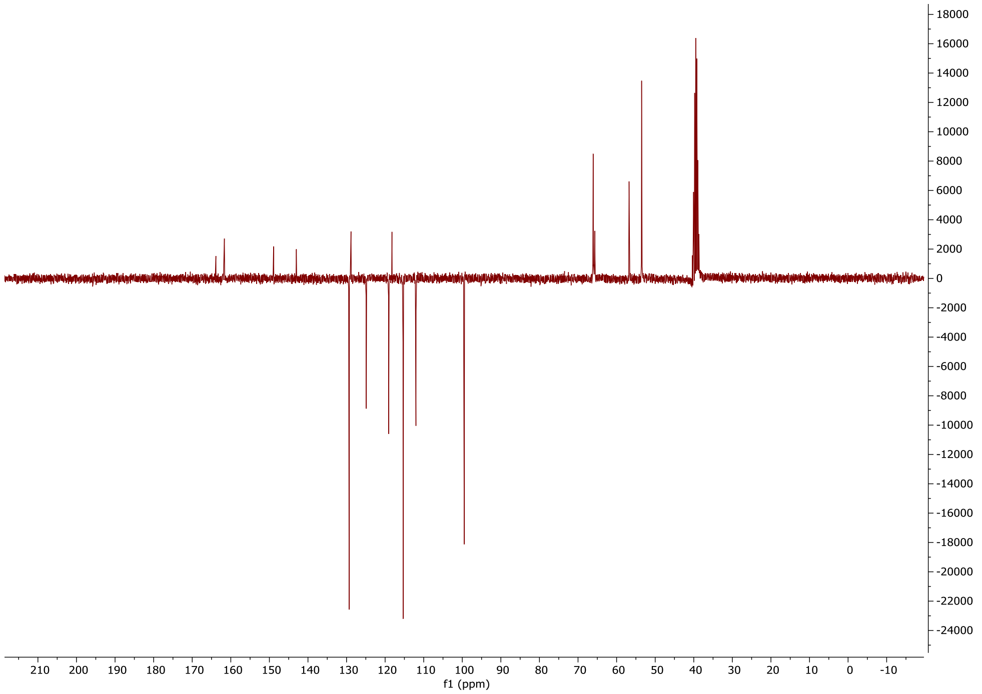

Figure S65. ^{13}C NMR (APT) spectrum of compound **36**

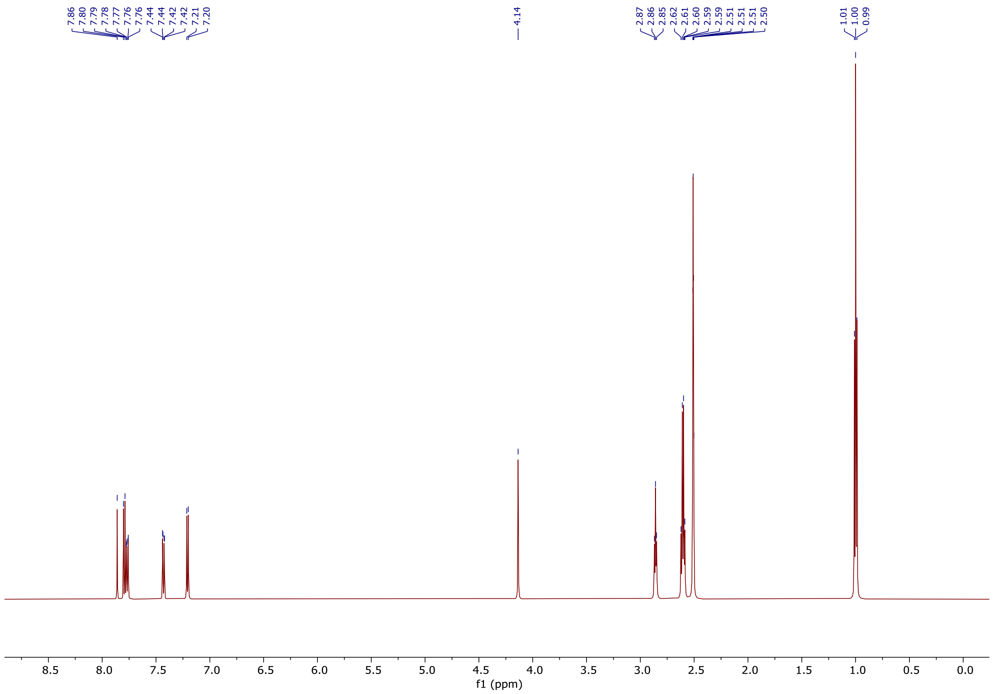

Figure S66. ^{1}H NMR spectrum of compound **37**

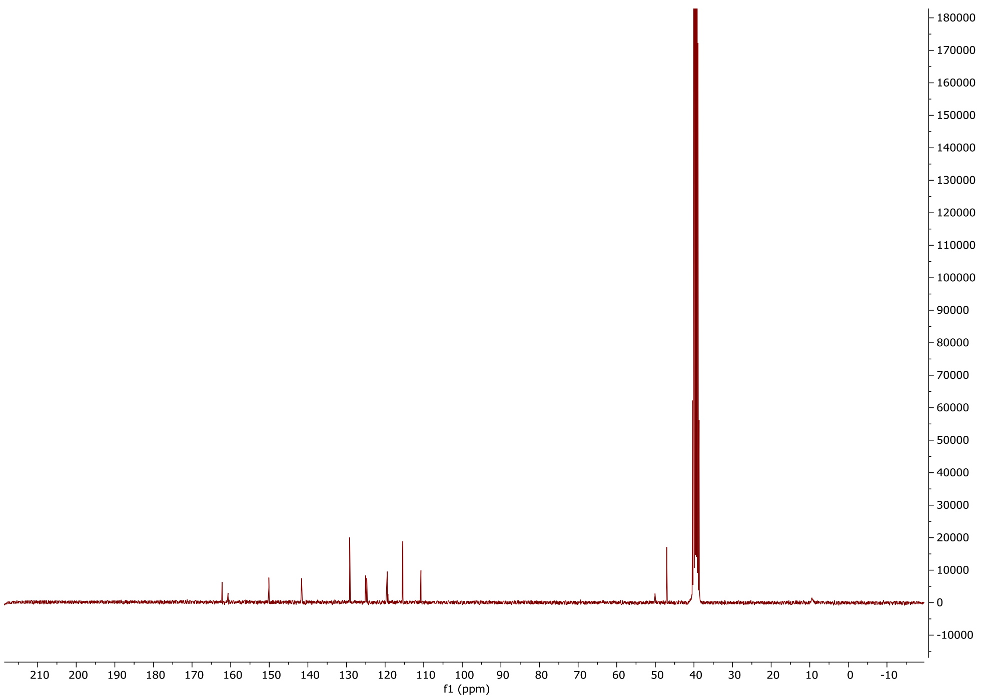

Figure S67. ^{13}C NMR spectrum of compound **37**

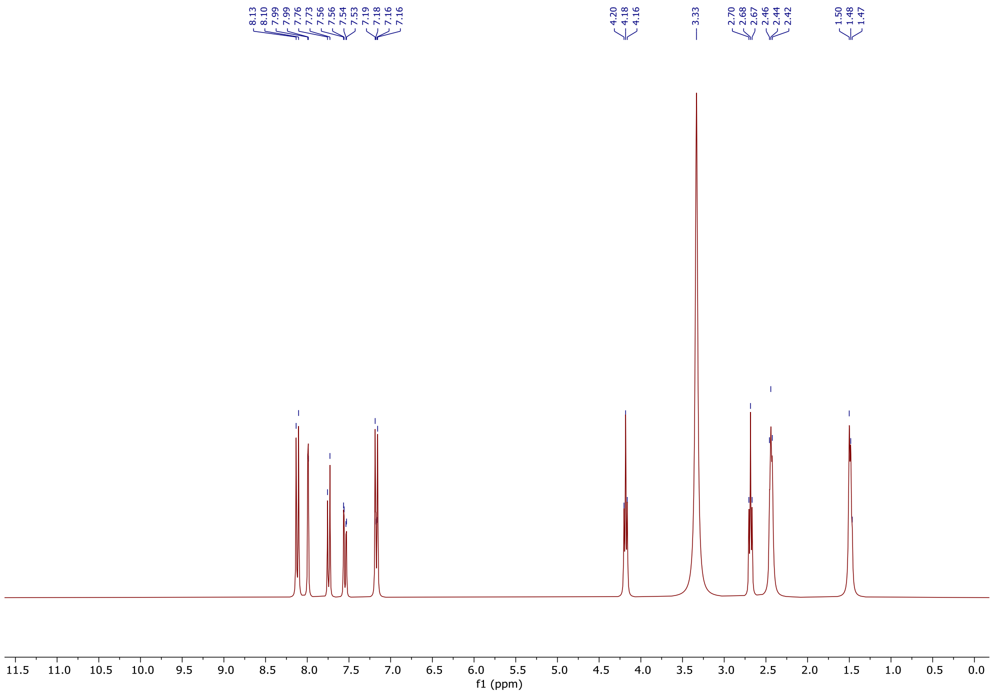

Figure S68. ^{1}H NMR spectrum of compound **38**

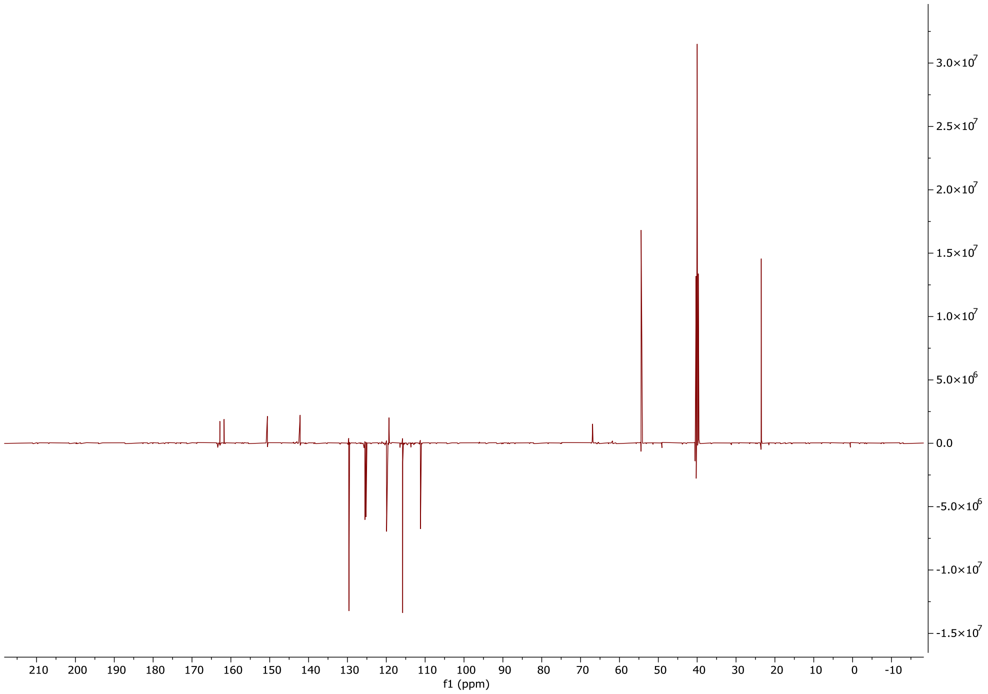

Figure S69. ^{13}C NMR (APT) spectrum of compound **38**

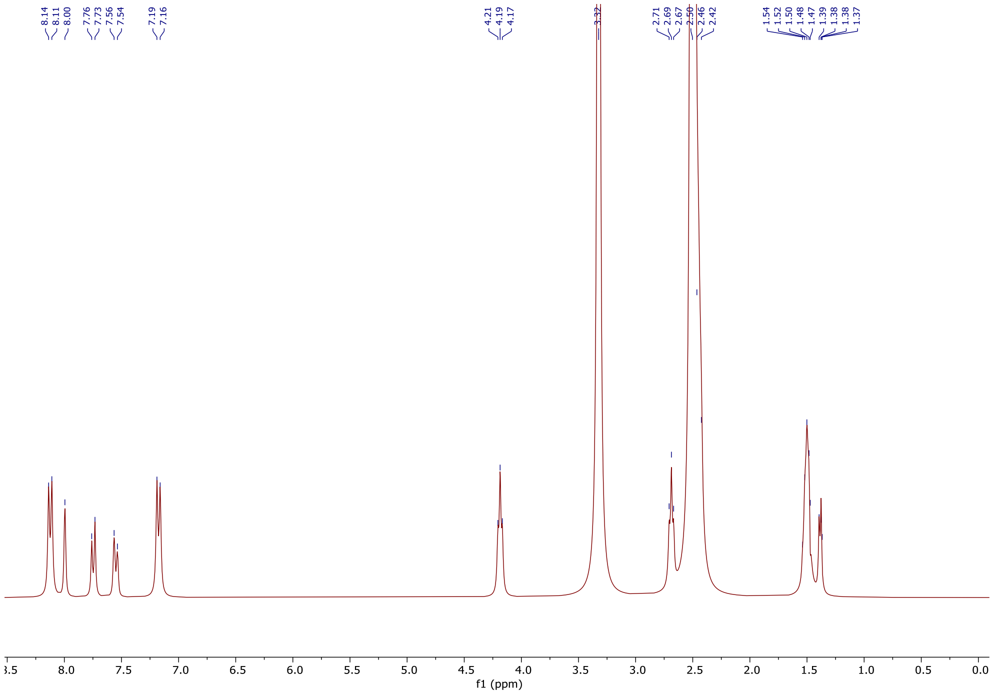

Figure S70. ^{1}H NMR spectrum of compound **39**

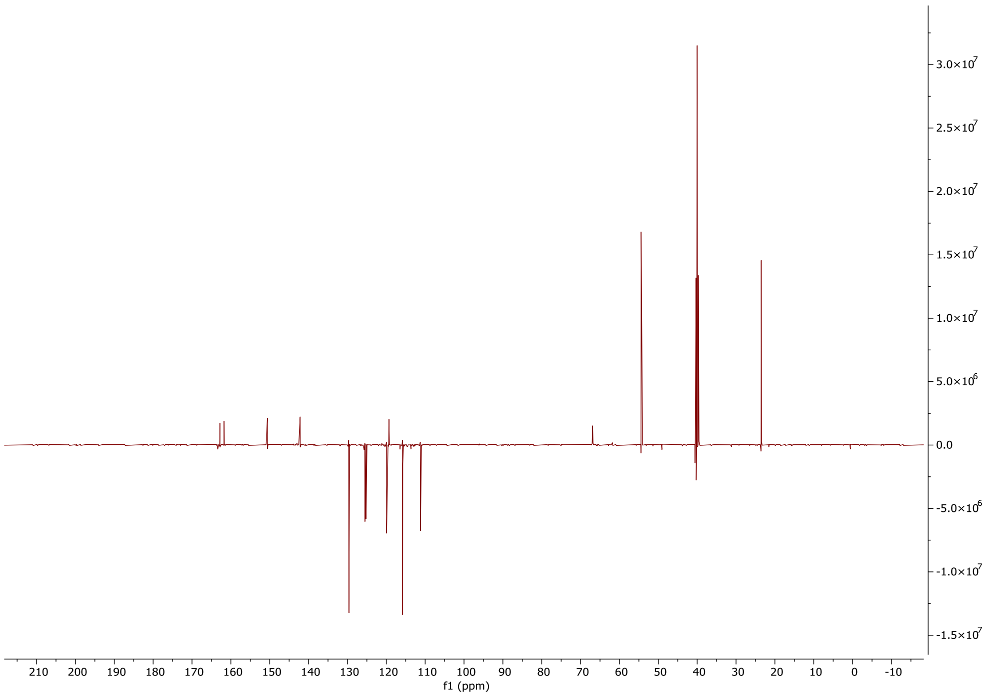

Figure S71. ^{13}C NMR (APT) spectrum of compound **39**

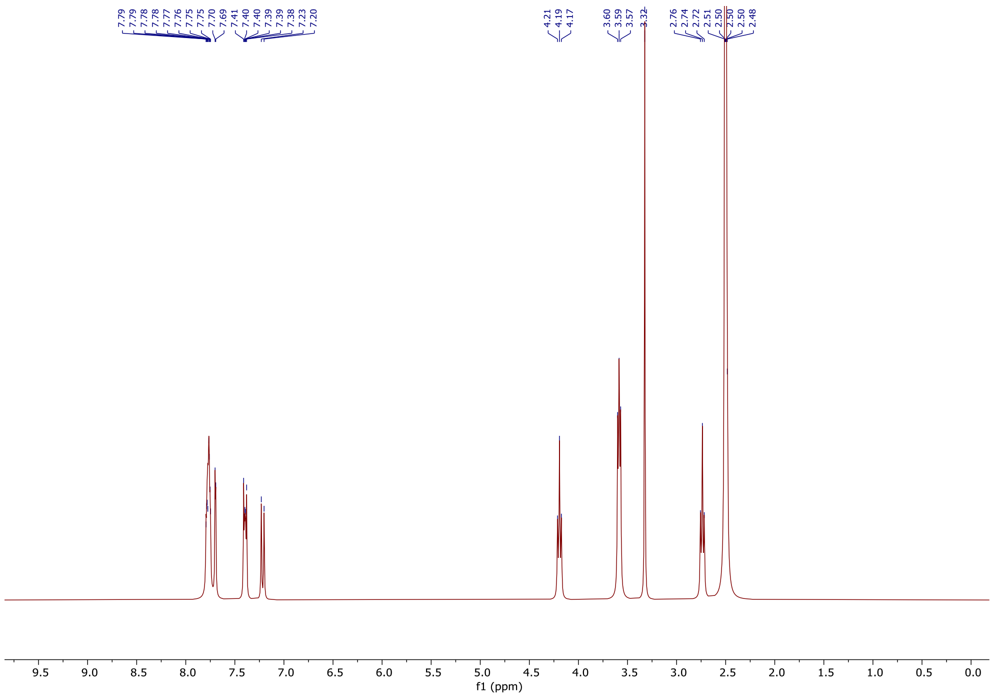

Figure S72. ^{1}H NMR spectrum of compound **40**

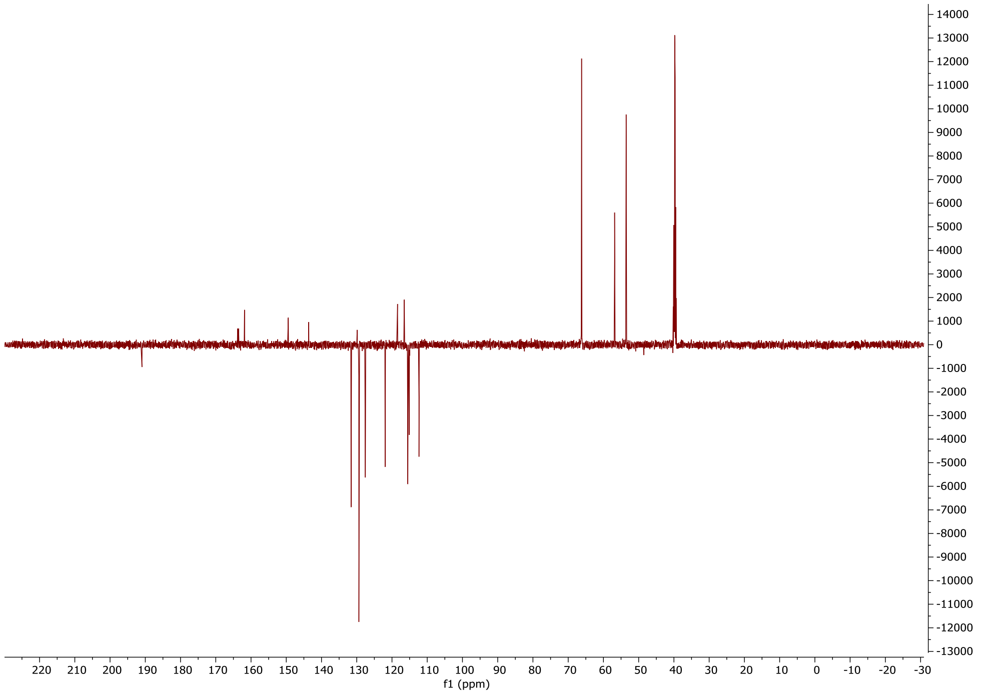

Figure S73. ^{13}C NMR (APT) spectrum of compound **40**

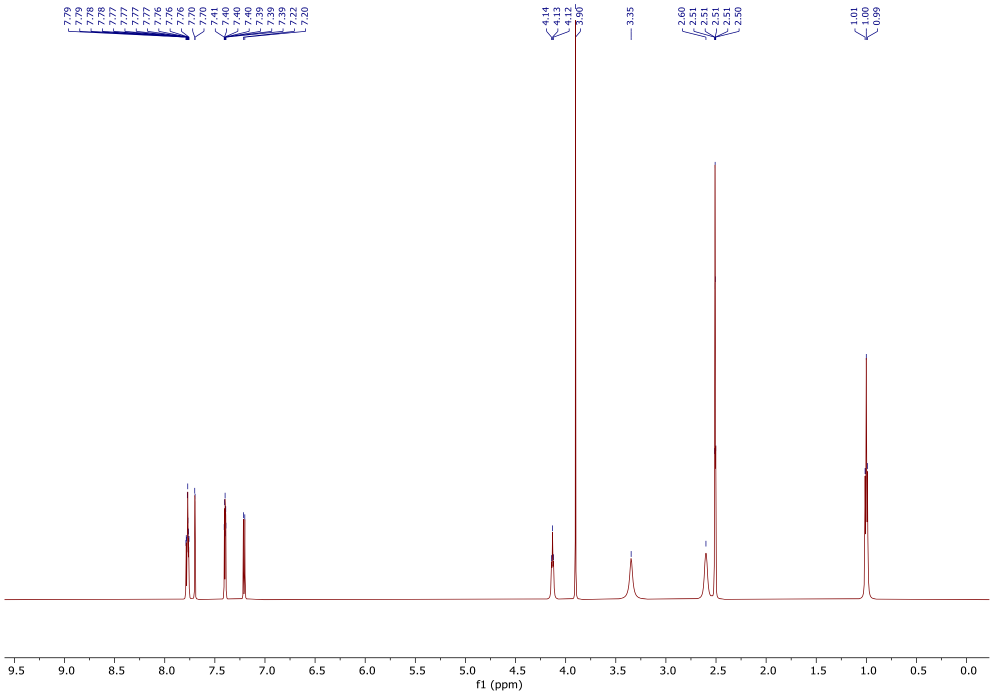

Figure S74. ^{1}H NMR spectrum of compound **41**

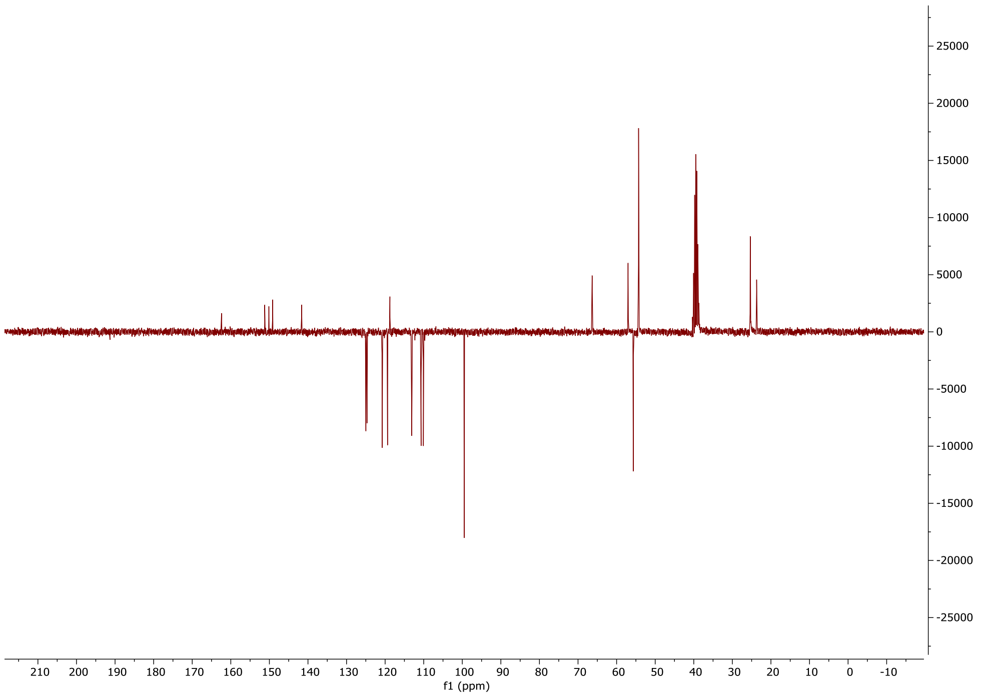

Figure S75. ^{13}C NMR (APT) spectrum of compound **41**

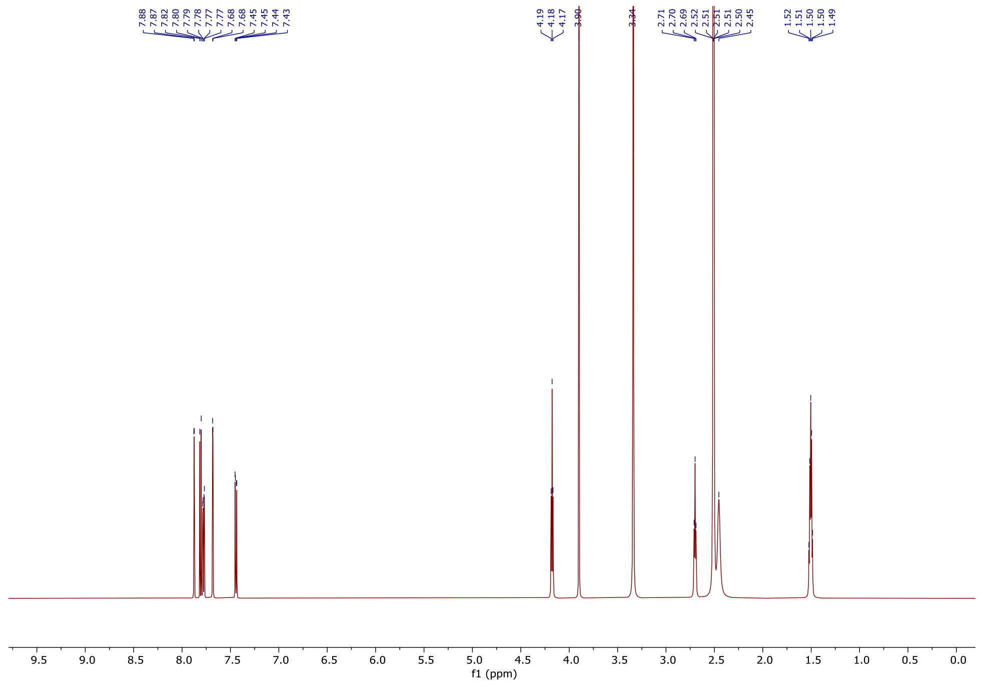

Figure S76. ^{1}H NMR spectrum of compound **42**

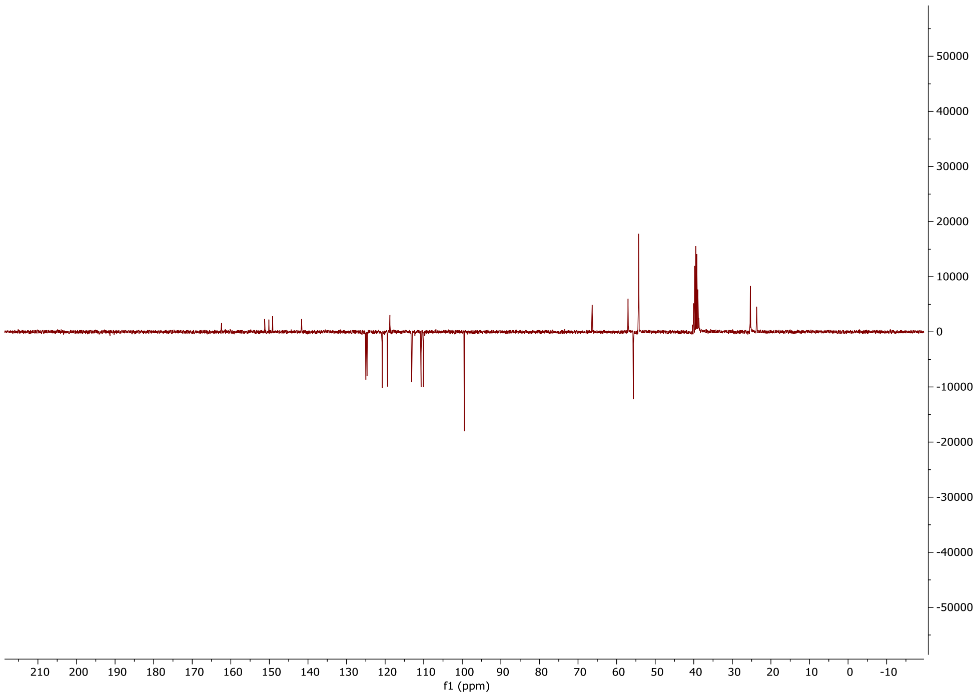

Figure S77. ^{13}C NMR (APT) spectrum of compound **42**

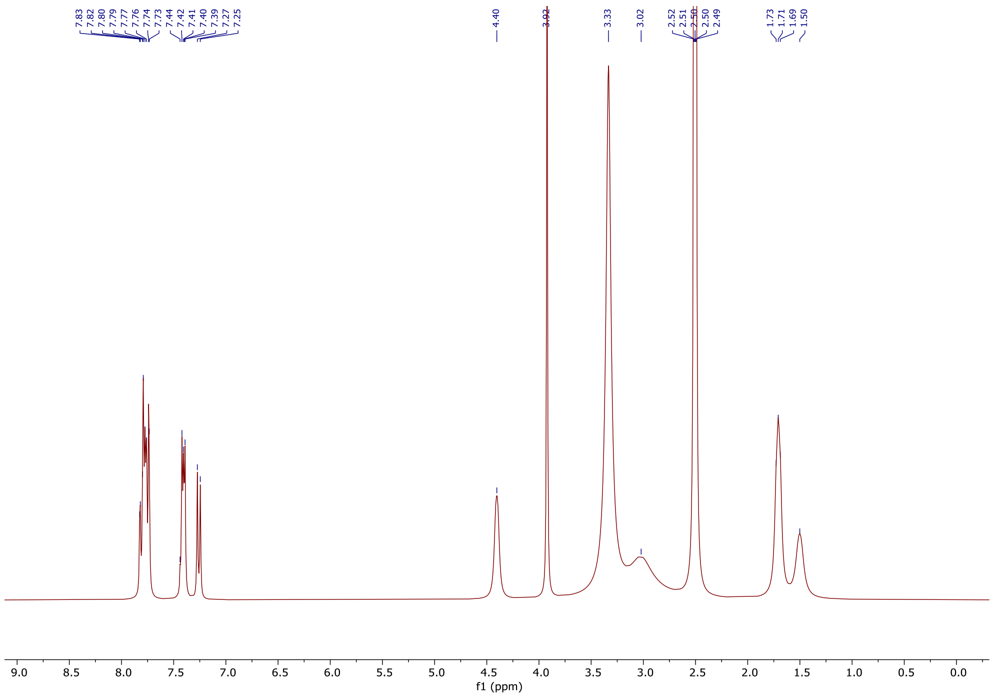

Figure S78. ^{1}H NMR spectrum of compound **43**

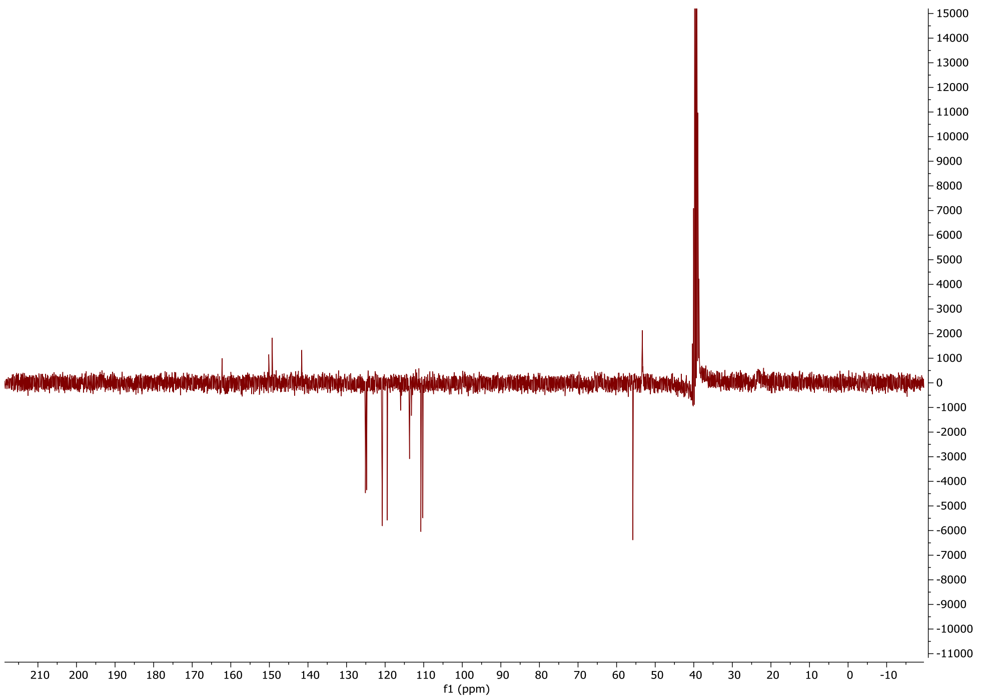

Figure S79. ^{13}C NMR (APT) spectrum of compound **43**

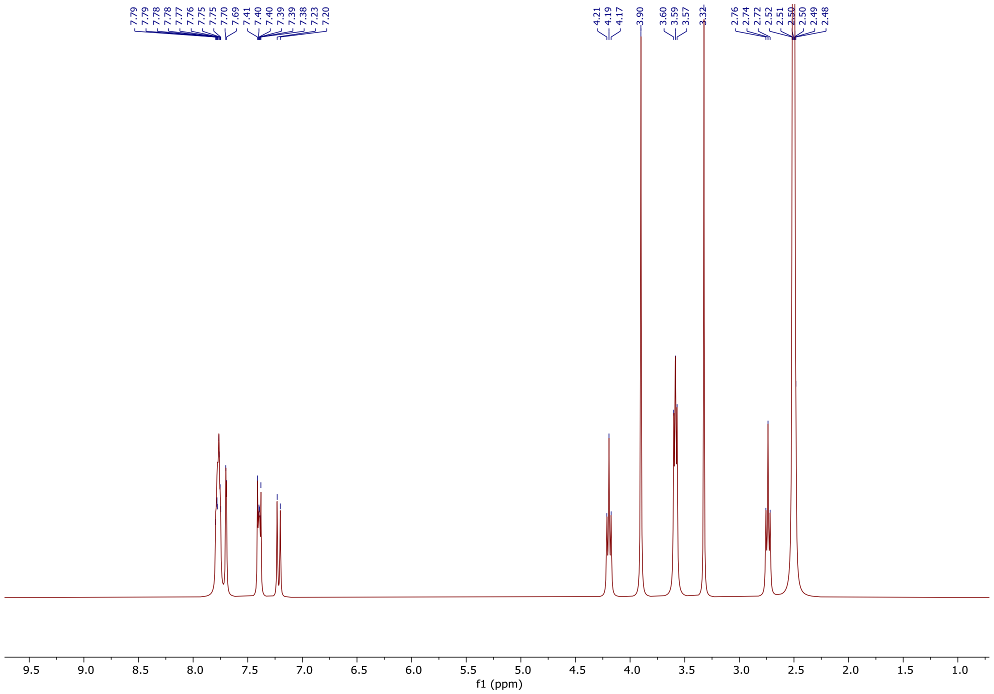

Figure S80. ^{1}H NMR spectrum of compound **44**

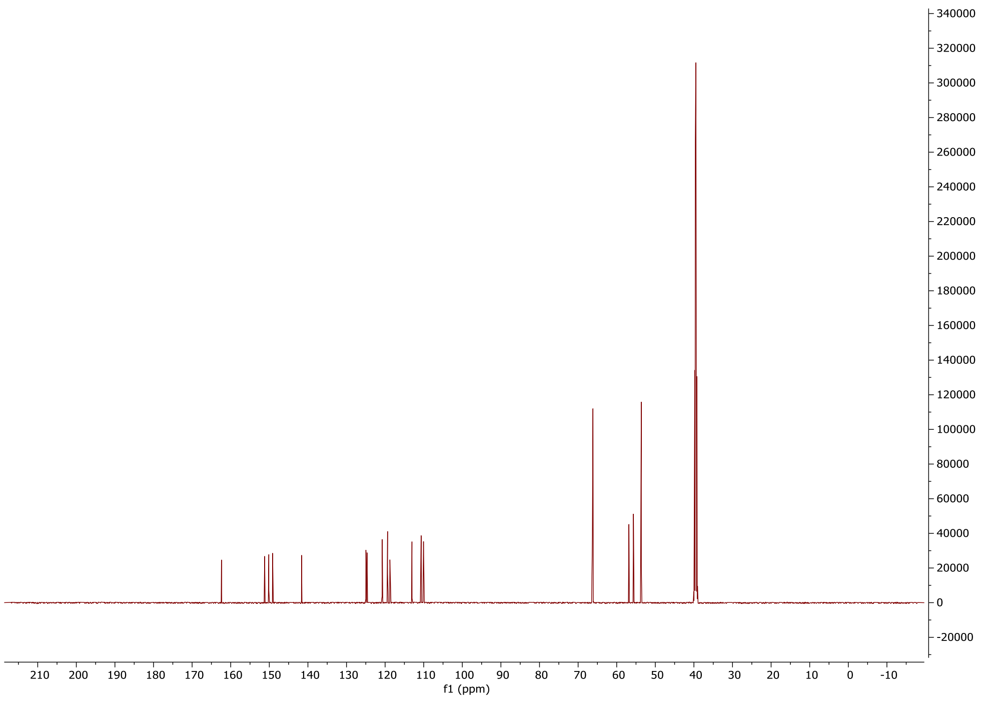

Figure S81. ^{13}C NMR spectrum of compound **44**

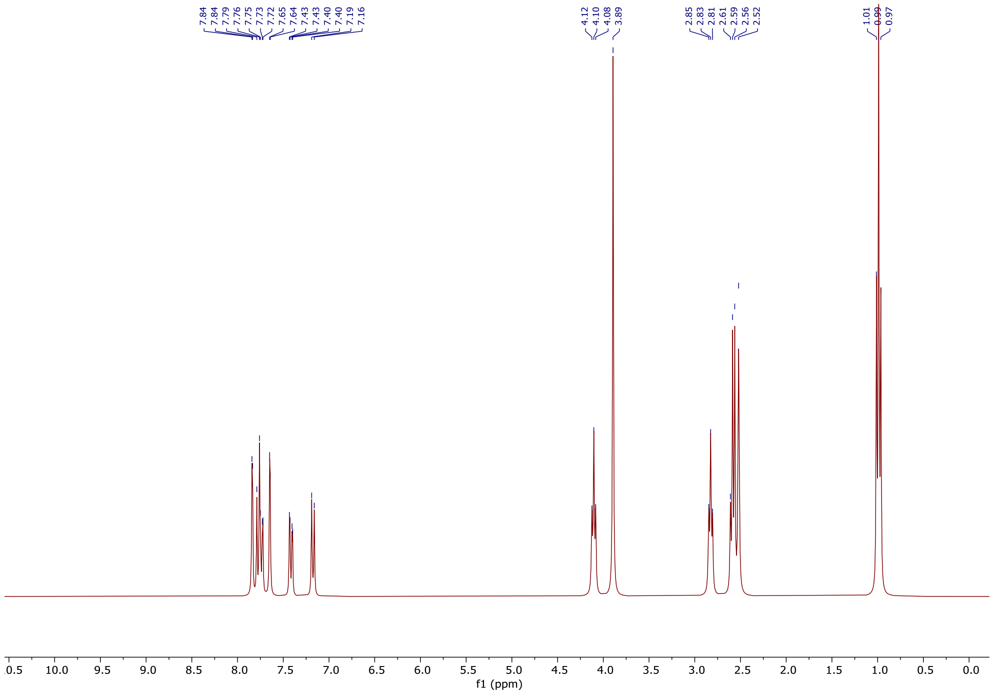

Figure S82. ^{1}H NMR spectrum of compound **45**

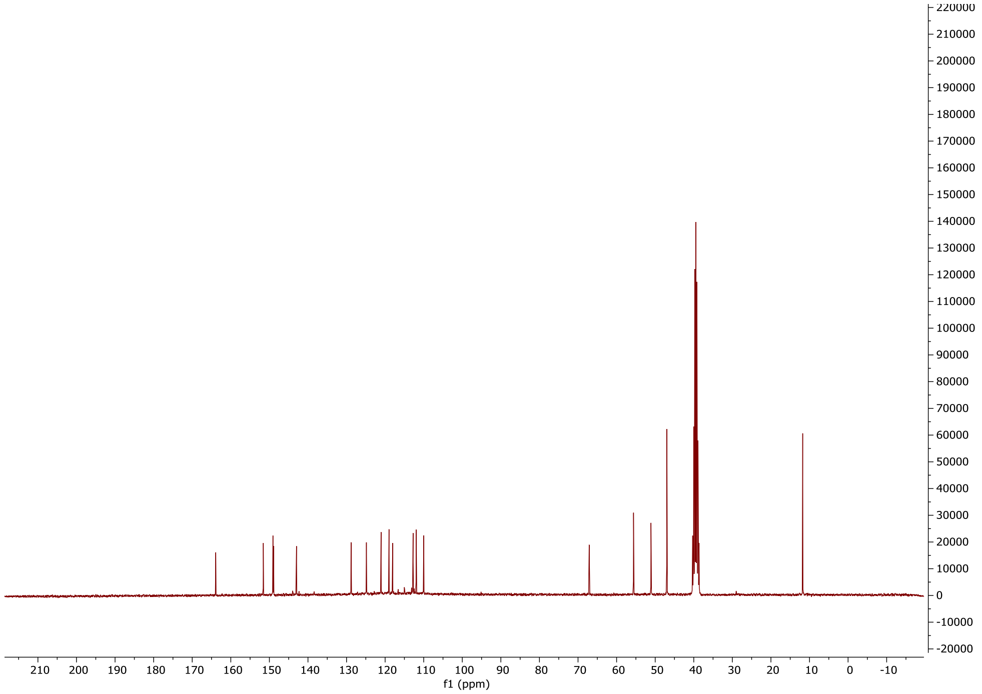

Figure S83. ^{13}C NMR spectrum of compound **45**

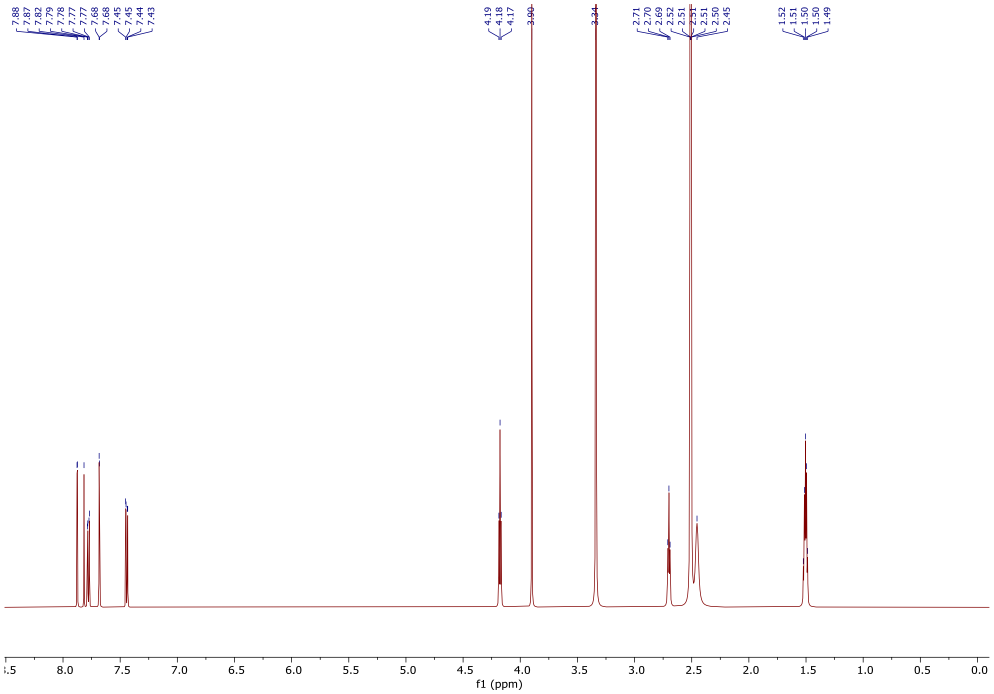

Figure S84. ^{1}H NMR spectrum of compound **46**

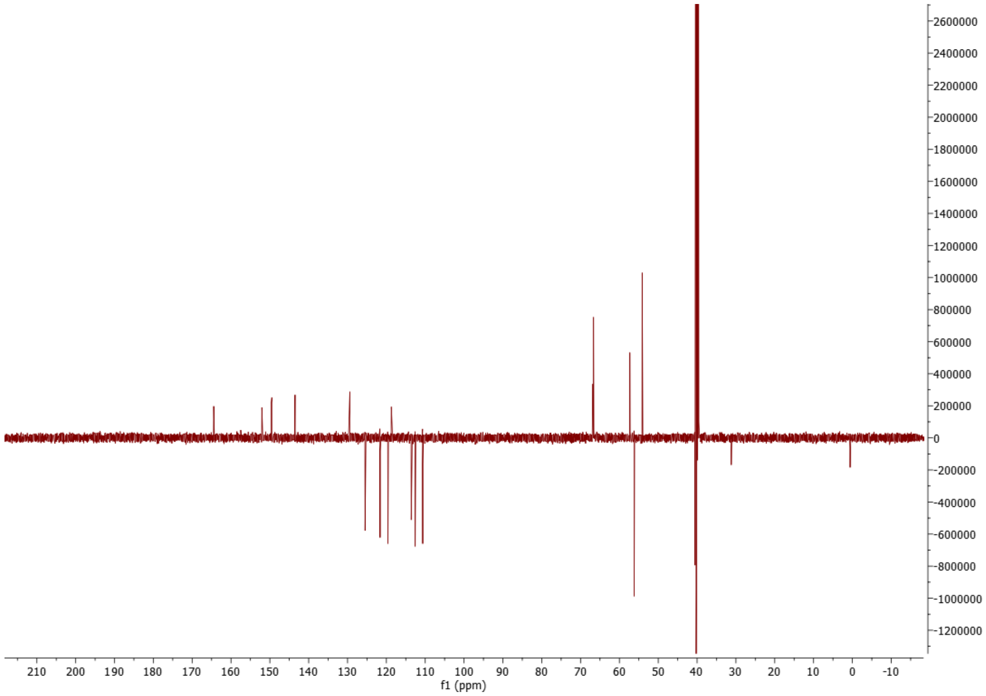

Figure S85. ^{13}C NMR (APT) spectrum of compound **46**

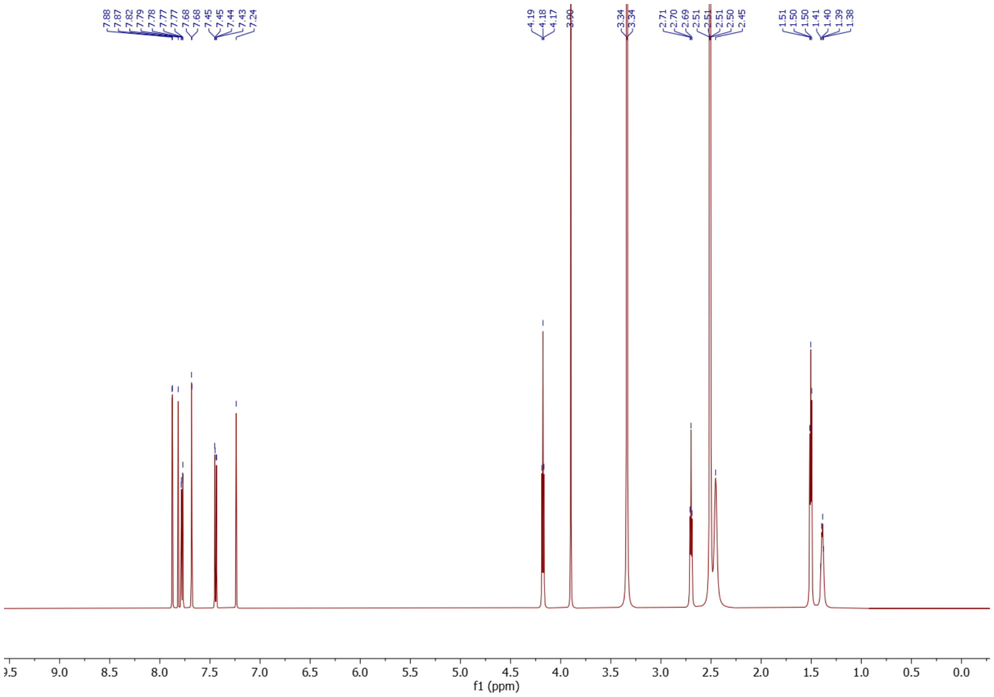

Figure S86. ^{1}H NMR spectrum of compound **47**

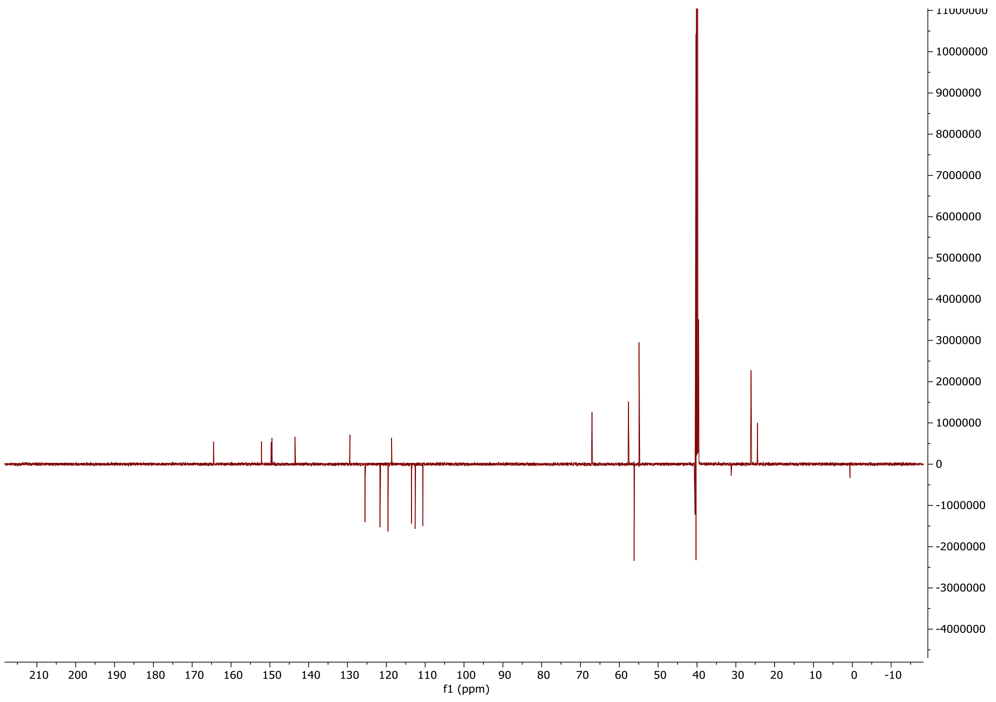

Figure S87. ^{13}C NMR (APT) spectrum of compound **47**

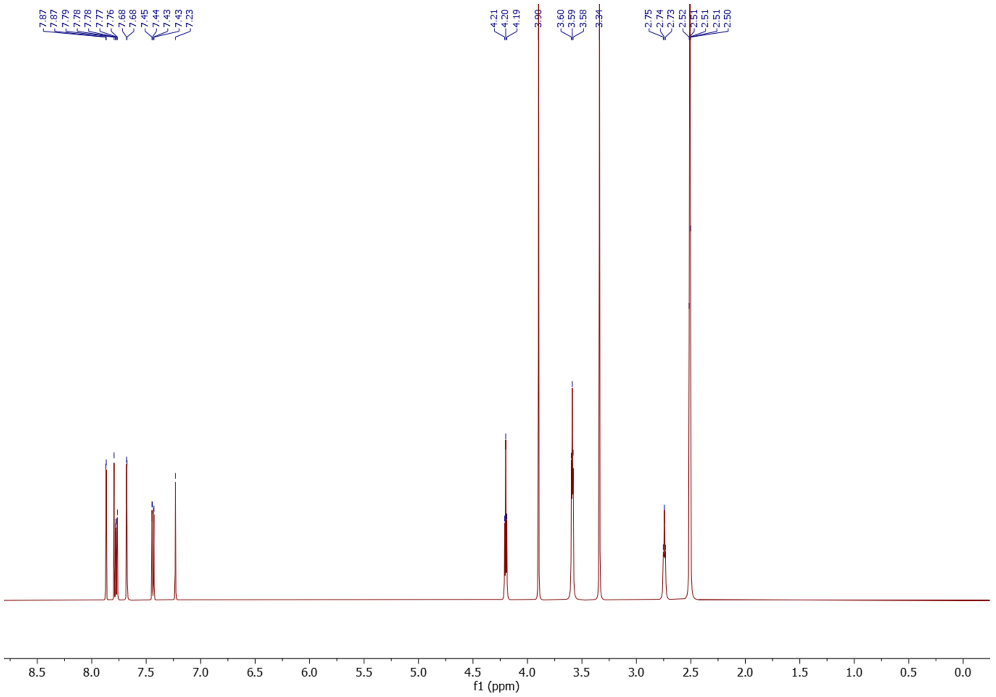

Figure S88. ^{1}H NMR spectrum of compound **48**

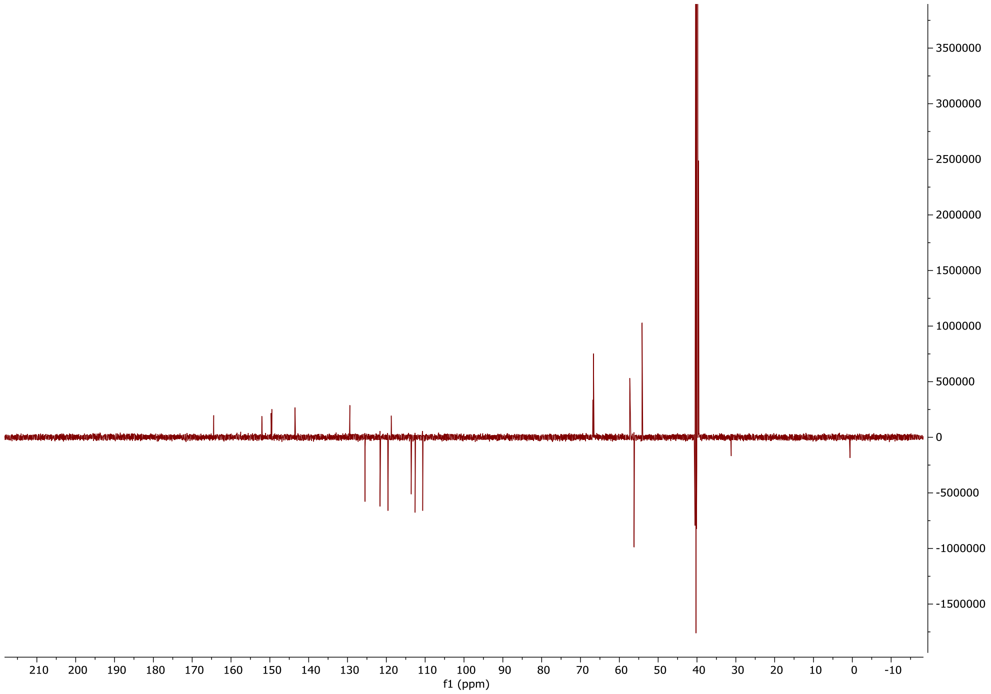

Figure S89. ^{13}C NMR (APT) spectrum of compound **48**

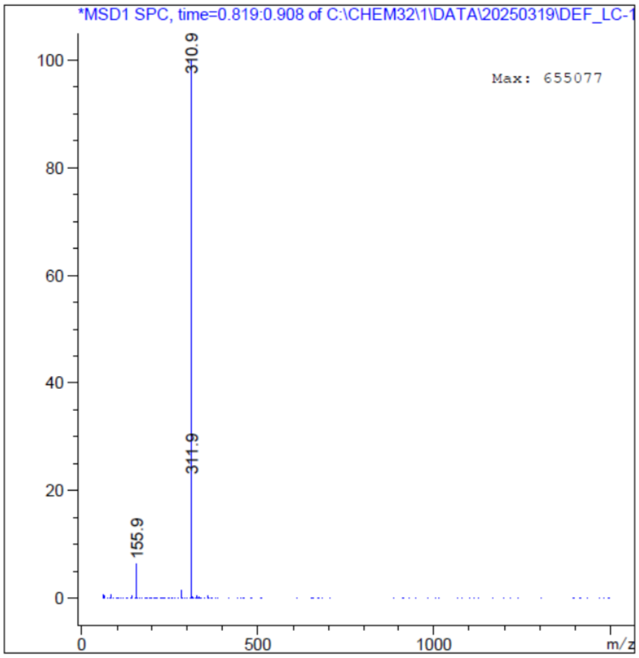

Figure S90. Mass spectrum of compund **29**

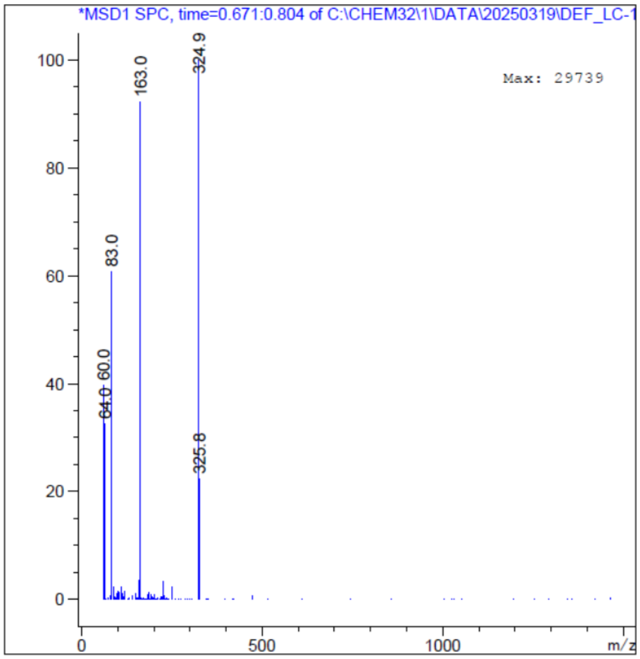

Figure S91. Mass spectrum of compound **32**

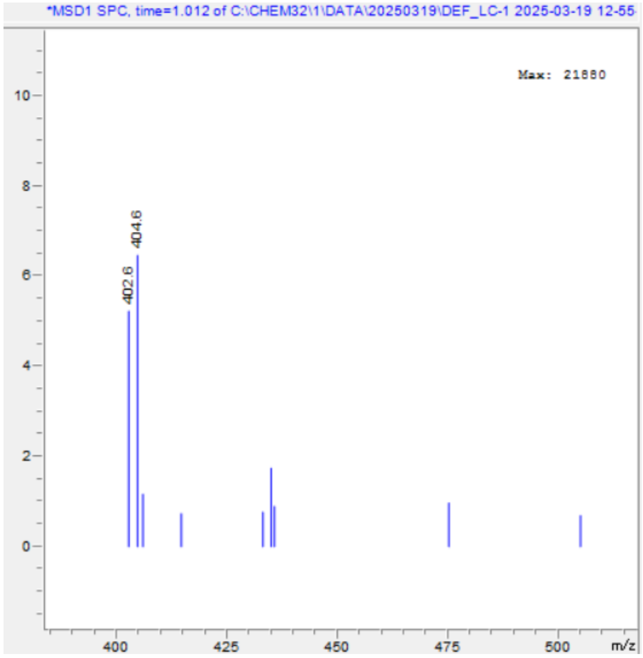

Figure S92. Mass spectrum of compound **40**

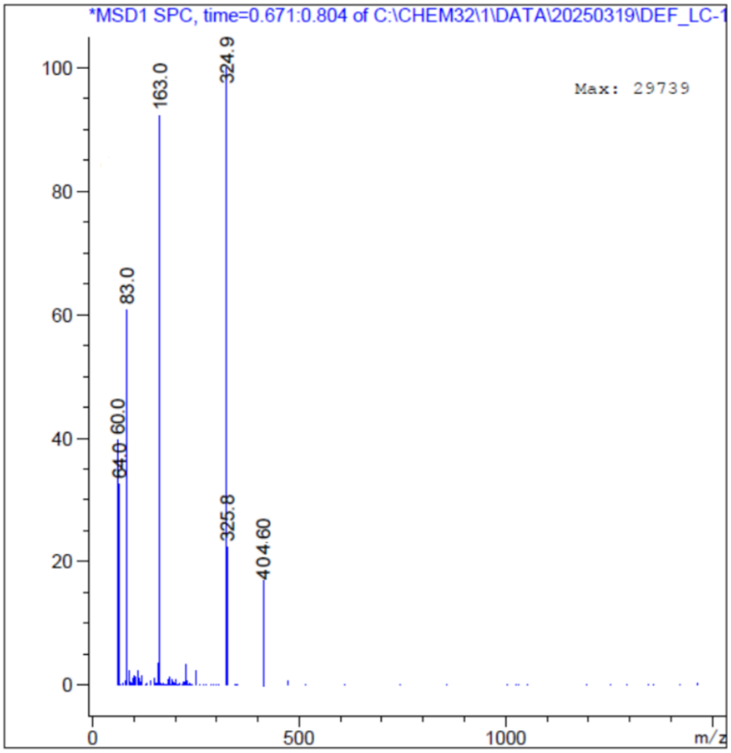

Figure S93. Mass spectrum of compound **44**
